# Single-cell RNA sequencing profiles drug activity within spatially engineered 3D cultures

**DOI:** 10.1038/s41467-026-74587-8

**Published:** 2026-06-19

**Authors:** Jessica J. King, Alireza Mowla, Jessica A. Kretzmann, Nishka Bhalla, Giselle Sugianto, Marck Norret, Ulrich D. Kadolsky, Munir Iqbal, Alka Saxena, Sebastian E. Amos, Yu Suk Choi, Brendan F. Kennedy, K. Swaminathan Iyer, Nicole M. Smith, Cameron W. Evans

**Affiliations:** 1 School of Molecular Sciences, The University of Western Australia, Perth, WA Australia; 2 BRITElab, Harry Perkins Institute of Medical Research, QEII Medical Centre and Centre for Medical Research, The University of Western Australia, Nedlands, Perth, WA Australia; 3 Department of Electrical, Electronic & Computer Engineering, School of Engineering, The University of Western Australia, Perth, WA Australia; 4 ARC Training Centre for Next-Generation Biomedical Analysis, The University of Western Australia, Perth, WA Australia; 5 Genomics WA, Harry Perkins Institute of Medical Research, The Kids Research Institute Australia, and The University of Western Australia, Nedlands, Perth, WA Australia; 6 School of Human Sciences, University of Western Australia, Perth, WA Australia; 7 Institute of Physics, Faculty of Physics, Astronomy and Informatics, Nicolaus Copernicus University in Toruń, Toruń, Poland; 8 School of Pharmacy and Pharmacology, University of Tasmania, Sandy Bay, TAS Australia

**Keywords:** RNA sequencing, Nanobiotechnology, RNA sequencing, Oligo delivery, Pharmaceutics

## Abstract

Spatial transcriptomic techniques provide a wealth of information useful in guiding drug development, while three-dimensional (3D) cell cultures have demonstrated power in accelerating drug approvals. However, techniques for robust spatial analysis of 3D cultures are limited. Here, we present a transfection-based method for constructing cellular spheroids through a layer-by-layer approach, in which DNA barcodes encode the spatial positioning of cells. Our technique facilitates multiplex single-cell RNA sequencing, providing spatial maps of gene expression and drug response, while correlative imaging reveals the locations of barcoded cell populations and quantifies local tissue elasticity. We show that model HeLa 3D spheroids display heterogeneous responses to drugs, which may arise through diffusion gradients of the drug, or from differences in metabolism, nutrient supply, and cellular stressors. The ability to create spatially encoded cellular assemblies may help to reveal spatial variation in gene expression within 3D culture models.

## Abstract (web summary)

King and colleagues use scTECH-seq to attain spatial mapping of single-cell gene expression and drug response in three-dimensional cultured cell spheroids. Spatial expression analysis provides insight into heterogeneous drug responses in 3D culture models.

## Introduction

Organ-on-a-chip platforms^1^ demonstrate the power of three-dimensional (3D) cultures to accelerate animal-free clinical drug approvals. While in vivo animal studies are more biologically relevant than two-dimensional (2D) monolayer cell cultures for drug screening^2^, animal models can be costly, time-consuming^3^, and often fail to translate to humans^4–6^. As an attractive 3D intermediary between homogeneous 2D cell cultures and highly complex tissue samples, 3D cultures have been successfully implemented in cancer and tumour research, and drug screening applications^7^. Spheroids are readily scalable 3D cell cultures that form sphere-like masses via cell-to-cell interactions, which may adopt a structural and spatial complexity similar to that found in vivo^8^. 3D cultures can more faithfully represent the heterogeneous cellular responses found in animal models, reflecting gradients in oxygen and nutrient supply, metabolic rate, and cell proliferation.

The ability to engineer 3D cultures with pre-coded spatial information will help to enrich drug screening and gene expression data. Understanding how cell position relates to transcriptomic changes could provide insight into how drug penetration, differences in the cell microenvironment, or cell–cell interactions contribute to drug response or disease progression. Characterising gene expression in 3D requires spatially-resolved single-cell RNA-seq (scRNA-seq) data^9^ to capture transcriptomic profiles in the context of cellular organisation^10^. At the time this work was initiated, 10X Genomics^11,12^ and NanoString Technologies^13^ spatial analysis platforms were limited to a spatial resolution of ~50 μm, precluding capture of individual-cell gene expression profiles^13,14^. In the context of 3D culture models, which are typically only a few hundred microns in size, these spatial analysis platforms can be technically challenging to implement^15,16^. Subsequent platforms, including Visium HD (2 μm barcoded arrays) and Xenium, now achieve single-cell or subcellular resolution within 2D tissue sections. However, applying these technologies to 3D culture systems such as spheroids requires serial cryosectioning, per-section capture or imaging, and computational reconstruction of radial architecture, a workflow that is both cost-prohibitive at the scale of typical spheroid experiments (drug screens, time courses, perturbation panels) and ill-suited to resolving the concentric layer organisation that defines spheroid biology, including proliferative rim, intermediate zone, and hypoxic core. Furthermore, the low throughput limits analysis of 3D architectures^17^. A platform for the study of gene expression that captures profiles at the single cell level while also maintaining critical spatial information will help to bridge the divide between homogeneous 2D cell culture and animal models for analysing 3D cultures.

Here, we construct multi-layered spheroids in which short barcode oligonucleotides (SBOs) encode spatial information and facilitate sample 5′-multiplexing for scRNA-seq, a technique we term single cell transfection-enabled cell hashing (scTECH-seq)^18^. Herein, we adopt our scTECH-seq method, using SBO–fluorophore conjugates to enable visualisation of barcoded cells in 3D spheroid cultures, prior to scRNA-seq. We demonstrate the construction of barcoded HeLa spheroids through a layer-by-layer assembly process and spatially profile drug-induced transcriptional responses within 3D cell cultures. The use of fluorescent barcodes also supports correlative imaging analysis using a recently reported mechano-microscopy technique^19^ to measure tissue elasticity within 3D cultures.

## Results

### scTECH-seq enables visualisation and single-cell sequencing of multi-layered spheroids

**Fig. 1:**
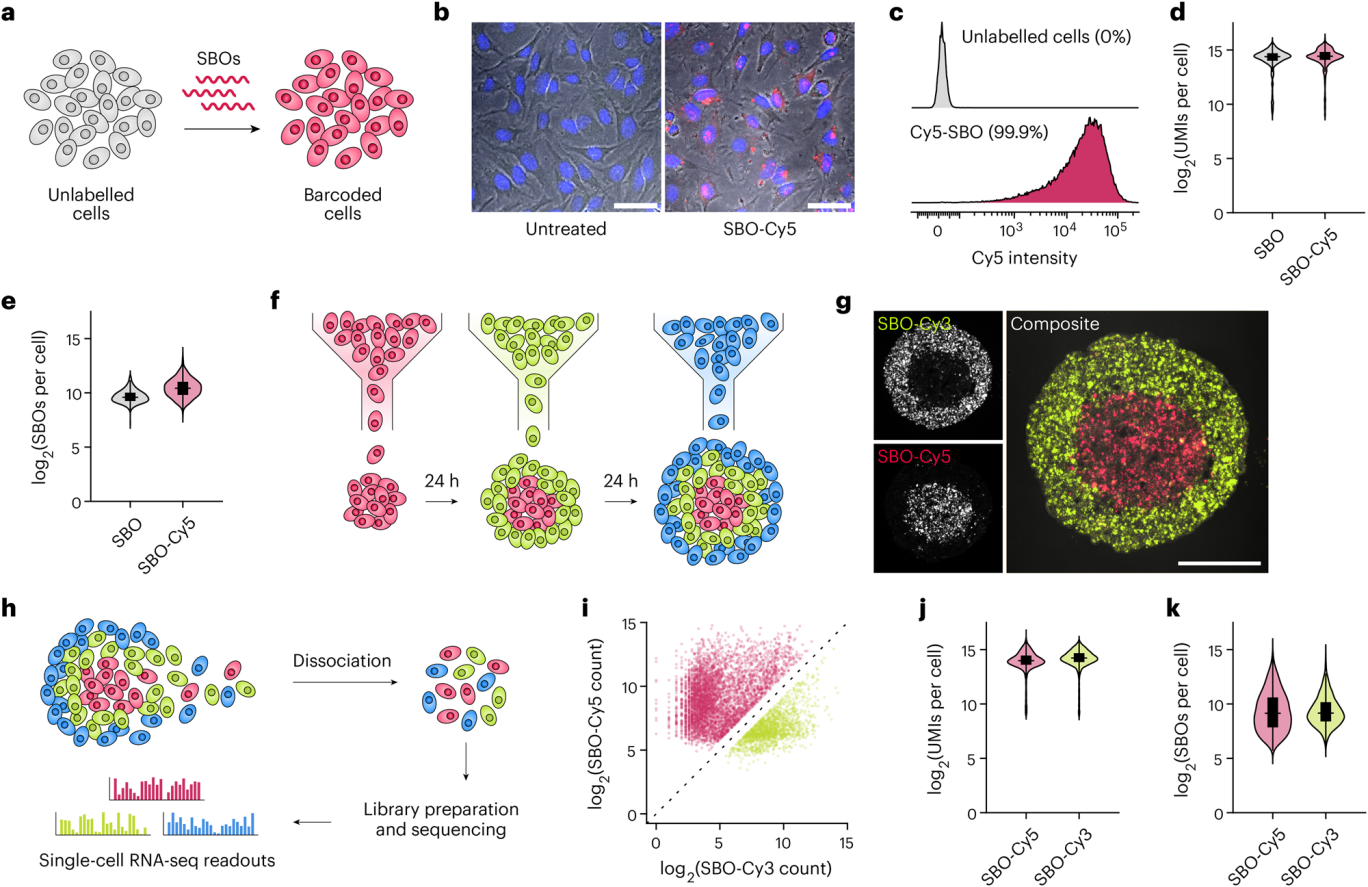
Single-cell transcriptomics of multi-layer spheroids. **a** Cells are transfected with short barcode oligonucleotides (SBOs) in culture to produce populations of ‘barcoded’ cells. **b** Fluorescent microscopy shows SBO (red) uptake in HeLa cells; counterstained nuclei (blue) with phase contrast overlay. Scale bar 50 μm. **c** SBO uptake efficiency was measured using flow cytometry, with >99% cells positive for SBO fluorescence (two biological replicates, *n* = 5). **d** Violin plot showing SBO fluorophore conjugation does not reduce RNA counts per cell for SBOs with (*n* = 595 cells) or without (*n* = 1239 cells) Cy5 modification. Groups were compared by one-tailed *t*-test (*p* = 0.9995). **e** Violin plot shows SBO fluorophore conjugation does not reduce SBO counts per cell with (*n* = 595) or without (*n* = 1239) Cy5 modification. Groups were compared by a one-tailed *t*-test (*p* = 1). **f** Schematic of the experimental approach for barcoding cell populations and growing spheroids using a layered approach. Different colours represent unique SBO sequences, each encoding a spatial region of interest. **g** Representative image of spheroid cross-section showing two cell layers, labelled with Cy5- (red) and Cy3- (yellow) conjugated SBOs. Scale bar 200 μm. Result representative of *n* = 2 spheroids in independent biological replicates. **h** Schematic of sequencing preparation process. Spheroids are dissociated into a single-cell suspension and undergo encapsulation and reverse transcription using the 10X Chromium Controller. Data is retrieved and analysed using CellRanger and Seurat. **i** Cy5- and Cy3- modifications do not interfere with SBO sequencing and deconvolution using D-score. **j** Violin plot shows a similar number of RNA counts per cell for Cy5-SBO (*n* = 4783 cells) and Cy3-SBO (*n* = 1717 cells). **k** Violin plot shows a similar number of SBO counts per cell for Cy5-SBO (*n* = 4783 cells) and Cy3-SBO (*n* = 1717 cells). Data for (**d**, **e**) and (**j**, **k**) were derived from one sequencing experiment; box and whisker plot overlays display median (horizontal line), interquartile range (IQR, box) and 1.5×IQR (whisker), while values outside 1.5×IQR are not plotted.

scTECH-seq uses SBOs as described by 10X Genomics, which we modified through addition of a fluorophore label at the 5′ end to enable SBO visualisation and quantification in cells (Supplementary Fig. 1, Supplementary Table 1). In this scheme, each individual cell is assigned a unique 10X barcode during single-cell library preparation via the 10X Genomics Chromium platform, while SBOs serve as feature barcodes that distinguish different samples of cells. HeLa cells were transfected for 4 h with dye-conjugated-SBOs (Fig. 1a) using 23 pmol SBOs per 150,000 cells, based on previous reports^18,20^. SBO uptake was visualised with fluorescence microscopy (Fig. 1b), and flow cytometry quantification of fluorescent SBOs indicated >99.9% labelling efficiency (Fig. 1c). SBOs were inherited by daughter cells following cell division and had an apparent half-life in HeLa cells of 28 ± 4 h (Supplementary Fig. 2), which matches the cell population doubling time and is consistent with the number of SBOs per cell halving with each cell division. A comparison of scRNA-seq using fluorophore-modified versus unmodified SBOs showed no reduction in RNA transcripts per cell, gene counts, or SBO numbers per cell (Supplementary Tables 2–4) when using Cy5-modified SBOs compared to unmodified SBOs (Fig. 1d, e). We used SBO-labelled cells to construct layered spheroids, in which each layer comprised cells transfected with a unique SBO (Fig. 1f). The advantage of a layer-by-layer spheroid is that it allows for engineering of a 3D culture with a controlled spatial organisation of barcoded cells; a randomised spheroid would contain different barcoded cells randomly distributed throughout, which would not allow analysis of different spatial regions.

Confocal microscopy of spheroid cryosections enabled visualisation of two distinct barcoded layers (Fig. 1g). Subsequent dissociation of spheroids (Fig. 1h) and quantification of SBOs via flow cytometry confirmed that barcodes were not lost or exchanged between individual cells (Supplementary Fig. 3). Two-layer spheroids tagged with Cy3- and Cy5-SBOs were dissociated, pooled, and sequenced, whereby SBOs also facilitated demultiplexing of scRNA-seq data (Fig. 1i). For the two layers, we observed similar numbers of RNA transcripts, genes, and SBO reads per cell (Fig. 1j, k, Supplementary Tables 5–7), demonstrating capacity to construct and visualise SBO-labelled architectures using unmodified or fluorophore-labelled SBOs without compromising sequencing reads or demultiplexing integrity. Analysis of gene expression in two-layer spheroids can be found in Supplementary Notes and Supplementary Figs. 4–6.

### Barcoding facilitates examination of spatial transcriptional differences in multi-layer spheroids

**Fig. 2:**
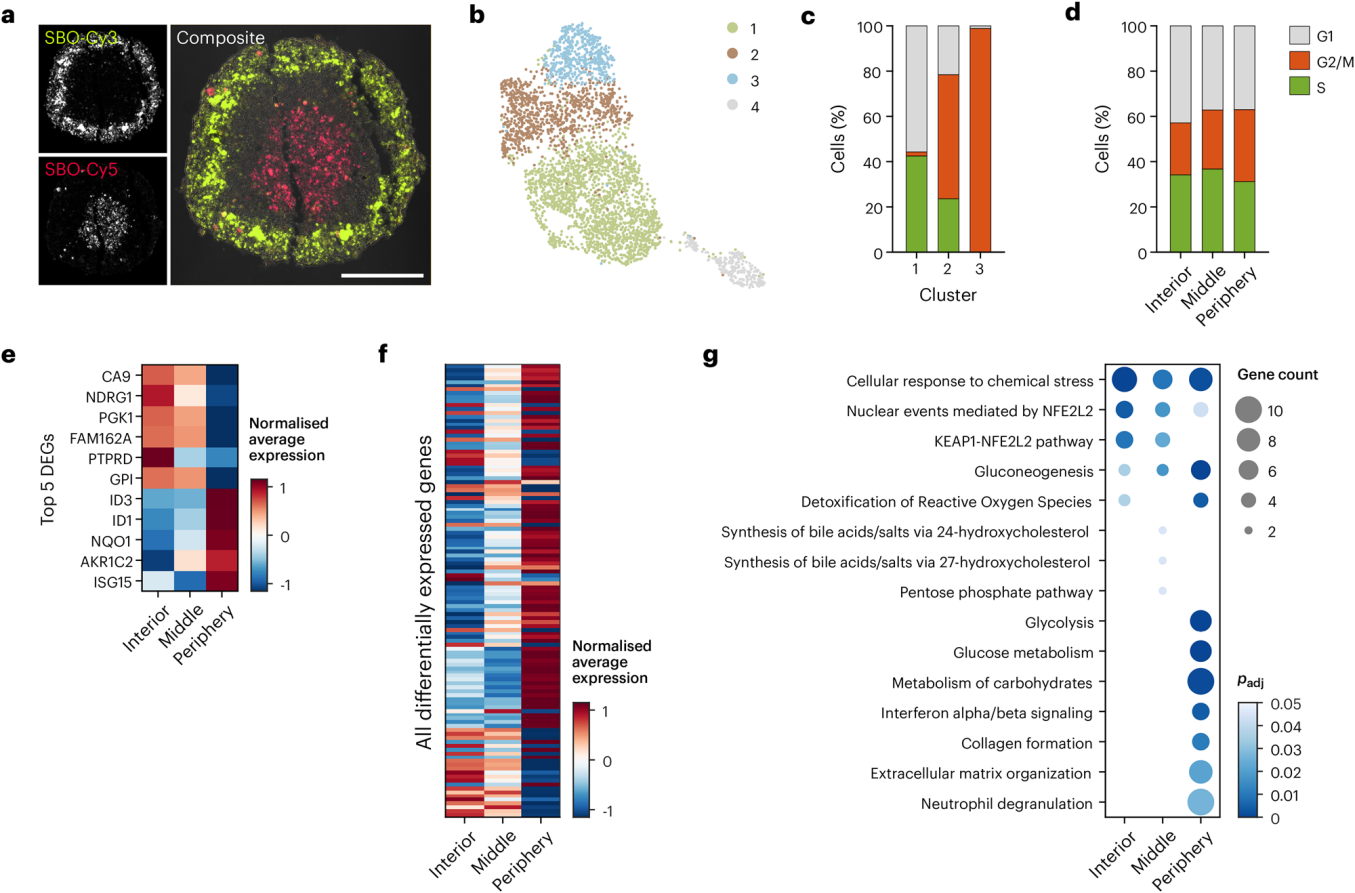
Multi-layer spheroids highlight spatial differences in gene expression related to cell cycle, metabolism, and nutrient supply. **a** Representative image of three-layer spheroid. Cells within the core (first layer) are labelled with Cy3-SBO (yellow), the middle layer contains cells barcoded with an SBO that was not conjugated to a fluorophore, and cells on the periphery are labelled with Cy5-SBO (red). Scale bar 200 μm. Result representative of *n* = 2 spheroids in independent biological replicates. **b** Uniform Manifold Approximation and Projection (UMAP) representation of the single cells recovered from three-layer spheroids. Cells are coloured by gene expression cluster. **c** Quantification of cell cycle markers within each of the three main clusters identified in (**b**). Cluster 3 principally contains cells undergoing mitosis (G2/M), while cluster 1 displays virtually no markers of G2/M phase. Thus, the main differences in gene expression across untreated spheroids are simply due to the cell cycle. **d** Distribution of cell cycle markers according to the spheroid layer shows that cell cycle progression is relatively consistent between each of the three layers. **e** Normalised average expression levels of the top 5 differentially expressed genes (DEGs, |log_2_ fold change|> 0.25, *p*_adj_ < 0.05) across three-layer spheroids. **f** Normalised average expression levels for all DEGs (|log_2_ fold change|> 0.25, *p*_adj_ < 0.05) across three-layer s*p*heroids. **g** Dot plot showing gene ontology (biological process) pathways enriched within each layer of three-layer spheroids. The dot size indicates the gene counts expressed in each pathway, while the colour indicates the adjusted *p*-value. In panels e–g, statistical significance for differential gene expression (*p*_adj_) was reported by Seurat using a non-parametric Wilcoxon rank sum test with Bonferroni correction. Source data are provided as a Source Data file.

Extending the approach described above, we constructed spheroids from three populations of HeLa cells, each of which had been labelled with a unique SBO. Barcoded cell populations were added each day over a 3-day period (Fig. 2a), creating three defined layers. Spheroids were dissociated and sequenced as above. Transcript, gene, and SBO counts are summarised in Supplementary Tables 9–11. Using the D-score tool for demultiplexing scRNA-seq data^21^, 82.5% of cells were unambiguously assigned to one of the three SBOs used, deconvolving cell populations by SBO and hence also by spatial organisation. Cells were clustered into four groups by gene expression during analysis (Fig. 2b). Many of the differentially expressed genes (DEGs) between clusters could be attributed to cell cycle progression, and analysis of the cells in clusters 1–3 confirmed substantial differences in the number of cells in G1, S, and G2/M phase (Fig. 2c). A subpopulation of cells (cluster 4, representing 7.0% of total cells) displayed hallmarks of cell death, including deregulation of housekeeping genes including *ACTB*, *GAPDH*, *HPRT1*, and *EIF* family members. This cluster contained cells from each layer (from 6 to 12% of the cells in each layer, representing 7.0% of all cells), which may represent cells that were damaged during spheroid dissociation, and these were excluded from further consideration.

Spatial analysis of gene expression revealed 117 DEGs (|log_2_ fold change|> 0.25, *p*_adj_ < 0.05) between the interior, middle and peripheral layers of three-layer spheroids. Examining cell cycle with respect to each of the three layers within the spheroids rather than cluster showed a similar distribution of cell cycle markers across the layers (Fig. 2d), while further examination of the top DEGs across the three layers revealed markers of growth, metabolism, and waste processing (Fig. 2e). Despite some indicators of cell stress and death within a small subpopulation of cells, the principal transcriptomic differences in multilayer spheroids were the result of growth and cell division. Expression profiles in spheroid interior regions confirmed some enrichment of apoptotic and necrotic pathways (Supplementary Fig. 7).

The top 5 DEGs identified in each layer of three-layer spheroids are shown in Fig. 2e. Of these, 8/11 genes were also found to be differentially expressed in two-layer spheroids (Supplementary Table 8). Qualitatively, across all DEGs, the middle layer tended to display average expression levels that were intermediate between the interior and periphery (Fig. 2f), supporting our hypothesis that gradients in gene expression patterns could be spatially defined. Pathway analysis using DEGs was conducted (Fig. 2g); KEAP1-NFE2L2 pathways and NFE2L2 nuclear events were upregulated in the interior, consistent with an increase in oxidative stress, while glycolysis, glucose metabolism, and ECM formation and organisation processes were more prevalent in the peripheral layer. These results are consistent with the current understanding of spheroid cultures in which there is a gradient in metabolic rate, nutrient supply, and oxygen dependent on cell depth, and dense cell-to-cell contact and suppressed cell proliferation in the spheroid core^22^.

### In vitro analysis of irinotecan-treated spheroids

**Fig. 3:**
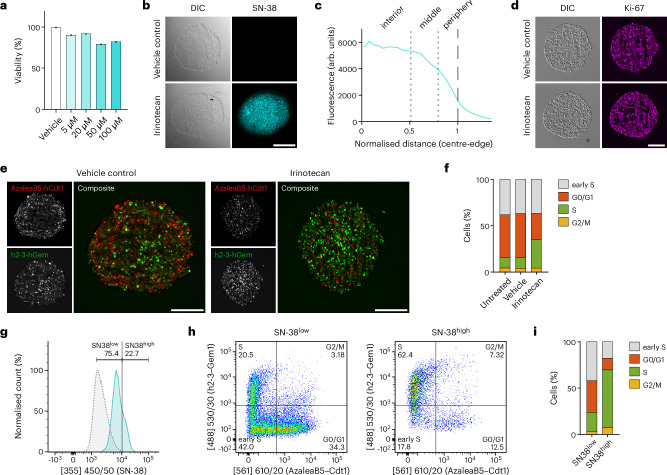
Topoisomerase I inhibition alters cell cycle state in three-layer HeLa spheroids treated with irinotecan for 24 h. **a** Percentage of live cells from dissociated spheroids after 24 hours of irinotecan treatment using propidium iodide. The percentage of live cells (mean ± standard deviation) was calculated via flow cytometry and normalised to the vehicle control (spheroids treated with 0.1% v/v DMSO, ‘vehicle’). 50,000 single cells were collected per sample. **b** Epifluorescence microscopy images of spheroid cryosections in which fluorescence was used for SN-38 detection. Scale bars 200 μm. Result representative of *n* = 4 spheroids. **c** Quantification of SN-38 fluorescence across the diameter of the spheroid cross-section shown in (**b**). **d** Ki-67 staining of spheroid cryosections. Scale bars 200 μm. Result representative of *n* = 4 spheroids. **e** Representative fluorescence images of Fucci spheroid sections show that irinotecan arrests cells in S phase with more cells expressing h2-3–hGem (green). AzaleaB5–hCdt1 fluorescence is indicative of G1 phase, h2-3–hGem fluorescence is indicative of S phase, and cells expressing both markers indicate G2/M phase. Scale bars 200 μm. Result representative of *n* = 2 spheroids in independent biological replicates. **f** Flow cytometry quantification of dissociated Fucci spheroids shows an increase in cells in S phase after irinotecan treatment. Spheroids (*n* = 8) per condition were dissociated and 50,000 single cells were processed during flow cytometry. **g** Analysis of cell cycle arrest as a function of drug concentration. Fucci HeLa spheroids were treated with irinotecan, dissociated, and analysed by flow cytometry. Irinotecan-treated cells (blue solid curve) were separated into SN-38^high^ and SN-38^low^ populations for analysis. Cells from untreated control spheroids are shown as a grey dotted line. >30,000 cells were analysed. **h** Scatter plots for the two populations shown in (**g**) based on the expression of each of the Fucci markers. The percentage of cells in each quadrant is shown. **i** Quantification of cell cycle by Fucci in SN-38^high^ and SN-38^low^ cells as shown in (**h**) confirms that the proportion of cells arrested in S phase correlates with the effective concentration of SN-38 (active metabolite). Source data are provided as a Source Data file.

To explore the spatial analysis of gene expression in response to an experimental stimulus, three-layer spheroids were treated with irinotecan. Irinotecan is a prodrug that is used for various cancers^23–26^, particularly colon cancer. The primary target of irinotecan is DNA topoisomerase I, which relaxes supercoiled DNA during replication and transcription^27,28^. Carboxylesterases metabolise irinotecan to ethyl-10-hydroxycamptothecin (SN-38)^29,30^, which is a fluorescent metabolite with excitation at 360 nm and emission at 420 nm, making it an ideal candidate to monitor intracellular drug penetration within spheroids. SN-38 stabilises topoisomerase I–DNA complexes, resulting in double-stranded DNA breaks and activation of apoptotic pathways^28^. Using spheroids with three layers, we tested different concentrations of irinotecan prepared in 0.1% v/v DMSO for 24 h, as well as a 0.1% v/v DMSO control (hereafter referred to as ‘vehicle control’) and an untreated control (Supplementary Fig. 8). The percentage of live cells was quantified via flow cytometry using propidium iodide staining (Fig. 3a). For subsequent studies, 50 μM irinotecan was selected.

Imaging of SN-38 revealed a relatively homogenous distribution throughout all spheroid layers (Fig. 3b), with a reduction in fluorescence intensity towards the periphery (Fig. 3c), however we speculate that this may have arisen due to drug diffusion and loss during sample processing. Ki-67 was used to evaluate proliferation in spheroids before (vehicle control) and after irinotecan treatment. Spheroids possessing an apoptotic or necrotic core were expected to have fewer Ki-67^+^ cells, but immunofluorescence of spheroid sections indicated that Ki-67^+^ cells were distributed homogeneously (Fig. 3d). Irinotecan treatment did not significantly reduce the number of Ki-67^+^ cells in spheroids compared to vehicle control.

Since both irinotecan and its active metabolite SN-38 stabilise topoisomerase I–DNA complexes and induce DNA damage, we assessed cell cycle progression as an indicator of irinotecan activity in three-layer spheroids using the fluorescent, ubiquitination-based cell cycle indicator Fucci(CA)5 system^31,32^. Spheroids composed of Fucci-engineered HeLa cells revealed an increase in h2-3-hGem expression when treated with 50 μM irinotecan (Fig. 3e), which was quantified by flow cytometry, indicative of cell cycle arrest either prior to completion of S phase or at the G2 checkpoint (Fig. 3f). Here we used Fucci, rather than gene expression profiles, to visualise changes that occurred and confirm the spatial arrangement of cell cycle arrest within spheroids. Further stratification of irinotecan-treated cells based on SN-38 fluorescence intensity (Fig. 3g) revealed that the global increase in S-phase population was even more pronounced in SN-38^high^ cells compared to SN-38^low^ cells, confirming that the extent of cell cycle arrest correlated with effective drug concentration (Fig. 3h, i).

### scRNA-seq resolves heterogeneity within spheroid layers

**Fig. 4:**
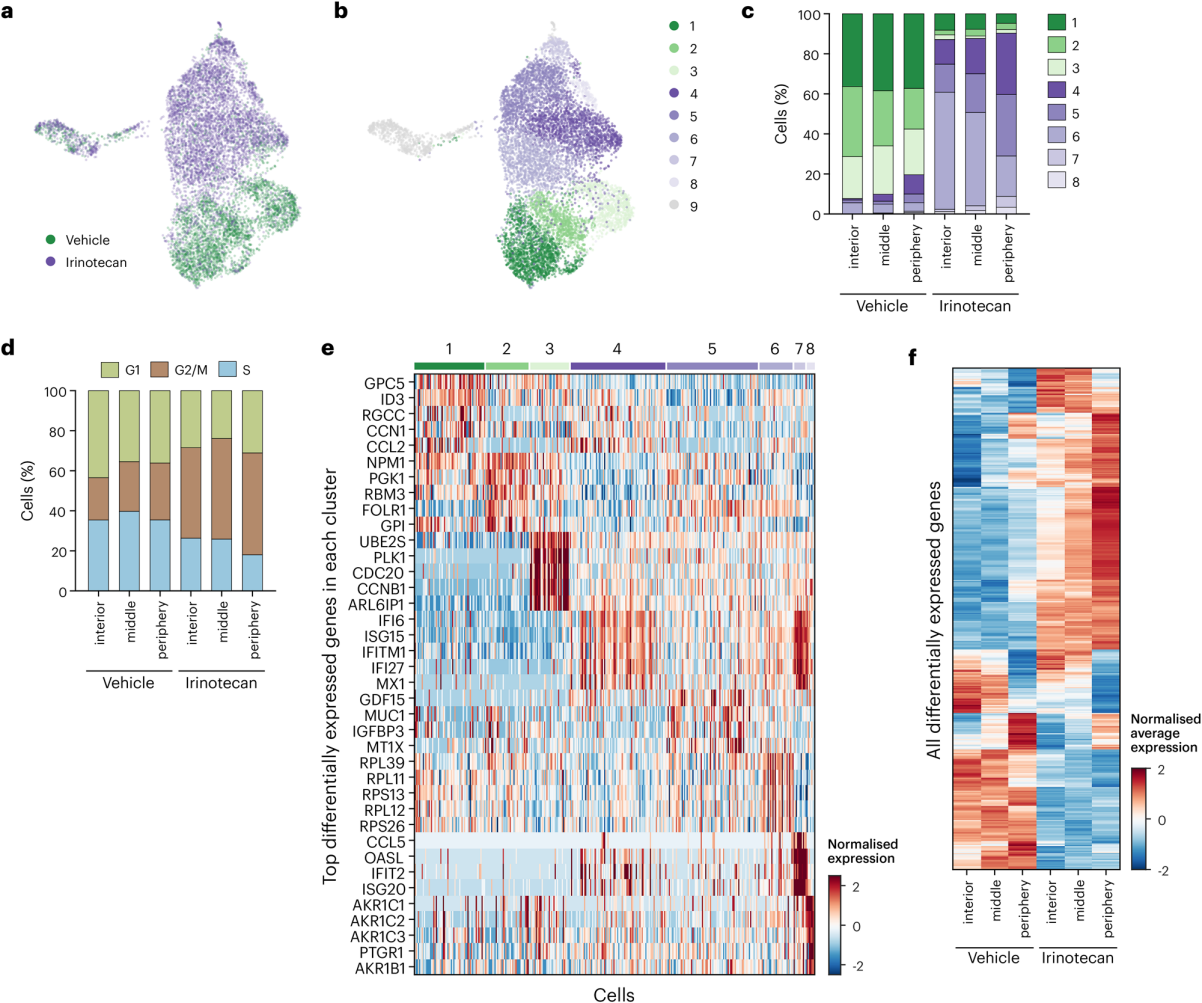
Irinotecan alters transcriptional profiles in multi-layer spheroids. **a** Uniform Manifold Approximation and Projection (UMAP) visualisation of single cells captured and sequenced. Cells are annotated according to whether they were treated with 0.1% v/v DMSO (‘vehicle’) or 50 μM irinotecan for 24 h. **b** UMAP visualisation of single cells, annotated by cell cluster. **c** Distribution of cell clusters according to treatment with vehicle or irinotecan and layer. **d** Irinotecan increases expression of G2/M gene markers. Percentage of cells in each cell cycle from the vehicle control and irinotecan-treated samples. **e** Heatmap enrichment of the top 5 differentially expressed genes (DEGs, |log_2_ fold change|> 0.25 and *p*_adj_ < 0.05) in each cell cluster. **f** Normalised expression levels of DEGs (|log_2_ fold change|> 0.25 and *p*_adj_ < 0.05) between the vehicle and irinotecan-treated spheroids, and each layer. In **e**, **f**, statistical significance for differential gene expression (*p*_adj_) was reported by Seurat using a non-parametric Wilcoxon rank sum test with Bonferroni correction. Source data are provided as a Source Data file.

We sequenced HeLa spheroids composed of three uniquely barcoded layers to study the spatial differences in gene expression triggered by irinotecan treatment. Vehicle control and spheroids treated with 50 μM irinotecan for 24 h were dissociated and libraries were prepared for scRNA-seq. After quality control, 11,921 single cells were assigned an SBO using the D-score deconvolution method; 3748 and 5674 cells were assigned to the vehicle control and the irinotecan-treated spheroids, respectively. Cells were clustered and a clear separation between the vehicle control and drug-treated cells was observed, consistent with broad changes in gene expression induced by irinotecan treatment (Fig. 4a). A variety of relevant reactome pathways were significantly upregulated in irinotecan-treated spheroids (Supplementary Fig. 9).

UMAP clustering revealed similar control populations as in the untreated spheroids, and an additional five clusters that were related to treatment with irinotecan (Fig. 4b). Within the three barcoded layers of these spheroids, we can identify different cell populations based on UMAP clustering of gene expression profiles, suggesting that in future work using co-cultures or more complex cell models, different cell types or subpopulations could also be readily identified within spatially barcoded regions. One subpopulation in the dataset (cluster 9) was enriched in markers of cell death in the same way as for the two- and three-layer control spheroids and was again excluded. In the vehicle control, clusters were evenly distributed across the different layers, whereas the layers of the irinotecan-treated spheroids displayed greater differences in cell clustering (Fig. 4c). As an example, cluster 6 contained more cells from the interior region of the spheroids, while the remaining clusters were enriched in cells from the periphery. We analysed the different layers for markers of cell cycle phase and found a general increase in G2/M markers in irinotecan-treated spheroids consistent with arrest at the G2 checkpoint (Fig. 4d). This agrees with the results in Fig. 3e-i in which cells are prevented from initiating mitosis.

We identified 3970 DEGs between the eight clusters (|log_2_ fold change|> 0.25, *p*_adj_ < 0.05). Single-cell expression across the top 5 DEGs for each cluster is shown in Fig. 4e. Interestingly, cluster 8 displayed an upregulation in aldo/keto reductase (AKR) genes; within this family of stress-related genes, upregulation of *AKR1C3* in particular has been previously identified as a biomarker for irinotecan resistance in colon cancer cells^33^. Visualisation of the average gene expression across spheroid layers for all DEGs highlighted a variety of patterns, including an array of genes that were significantly upregulated compared to vehicle control in a spatially-dependent manner across spheroid layers following treatment with irinotecan (Fig. 4f).

### scTECH-seq enables spatial analysis of irinotecan-induced change

**Fig. 5:**
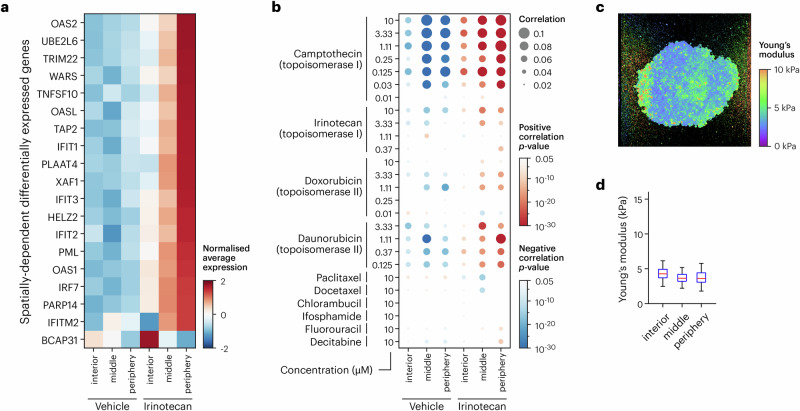
Irinotecan induces spatially dependent gene expression changes in spheroids. **a** Normalised average expression levels of differentially expressed genes (DEGs), filtered for those that display a spatially dependent response to irinotecan while remaining consistent within untreated spheroids. **b** Gene expression profiles for each layer in vehicle and irinotecan-treated spheroids were correlated with 24 h HeLa perturbagen profiles from the Library of Integrated Network-Based Cellular Signatures (LINCS) L1000 database. Dot size represents Kendall’s rank correlation coefficient *τ*, red dots show positive correlation, blue dots negative correlation, and colour scales represent *p*-value. **c** Mechano-microscopy was performed on three-layer spheroids, using fluorophore-tagged short barcode oligonucleotides (SBOs) to identify boundaries between layers; the resulting map of Young’s modulus shows that elasticity is relatively uniform throughout three-layer spheroids. **d** Spheroid elasticity quantified from the image in (**c**) did not significantly vary with depth, suggesting that gene expression differences between layers were not induced by variation in mechanical properties (*n* = 38,566, 97,531, and 189,204 pixels for interior, middle, and peripheral layers, respectively). Box and whisker plots are representative of *n* = 2 spheroids and display median (horizontal line), interquartile range (IQR, box), and 1.5 × IQR (whisker), while values outside 1.5×IQR are not plotted. Source data are provided as a Source Data file.

The unique benefit of our multiplexing model is the ability to classify cells based on spatial location and map drug response across 3D systems. We filtered the average expression data for genes that did not vary in different layers of the control spheroids (to exclude baseline variation due to hypoxia, differences in metabolism, etc.). We identified 197 genes whose expression levels were affected by irinotecan but did not significantly vary between layers (Supplementary Fig. 10), as well as 39 genes that presented a spatially dependent response to irinotecan (Fig. 5a). Within the set of spatially-dependent genes, *PARP14* has been associated with poor prognosis in pancreatic cancer^34^, *IFITM2* has been identified as a marker of gastric cancer growth and metastasis^35^, and *BCAP31* has been identified as a predictor of inferior survival in non-small cell lung cancer^36^. Further validation of the above genes could be undertaken in more physiologically applicable models of the relevant cancers. However, we note that in situ hybridisation and immunofluorescence may not clearly reveal differences between layers due to relatively high cell-to-cell variation in gene expression.

We correlated gene expression profiles from each layer of the vehicle and irinotecan-treated spheroids with gene expression profiles from the Library of Integrated Network-Based Cellular Signatures (LINCS) L1000 chemical response atlas (Fig. 5b)^37^. LINCS datasets were all acquired in HeLa cells following 24 h drug treatment to match spheroid treatment conditions. Gene expression levels in spheroid layers were compared to L1000 expression signatures through Kendall’s rank correlation coefficient *τ*. We observed that the peripheral layer displayed the strongest positive correlations, which decreased in magnitude moving toward the interior, supporting the idea that drug activity was greatest within cells at the spheroid periphery, and that peripheral cells in our 3D culture most closely reflect the conditions under which cells are grown in 2D cultures. Interestingly, stronger positive correlations were observed with camptothecin than irinotecan, suggesting a different transcriptional response in 3D spheroids compared to 2D monolayer cultures, perhaps through differences in crowding, metabolic activity, senescence, and hypoxia. The active metabolite of irinotecan, SN-38, is a structurally related analogue of camptothecin (7-ethyl-10-hydroxycamptothecin), so the gene expression correlation between camptothecin in 2D and irinotecan in 3D could be expected. We also compared the expression profiles from our spheroids to other drugs; expecting to see some positive correlation with topoisomerase II inhibitors, we profiled against doxorubicin and daunorubicin, where we indeed found weaker positive correlations (Fig. 5b). As controls, we profiled against the taxanes paclitaxel and docetaxel, alkylating agents chlorambucil and ifosphamide, and antimetabolites fluorouracil and decitabine, all of which have different modes of action, and accordingly we found minimal similarity in gene expression profiles. Our results are consistent with the hypothesis that spatially encoded architectures can profile differential transcriptional responses to help explain evolving drug resistance and variation in cell response to drugs in a 3D context. With this proof-of-principle in hand, it would be invaluable to conduct more comprehensive studies with more drugs and drug classes, as well as spheroids of different cell composition and size, to further validate and compare gene expression profiles observed in 2D and 3D cultures, with a view toward establishing 3D spatiotemporal drug atlases.

Finally, we examined the mechanical properties of our spheroids using mechano-microscopy, a high-resolution variant of quantitative microelastography. We hypothesised that there may have been a discontinuous structure at spheroid layer boundaries because of stepwise growth, which could lead to variation in drug penetrance. Spheroids were embedded in hydrogels, and the deformation in response to an applied force was measured to obtain elasticity maps. Capitalising on the fluorophore-labelled SBOs present within each of the spheroid layers, we were able to assign elasticity measurements to each of the three regions. The multi-layered spheroids did not exhibit any signs of a disjoint boundary or change in elasticity between the layers (Fig. 5c, d). Thus, the spheroids were relatively homogeneous in structure, and variation in the response to irinotecan was likely due to differences in drug penetration or cellular metabolic activity and not microenvironment. Interestingly, in comparison to our layer-by-layer assemblies, spheroids of a similar size grown in a single step displayed an elasticity that varied inversely with the cellular depth (Supplementary Fig. 10). We profiled a comprehensive set of mechanosensory receptors, adhesion molecules, and fibrotic markers within control spheroids (Supplementary Table 12), but except for *ACTG1*, which showed significantly higher expression in the spheroid interior compared to the periphery (*p*_adj_ ≤ 0.05), no other genes exhibited spatial variation. This finding suggests minimal mechanosensory or adhesion-related heterogeneity in HeLa spheroids, consistent with the uniform elasticity observed by mechano-microscopy.

## Discussion

In this work, we successfully used fluorophore-conjugated SBOs for intracellular barcoding in scRNA-seq experiments, enabling visualisation of barcoded cells. We anticipate that SBOs modified in this manner may also enable cell sorting based on SBO identity, for example, to clean up populations for cell types with non-uniform SBO uptake efficiency, or prior to sequencing to remove cells containing non-unique SBOs. We envision that assembly approaches could be extended beyond our layer-by-layer approach to automated 3D bioprinting to increase throughput and reproducibility. Polymer transfection by scTECH-seq is advantageous because it results in complete cellular internalisation of SBOs via endocytosis. Alternative strategies such as CITE-seq employ lipid- or antibody-based anchoring of SBOs to the cell membrane, which is reversible and prone to barcode exchange between cells. scTECH-seq offers a number of advantages over existing sequencing methods, including compatibility with 5′-multiplex gene expression profiling, which offers better exonic read capture and long-read sequencing potential, and highly uniform barcode distribution that improves demultiplexing efficiency. Furthermore, the departure from chip-based 3′ mRNA capture allows us to multiplex samples to reduce cost, also facilitating experiments on a larger scale or with greater throughput in the future. While existing spatial transcriptomic platforms can achieve impressive resolution in planar tissues, scTECH-seq is uniquely suited for investigating transcriptional dynamics in complex, multilayered 3D cell culture models.

Our antigen-independent barcoding method (scTECH-seq) is sufficient for transfection, cell harvesting, assembly, and treatment of novel layered 3D HeLa architectures over 72–96 h. The labelled cell populations could be resolved by flow cytometry (Supplementary Fig. 3), and D-score demultiplexing assigned over 80% of cells unambiguously in three-layer spheroids, suggesting that intracellular delivery of SBOs by scTECH-seq results in minimal barcode diffusion between layers that is often observed using current lipid- or antibody-tagging technologies. We found evidence of reduced SBO numbers that would likely compromise successful demultiplexing after this time point, as revealed by SBO quantification over time (Supplementary Fig. 2). The fact that SBO abundance correlates with the cell population doubling time suggests that division is the primary determinant of SBO longevity rather than degradation. Since the number of demultiplexed cells correlated with the median number of SBO counts per cell (Supplementary Table 11), we concluded that some barcode loss or degradation had taken place over the longer time taken to grow three-layer spheroids, reducing demultiplexing efficiency in the interior and middle layers. However, we cannot exclude the possibility that cell death or stress in these layers may have also been a factor. Since polymer-based delivery of SBOs is known to result in endolysosomal entrapment, alternative delivery reagents targeting different uptake routes^38^, modification of SBOs with phosphorothioate linkages to reduce nuclease-mediated degradation^39^, or the generation of cell lines with genetically encoded barcode sequences and/or fluorescent reporters^40^ could help preserve SBO integrity in future work. Furthermore, additional layers, greater numbers or multiplexing of fluorescent tags, or more rapid addition of layers to the growing spheroid could facilitate a more fine-grained analysis of spatial gene expression. It would be prudent to confirm cell viability when extending the culture to a greater number of layers or over a longer time period.

While spatial transcriptomics platforms are ideal for exploring gene expression over comparatively large tissue sections, spatial transcriptomics for spheroids has historically been constrained by the resolution of capture spots. Recent advances indicate that while spatial transcriptomics can be applied at the spheroid scale, resolving internal expression patterns in 3D cultures to a meaningful degree, iterative optimisation may be required and sample preparation remains technically challenging^16^. Alternatively, a previously reported technique (SEEP-seq) for spatial transcriptomics in 3D cultures makes use of dye penetration to indirectly quantify cell depth in 3D cultures prior to single-cell analysis^41^. SEEP-seq provides information in relation to drug diffusivity, whereas our approach is more application-agnostic. We see complementary potential for SEEP-seq and our technique, which is not limited to diffusion, and could be used to profile aspects of 3D culture, including proliferation or epithelial–mesenchymal transition, which may not be directly related to diffusivity, especially in more complex architectures. An alternative to physical spatial transcriptomics platforms is computational statistical reconstruction methods that infer spatial position from droplet-based scRNA-seq data, such as novoSpaRc^42^, SpaOTsc^43^, and Tangram^44^. These approaches rely on matching gene expression profiles to a spatial reference atlas, or to each other, to reconstruct a pseudo-spatial map. While these techniques are powerful when high-quality spatial reference data are available, their accuracy can be limited in novel systems, cultures consisting of a single cell type, or engineered cultures lacking a priori spatial maps, as is the case here. In contrast, scTECH-seq provides a direct physical encoding of spatial origin, enabling spatially resolved single-cell transcriptomics in de novo 3D architectures without reliance on external spatial references. There is scope to incorporate statistical reconstruction methods in the future, provided that the spatial fidelity of such techniques can be experimentally confirmed.

As we have demonstrated, SBOs not only facilitate 5′-multiplex scRNA-seq, but can also encode the spatial arrangement of cells, allowing scRNA-seq data to be related to cell location within engineered 3D cell culture architectures. Our data support the hypothesis that spheroid cultures display limited cell growth and proliferation within the core due to dense cell packing and a limited supply of nutrients and oxygen. Exploration of cellular response to irinotecan in the 3D context revealed a gradient in the transcriptomic response that indicated the greatest drug-related effects at the periphery. Extending the fluorescent barcoding idea further, the visualisation of SBOs within spheroids also supported in situ correlative imaging techniques; we demonstrated this possibility using mechano-microscopy to construct maps of elasticity (i.e., Young’s modulus) in 3D cultures. The ability to relate scRNA-seq sequencing data to matrix elasticity could facilitate correlative studies of gene expression in the context of mechanobiology.

While we validated spatially-resolved scTECH-seq here using HeLa spheroids, future work could explore applications in other immortalised cell types, primary cells, co-culture systems, or organotypic models such as hepatic or neural spheroids to extend the utility of scTECH-seq in drug screening, toxicity testing, and developmental biology. We have previously applied scTECH-seq to human mammary cells to study epithelial-to-mesenchymal transition in 2D cultures, which shows the applicability of our method to other cell types and cellular transitions, highlighting the versatility of this technology. In future applications involving stem cell differentiation or long-term culture, additional factors such as cellular migration, barcode stability, and dynamic gene expression changes would need to be considered; in such cases, stable genetic barcoding or chemically modified SBOs may be more suitable. However, slower-dividing cells, or studies involving terminal differentiation in which cells stop dividing, may support longer-term spatial barcoding in spheroids or developing organoids worthy of further exploration. While over long-term studies, migration of cells within a spheroid could result in mixing of spatially tagged cell populations, our use of fluorescent-tagged SBOs would allow any such migration of barcoded cell subpopulations to be visualised, while also supporting single-cell transcriptomic analysis. Thus, migration and differentiation could potentially be monitored simultaneously in another intriguing avenue for further study that is otherwise not possible with existing technologies. Future work could expand the drug panel to include compounds with diverse mechanisms of action to further demonstrate the utility of scTECH-seq in profiling spatially heterogeneous drug responses. We envisage that further correlative analysis between 2D and 3D drug response will be helpful in establishing expected gene expression profiles in 3D and explaining heterogeneous activity and outcomes in response to drug treatment. Additionally, future studies could aim to quantify drug-induced TOP1 cleavage complexes within spheroid layers to directly link spatial transcriptomic profiles with the primary mechanism of action of TOP1 inhibitors, providing a more comprehensive understanding of spatial drug responses.

We conclude that transfection-based hashing methods such as scTECH-seq are compatible with the stepwise growth of layered HeLa spheroids. Fluorophore modification of SBOs enables cell subpopulations within the assembly to be imaged, and the use of unique SBO sequences facilitates 5′-multiplexing of scRNA-seq data to collect spatially resolved, transcriptomic information. Our spatially resolved data highlights differences in gene expression that could form the basis for 3D gene expression atlases of drug response. Using scTECH-seq, the construction of engineered cellular architectures adds spatial information to studies of gene expression using in vitro 3D culture models.

## Methods

Experimental protocols complied with relevant ethical regulations, including the requirements of the Office of the Gene Technology Regulator (OGTR) and Department of Agriculture, Fisheries and Forestry (DAFF) in Australia, and were approved by the University of Western Australia’s Institutional Biosafety Committee.

### SBO barcode design

The 10X Genomics 5′ feature short barcode oligonucleotides (SBOs) were ordered from Integrated DNA Technologies (IDT Asia Pacific). SBOs (69 nt) were designed according to 10X Genomics specifications, except with the following modifications: oligonucleotides were modified by replacing the C12 amine linker with a shorter chain, internal modified dC (/iAmC6C/) and adding a fluorophore at the 5′ end (/5Cy5/ or /5Cy3/). Oligonucleotides were ordered at a 250 nmol scale with HPLC purification. A representative SBO sequence using feature barcode 13 (in lowercase) as an example is 5′-/5Cy3//iAmMC6C/GGAGATGTGTATAAGAGACAGNNNNNNNNNNataatcattacgtggNNNNNNNNNCCCATATAAGA*A*A-3′ (* = phosphorothioate). Feature barcode sequences used in this study are presented in Supplementary Table 1.

### Cell culture

HeLa cells (ATCC) were cultured in Dulbecco’s Modified Eagle Medium (DMEM) (Gibco 10569044) supplemented with 10% v/v FBS at 37 °C and 5% CO_2_. HeLa cells were maintained at 37 °C in a humidified incubator with 5% CO_2_ and passaged at 70–80% confluency using 1× TrypLE Express (Gibco).

### Transfection protocol

Dendritic polymer for transfection of SBOs was synthesised as previously described^45,46^. Briefly, the dendritic polymers are assembled from a statistical copolymer backbone consisting of 25 mol% glycidyl methacrylate (GMA) and 75 mol% 2-hydroxyethyl methacrylate (HEMA), whereby the epoxide groups on the GMA monomers enable the attachment of alkyne-root generation 5.0 poly(amido amine) dendrons through an azide–alkyne click reaction. Polymer–SBO complexes were formed at an amine-to-phosphate (N/P) ratio of 30. For SBO labelling, HeLa cells were seeded at 150,000 cells/mL in a 24-well plate and incubated overnight at 37 °C and 5% CO_2_. Polymer (3 mg/mL stock solution in ddH_2_O) and SBO (100 μM stock solution in ddH_2_O) were diluted in Opti-MEM (Gibco 31985070). SBO was diluted to 657 nM for a total of 23 pmol (1.59 nmol DNA phosphate groups) in 35 μL, while the polymer was diluted to 0.326 mg/mL for a total of 47.7 nmol primary amines in 35 μL. Equal volumes of polymer (34 μL) and SBO (34 μL) solutions were mixed gently but thoroughly and incubated for 25 min at room temperature. Cells were washed with 1×PBS once to remove serum and the media was replaced with 150 μL Opti-MEM. 65 μL polymer–SBO complexes were added per well and cells were incubated at 37 °C for 4 h. Transfection efficiency was visualised with an epifluorescence microscope and quantified via flow cytometry.

### Flow cytometry

For 2D cell cultures, the transfection solution was removed and cells were washed with PBS twice. Cells were collected with TrypLE and 2× FACS buffer (2 mM EDTA + 6% FBS in 1× PBS) for flow cytometry. For spheroid dissociation, cells were washed with PBS twice before incubation with TrypLE for 20 min at 37 °C. Spheroids were manually dissociated by pipette mixing 20–30 times until cell clumps were no longer visible. Cells were collected by centrifugation (300 *g*, 5 min) and resuspended in 2× FACS buffer. At least 10,000 single-cell events, gated on side scatter area *vs* height, were recorded for analysis. Untreated or unstained cells were used as a negative control. Data were analysed post-acquisition using FlowJo software v10.10.0.

### Spheroid formation

To form spheroids, transfected HeLa cells were plated in Nunclon Sphera 96-well U-shaped-bottom microplates (Thermo Scientific 174925). Spheroid formation was visualised under phase contrast microscopy. To grow three-layer spheroids, HeLa cells were transfected as described above. Transfected cells were harvested and plated in 96-well low adhesion plates at 2000 cells/well in 100 μL media. To form the second layer, the following day, a new sample of HeLa cells was transfected with a different SBO prior to harvesting and 3000 cells/well were seeded into fresh wells. Spheroids formed the previous day were transferred to the new well, with one spheroid per well. This process was repeated on the third day, using 5000 cells/well to form the final layer. Each day, cells were incubated for 24 h prior to the addition of the next layer. Two-layer spheroids were prepared the same way, using 5000 cells/well for both layers.

### Spheroid dissociation and viability assay

Propidium iodide was used to assess the proportion of live/dead cells in spheroids after irinotecan treatment. 24 h after the third layer of cells was added, spheroids were treated with irinotecan. Irinotecan hydrochloride (Sigma-Aldrich I1406) was dissolved at 50 mg/mL in DMSO. Irinotecan was diluted in media to a 1 mM working stock and serial dilutions were used to make 200 μM, 100 μM, 40 μM, and 10 μM dilutions. Half of the volume in each well was removed and diluted irinotecan stocks were added in equal volume to achieve final concentrations of 100, 50, 20 and 5 μM. Spheroids were incubated for 24 h. Vehicle (0.1% v/v DMSO) and untreated spheroids were used as controls. After 24 h, spheroids (*n* = 8) for each treatment were transferred into a 1.5 mL tube. Media was removed and 300 μL 1× TrypLE Express was added, and spheroids were incubated for 15 min at 37 °C. After 15 min, another 300 μL TrypLE was added to each tube and spheroids were pipetted up and down 20 times using a 1000 μL single-channel pipette until no visible cell clumps remained. Cells were centrifuged at 300 *g* for 5 min and resuspended in 2× FACS buffer supplemented with 4 μg/mL propidium iodide solution. Cells were stored on ice until flow cytometry was performed (BD SORP Fortessa), using a 561 nm laser and 610/20 nm bandpass filter. Data was analysed using FlowJo v10.

### Spheroid sectioning

Following spheroid growth or drug treatment, spheroids were transferred into 1.5 mL tubes. Media was removed and 4% paraformaldehyde (PFA) in 1× PBS was added and spheroids were incubated for 20 min at 25 °C. Spheroids were washed with 1× PBS twice and incubated in 30% sucrose overnight at 4 °C for cryoprotection. The following day, spheroids were frozen in optimal cutting temperature (OCT) compound for cryosectioning using 12 μm slices.

### Ki-67 immunofluorescence

Hydrophobic barrier pens were used to create a water-repellent barrier around spheroid sections. All incubation steps were conducted at 25 °C unless otherwise stated. Spheroid sections were fixed to slides with 4% w/v PFA for 15 min and washed with 1× PBS twice, 5 min each. Antigen retrieval was performed by immersing slides in citrate buffer (10 mM sodium citrate, pH 6.0 + 0.05% v/v Tween 20) overnight at 60 °C. Sections were washed with 1× PBS, permeabilised with 0.1% v/v Triton X-100 in 1× PBS for 10 min, followed by 2 × 5 min washes in 1× PBS. Blocking was conducted using 2% w/v BSA in 1× PBS for 1 h and incubated with anti-Ki67 antibody (Abcam ab16667) in a 1:200 dilution, overnight at 4 °C. Sections were washed 3 × 5 min in 0.1% v/v Tween 20 in 1× PBS. Secondary antibody incubation was left for 1 h using anti-rabbit Alexa Fluor 488-conjugated at a 1:500 dilution. Sections were washed with 0.1% v/v Tween 20 in 1× PBS twice and 1× PBS before mounting glass coverslips with Fluoromount G (Southern Biotech). Epifluorescence images were acquired (Nikon ECLIPSE Ti2) using a Plan Apo λ 10×/0.45 objective and analysed using ImageJ.

### Fucci cells

A stable HeLa-Fucci cell line was generated using the Super PiggyBac Transposase Expression Vector system (System Biosciences, PB210PA-1) together with tFucci(CA)5 plasmid^32^ (Addgene 153521), as per manufacturer’s protocol. Briefly, HeLa cells were seeded at a density of 80,000 cells/mL in a 12-well plate and left to settle overnight (~16 h). For transfection, 200 ng of the PiggyBac transposase was mixed with 500 ng of tFucci(CA)5 plasmid (overall ratio of 1:2.5 transposase: transposon vector), and diluted to a total of 30 μL in Opti-MEM (Gibco 31985070). Separately, 0.7 μL Lipofectamine 2000 (Invitrogen 11668027) was diluted to 30 μL in Opti-MEM, and the two solutions were mixed thoroughly (total of 60 μL transfection cocktail) and incubated for 25 min at 25 °C. Meanwhile, cell media was removed, cells were rinsed twice with 1× PBS, and 400 μL Opti-MEM was added. After incubation, 60 μL of the transfection cocktail was added to the well, cells were incubated at 37 °C for 3 h, after which 540 μL complete media was added and cells were incubated for a further 45 h (48 h total). Cells were detached, transferred to a 10 cm dish, and left overnight to settle. Cells were then routinely cultured in selection media (DMEM + 10% v/v FBS + Blasticidin S HCl 10–30 μg/mL) until 100% of cells expressed the protein markers.

### scRNA-seq sample preparation and sequencing

Three-layer spheroids were formed as described above. Spheroids were treated with 50 μM irinotecan or 0.1% v/v DMSO for 24 h. Spheroids were dissociated as outlined in ‘Spheroid dissociation and viability assay’. Single cells from the vehicle control and irinotecan spheroids were counted and pooled in equal numbers at 2000 cell/μL in 0.4% w/v BSA for sequencing. Cell viability was confirmed to be >90%. Cells were loaded onto a 10x Genomics Chromium Controller, Chip K (1000286) and libraries were prepared using the Chromium Next GEM single cell 5′ kit v2 and 5′ Feature Barcode kit following manufacturer guidelines (10X Genomics 1000263, 1000265). The Illumina iSeq 100 platform was used for initial QC of the libraries and deeper sequencing was conducted on the Illumina NovaSeq 6000 in the vendor-recommended format to reach up to 50,000 reads per cell. Fastq files were generated with Illumina bcl2fastq v2.20.0.422, allowing 1 mismatch in each index. 10X Genomics Cell Ranger was used for transcript alignment and the generation of gene count and feature barcode matrices.

### Single-cell data analysis

The D-score deconvolution algorithm was used to assign SBOs to single cells^21^. Three-layer spheroids are used here as an illustrative example. A total of 11,921 single cells were sequenced and successfully assigned an SBO. Cells were filtered for 200–95,000 total genes and less than 15% mitochondrial genes were detected. 9422 cells were used in downstream analysis. Data were normalised and the top 3000 variable genes were selected for scaling. The scaled data was used for principal component, dimensionality reduction and UMAP clustering. The Wilcoxon rank sum test was used to calculate DEGs (|log_2_ fold change | > 0.25, *p* < 0.05). DEGs were corrected for multiple testing using the adjusted *p*-value. The following software versions were used: Cell Ranger v7.0.0, ClusterProfiler v4.6.2, GSEABase v1.60.0, ReactomePA v1.42.0, Seurat v4.3.0.1.

### Mechano-microscopy

Mechano-microscopy was conducted as previously described^47^. Briefly, the mechano-microscopy system is built upon an optical coherence microscopy (OCM) setup using a supercontinuum laser for broadband light. The Michelson interferometer in the OCM system includes filters to create a spectral range of 650 nm to 950 nm and achieves ~1.4 μm axial resolution and ~0.5 μm lateral resolution. Two B-scans were acquired per location and B-scans were analysed to measure local displacement and strain, allowing for elasticity estimation using the stress–strain response of a pre-characterised compliant layer placed at the bottom of the sample. A confocal fluorescence microscopy system and a photomultiplier tube detector is integrated alongside the OCM to visualise fluorescent barcodes.

### SBO inheritance and longevity analysis

For time-lapse imaging to observe SBO inheritance by daughter cells, HeLa cells were seeded in 4-well chamber slides (Ibidi 80427) and incubated overnight before transfection. All quantities for transfection were scaled from a 24-well plate according to the culture surface area using SBO01-Cy5 and SBO13-Cy3. Cells were incubated with the transfection mix for 2 h before adding complete media and imaging overnight. Epifluorescence and phase contrast images were captured every 10 min for 2 h, every 30 min for the following 6 h, and every 60 min thereafter for Cy3 and Cy5 (Nikon ECLIPSE Ti2, S Plan Fluor ELWD 20×/0.45 objective).

For SBO quantification over time by qPCR, HeLa cells were seeded at 0.3 × 10⁶ cells per well in 6-well plates and incubated overnight before transfection. Two model barcode oligo sequences (SBO05: 5′-/5AmMC12/CGGAGATGTGTATAAGAGACAGCAGGNNNNNNTGGTGACAAGTATCTGAACCACGACCCATATAAGA*A*A-3′, SBO08: 5′-/5AmMC12/CGGAGATGTGTATAAGAGACAGCAGGNNNNNNGCCAAGATCAGGTCCCAAAGGCAGCCCATATAAGA*A*A-3′; * represents phosphorothioate linkage) were ordered from Integrated DNA Technologies at a 250 nmol scale with HPLC purification (NB: these oligos were modified slightly to facilitate PCR primer design and are therefore not suitable for sequencing experiments). Equal amounts of SBO05 and SBO08 were mixed and diluted to a final concentration of 6.57 nM (i.e., 3.29 nM each) in Opti-MEM (Gibco) and complexed with polymer at an N/P ratio of 30. Equal volumes of SBO and polymer were mixed and left to incubate at room temperature for 25 min to form transfection complexes. After rinsing the cells with PBS, 350 μL of the complexes were added to each well. Cells were incubated with the complexes at 37 °C for 4 h, after which 2 mL of complete medium was added without removing the transfection mixture. Samples were collected at 24, 72, 96, and 120 h post-transfection. All cells were washed once with PBS, pelleted, and stored at −80 °C until further use. Genomic DNA was extracted using the ISOLATE II Genomic DNA Kit (Bioline BIO-52067) following the manufacturer’s protocol, with lysis carried out at 65 °C for 1 h. DNA was eluted twice in 50 μL of pre-warmed elution buffer. Concentration and purity were measured using NanoDrop Eight (Thermo Fisher Scientific). qPCR was performed using the QuantiNova SYBR Green PCR Kit (Qiagen 208056) on an AriaMx Real-Time PCR System (Agilent Technologies) in a 96-well plate format. Primers were ordered from Integrated DNA Technologies at a 25 nmol scale with standard desalting (SBO forward: 5′-GGAGATGTGTATAAGAGACAGCAGG-3′, SBO05 reverse: 5′-GTCGTGGTTCAGATACTTGTCACC-3′, SBO08 reverse: 5′-CTGCCTTTGGGACCTGATCTTG-3′, *AGTR1* forward: 5′-GGTTTCCACTGCCAAAACAATGC-3′, *AGTR1* reverse: 5′-CTAGGAAGACACTACTACTTGGGACC-3′). Reactions were set up in technical triplicates, amplifying the SBO sequence of interest, with *AGTR1* used as the internal reference gene for normalisation. The cycling protocol included an initial denaturation at 95 °C for 3 min, followed by 40 cycles of 95 °C for 15 s, 58 °C for 30 s, and 72 °C for 30 s. Melt curve analysis was performed after amplification to confirm primer specificity. Relative SBO levels were calculated using the comparative Ct (ΔΔCt) method. The fold change at each time point was calculated as 2^-ΔΔCt^ relative to the average ΔCt at the 24-hour time point, and error propagation was based on the standard deviation of ΔΔCt values from technical replicates.

### Statistics and reproducibility

No statistical method was used to predetermine sample size. Due to cost, one sequencing replicate was performed for each sample. Unless specified otherwise (e.g., cluster 4 in Fig. 2b and cluster 9 in Fig. 4b), data were not excluded from downstream analyses. Samples in 2D cell culture and 3D spheroids were assigned at random to control or treatment groups. Investigators were not blinded to allocation during experiments and outcome assessment, except that initial processing of single-cell data (CellRanger and D-score demultiplexing) was blinded as to which SBO feature barcode sequences corresponded to which experimental samples. Further analyses of sequencing results were not blinded.

### Reporting summary

Further information on research design is available in the Nature Portfolio Reporting Summary linked to this article.

## Supplementary information

Supplementary information

Reporting Summary

Transparent Peer Review file

## Source data

Source data

## Data Availability

The scRNA-seq datasets generated in this study have been deposited in the National Centre for Biotechnology Information (NCBI) Gene Expression Omnibus (GEO) under accession number GSE245416. Processed data used in the figures are provided with this paper as a Source Data file. Raw data have been deposited at 10.5281/zenodo.20373611. All other data is available upon request. Source data are provided with this paper.

## Code availability

The R code used for data processing is available at 10.5281/zenodo.20373611. D-score software is available at 10.5281/zenodo.20375079.
