# Supplementary material for: Single-cell RNA sequencing profiles drug activity within spatially engineered 3D cultures
Source: Nat Commun. 2026 Jun 19;17:7735. doi: 10.1038/s41467-026-74587-8 (PMC13434128; doi:10.1038/s41467-026-74587-8)
Supplement: Supplementary file 1 [file 41467_2026_74587_MOESM1_ESM.pdf]

Supplementary Information

Single-cell RNA sequencing profiles drug activity within spatially engineered 3D cultures

Jessica J. King[1], Alireza Mowla[2,3], Jessica A. Kretzmann[1,4], Nishka Bhalla[1,4], Giselle Sugianto[1], Marck Norret[1], Ulrich D. Kadolsky[5], Munir Iqbal[5], Alka Saxena[5], Sebastian E. Amos[6], Yu Suk Choi[6], Brendan F. Kennedy[2,3,7], K. Swaminathan Iyer[1,4], Nicole M. Smith[1,4]*, and Cameron W. Evans[1,4,8]*

[1] School of Molecular Sciences, The University of Western Australia, Perth WA 6009, Australia.

[2] BRITElab, Harry Perkins Institute of Medical Research, QEII Medical Centre and Centre for Medical Research, The University of Western Australia, Nedlands, Perth WA 6009, Australia.

[3] Department of Electrical, Electronic & Computer Engineering, School of Engineering, The University of Western Australia, Perth, WA 6009, Australia.

[4] ARC Training Centre for Next-Generation Biomedical Analysis, The University of Western Australia, Perth WA 6009, Australia.

[5] Genomics WA, Harry Perkins Institute of Medical Research, The Kids Research Institute Australia, and The University of Western Australia, Nedlands, Perth WA 6009, Australia.

[6] School of Human Sciences, University of Western Australia, Perth WA 6009, Australia.

[7] Institute of Physics, Faculty of Physics, Astronomy and Informatics, Nicolaus Copernicus University in Toruń, 87-100 Toruń, Poland.

[8] School of Pharmacy and Pharmacology, University of Tasmania, Sandy Bay TAS 7005, Australia.

* Corresponding author: Cameron W. Evans, cameron.evans@uwa.edu.au; Nicole M. Smith, nicole.smith@uwa.edu.au.

List of Supplementary Figures

Supplementary Figure 1. Short barcode oligo (SBO) design S4

Supplementary Figure 2. SBOs are inherited by daughter cells following division S4

Supplementary Figure 3. Cyanine fluorophore-tagged SBOs can be used for sorting cells S5

Supplementary Figure 4. UMAP in two-layer untreated spheroids S6

Supplementary Figure 5. Cell cycle in two-layer spheroids S6

Supplementary Figure 6. Pathway analysis in two-layer spheroids S7

Supplementary Figure 7. Gene ontology (biological processes) S8

Supplementary Figure 8. Flow cytometry assessment of viability in spheroids S9

Supplementary Figure 9. Normalised enrichment scores for Reactome pathways S10

Supplementary Figure 10. Normalised average expression levels of non-spatial DEGs S11

Supplementary Figure 11. Layered spheroids display uniform stiffness S12

List of Supplementary Tables

Supplementary Table 1. SBO sequences used in this study S13

Supplementary Table 2. Raw transcript counts comparing fluorophore conjugation to SBOs S13

Supplementary Table 3. Raw gene counts comparing fluorophore conjugation to SBOs S13

Supplementary Table 4. Raw SBO counts comparing fluorophore conjugation to SBOs S13

Supplementary Table 5. Raw transcript counts in two-layer spheroids S14

Supplementary Table 6. Raw gene counts in two-layer spheroids S14

Supplementary Table 7. Raw SBO counts in two-layer spheroids S14

Supplementary Table 8. DEGs in two-layer spheroids. S14

Supplementary Table 9. Raw transcript counts in three-layer spheroids S15

Supplementary Table 10. Raw gene counts in three-layer spheroids S15

Supplementary Table 11. Raw SBO counts in three-layer spheroids S15

Supplementary Table 12. DEGs for mechanosensory, adhesion, fibrotic markers S16

Supplementary Notes

Analysis of two-layer spheroid single-cell RNA sequencing data

We analysed the single-cell RNA-seq data obtained from two-layer spheroids. Unsupervised uniform manifold approximation and projection (UMAP) clustering revealed three subpopulations based on gene expression that were uniformly distributed between the two layers (Supplementary Fig. S4). Gene expression differences in two of these clusters could be attributed to cell cycle progression (Supplementary Fig. S4), while the third and smallest subpopulation displayed substantially different expression and strong downregulation of housekeeping and other genes, indicative of potential loss of homeostasis and cell death. We speculate that this minor subpopulation of cells arose during dissociation of spheroids, since cells from both layers of the spheroid were equally represented. Between the two different layers of the spheroids, only 16 genes were differentially expressed ($|\log_2 FC| > 0.25$, $p_{adj} < 0.05$, Supplementary Table 8). Pathway analysis based on this small set of genes highlighted changes in glucose synthesis and metabolism, CO_2 accumulation and hypoxia, and some p53-mediated pathways (Supplementary Fig. S5).

Supplementary Figures

5′-/5Cy3//iAmMC6C/GGAGATGTGTATAAGAGACAGNNNNNNNNNNataatcattacgtggNNNNNNNNNCCCATATAAGA*A*A-3′

fluorophore | Nextera partial read 2 | 10 nt | Feature barcode | 9 nt | Capture sequence

Supplementary Figure 1. Short barcode oligo (SBO) design, incorporating fluorophore at the 5′ end.

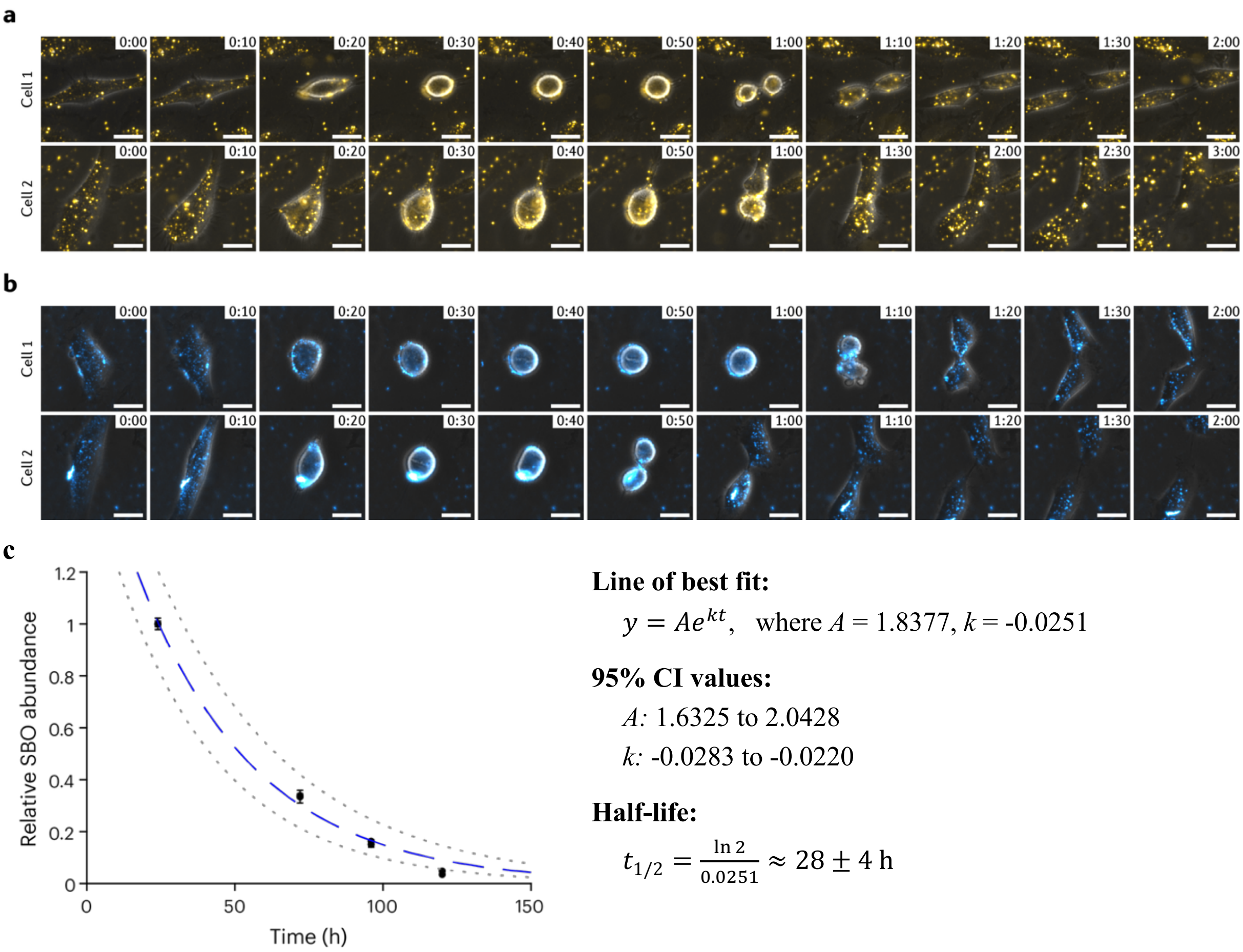

Supplementary Figure 2. SBOs are inherited by daughter cells following division. Two regions of interest (dividing cells) are shown for each of two fluorescent-tagged SBOs. **(a)** Epifluorescence microscopy shows SBOs present before and after cell division, with SBO13-Cy3 shown in yellow, **(b)** SBO01-Cy5 shown in cyan. Times indicated are times elapsed since the first frame shown in the montage in h:mm format. Scale bars 20 µm. **(c)** DNA was extracted from cell samples at 24, 72, 96, and 120 h post-transfection and two different SBOs were quantified by qPCR relative to *AGTR1* gene. SBO abundance was plotted normalised to 24 h timepoint. Black: data points for SBO05 and SBO08, blue dashed line: line of best fit to exponential function, grey dotted lines: 95% CI. From the equation of best fit, the apparent half-life of SBOs in HeLa cells is 28 ± 4 h. qPCR results were determined from n = 3 technical replicates per sample, and n = 2 different barcodes (SBO05, SBO08) transfected in the same cells (note results from each of the two SBOs show a high level of agreement and overlap on the plot).

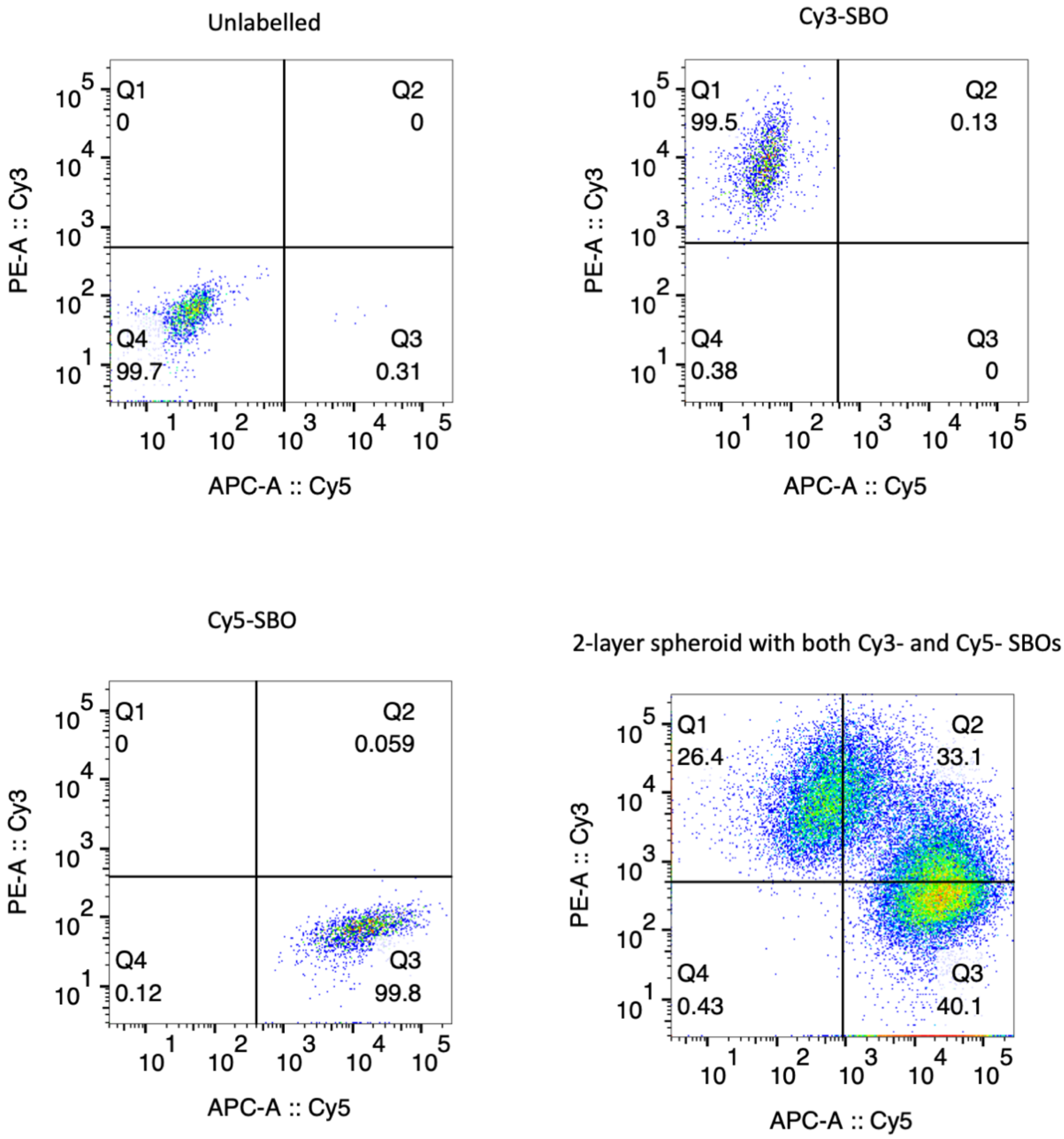

Supplementary Figure 3. Cyanine fluorophore-tagged SBOs can be used for sorting cells with a single SBO. Dissociated single cells from two-layer spheroids containing Cy5-SBO and Cy3-SBO labelled cells maintain their SBOs and are still resolved as distinct populations. 80,000 single cells were collected. Representative of *n* = 2 independent replicates.

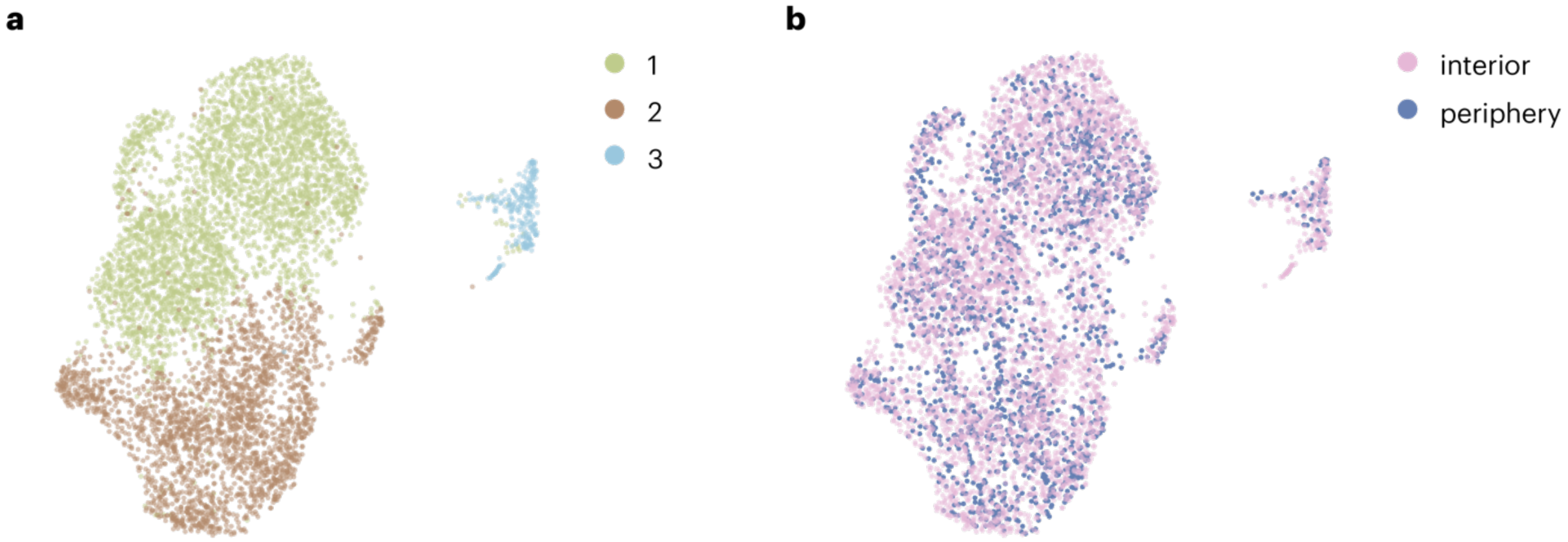

Supplementary Figure 4. UMAP in two-layer untreated spheroids. **(a)** Cells are clustered into three subpopulations. **(b)** Cells mapped in the same UMAP plot according to cell location within spheroids shows that gene expression patterns are not spatially dependent since cells from the interior and periphery are found uniformly across all clusters.

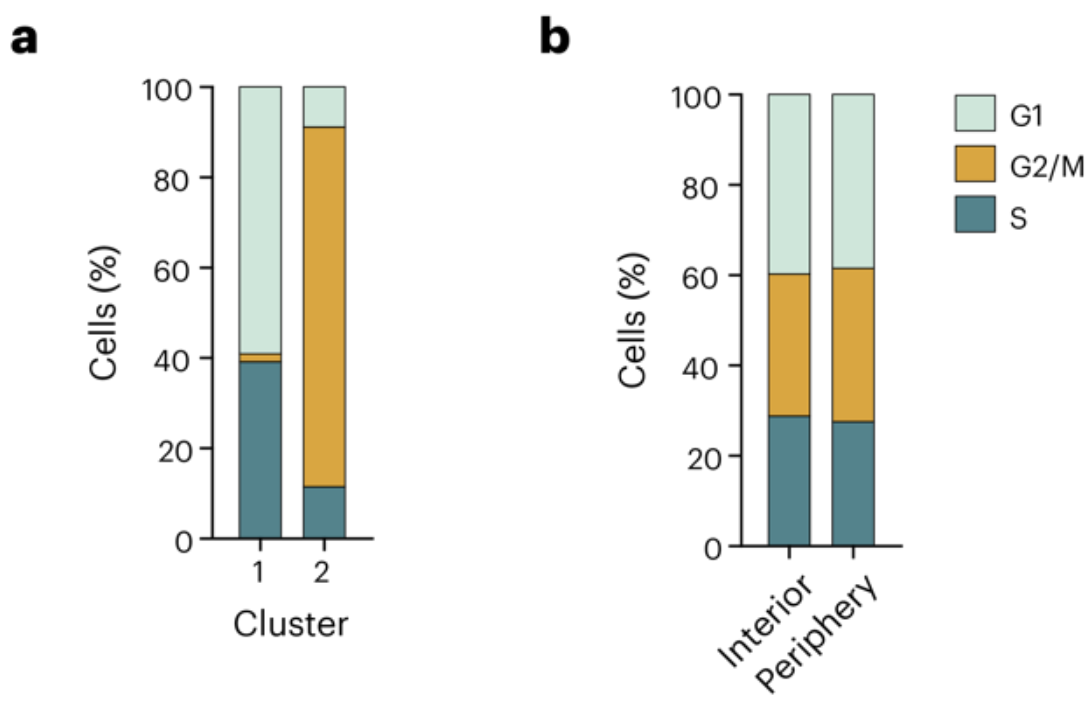

Supplementary Figure 5. Cell cycle in two-layer spheroids. **(a)** Cell cycle evaluated by cluster shows the principal differences between the two major clusters is due to mitosis. **(b)** Cell cycle evaluated according to spheroid layer shows that cell cycle is evenly distributed throughout the whole spheroid. These plots are consistent with observations in Supplementary Figure 2.

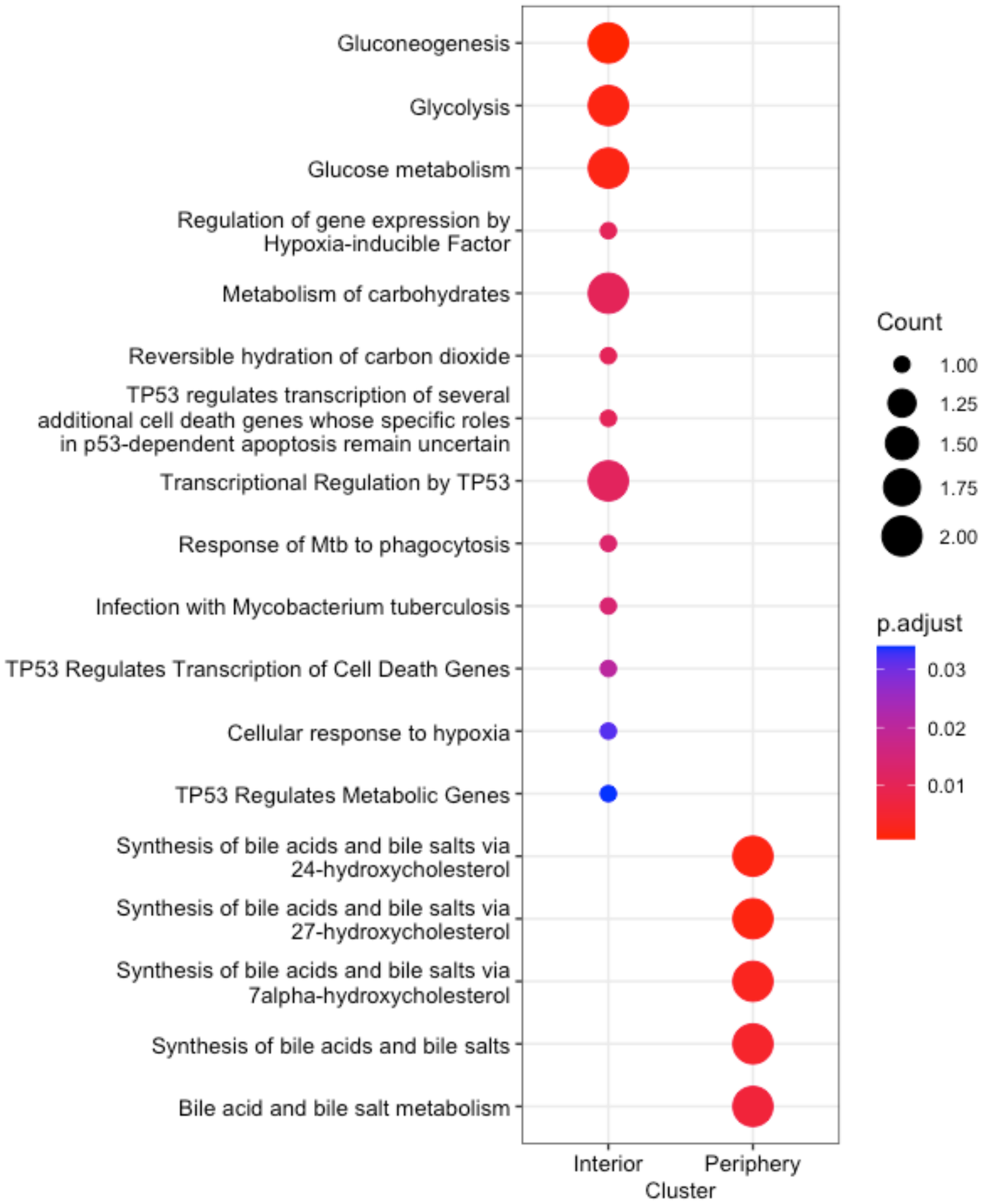

Supplementary Figure 6. Pathway analysis in two-layer spheroids.

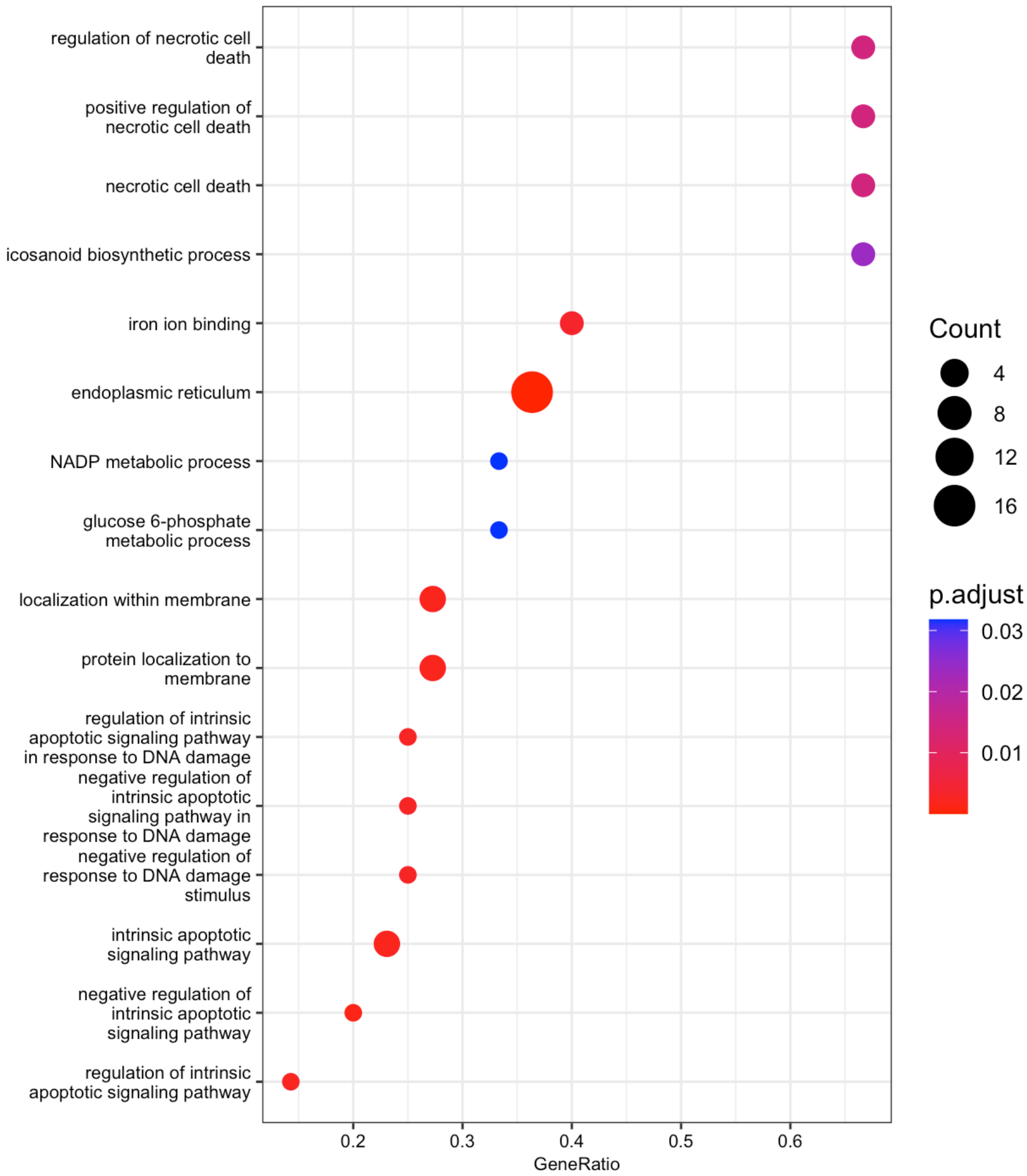

Supplementary Figure 7. Gene ontology (biological processes) enrichment demonstrate that the cells within spheroid cores are enriched for apoptotic and necrotic pathways. Dot size indicates gene counts expressed in each pathway and colour indicates adjusted *p*-value.

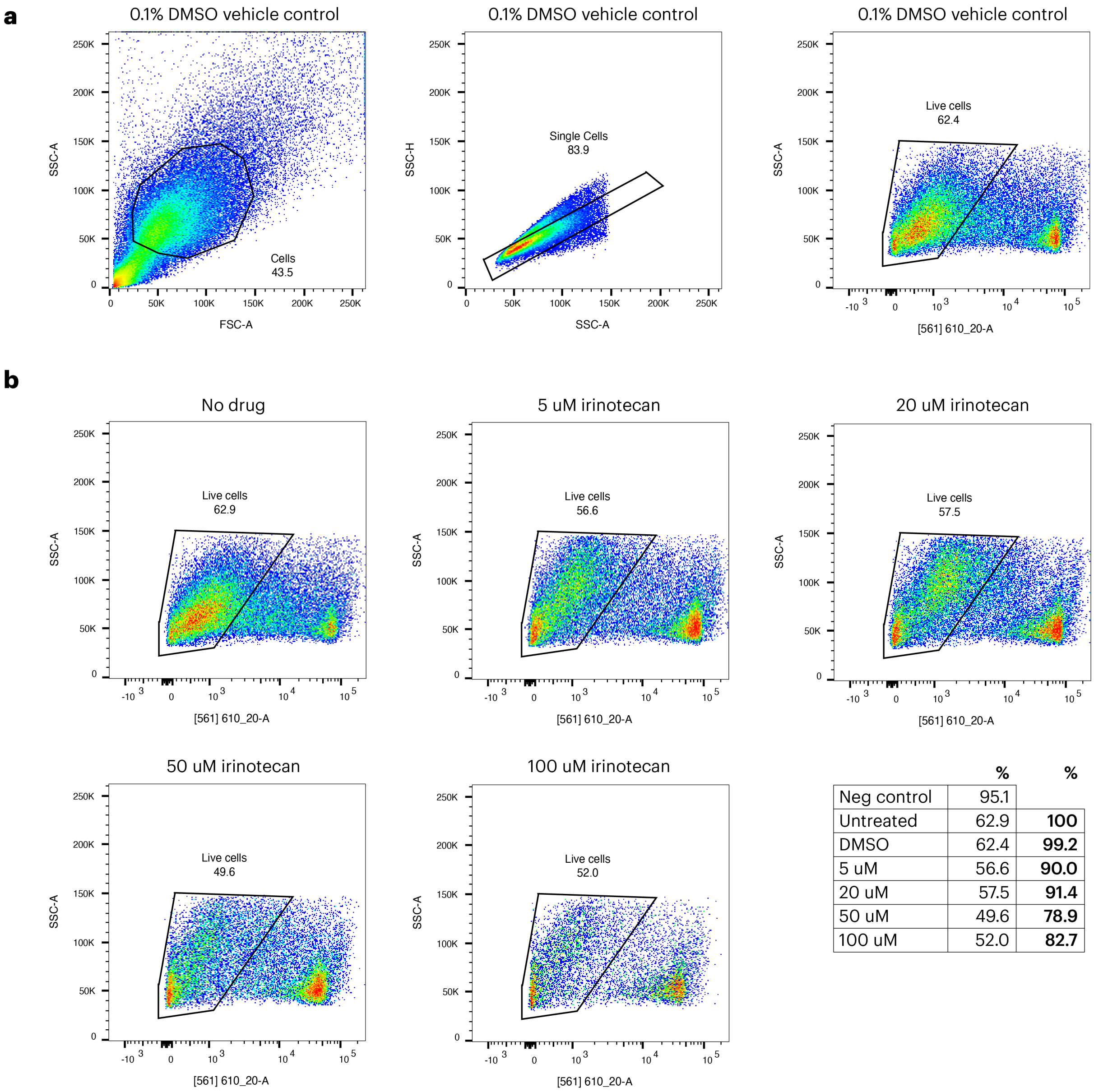

	%	%
Neg control	95.1	
Untreated	62.9	**100**
DMSO	62.4	**99.2**
5 uM	56.6	**90.0**
20 uM	57.5	**91.4**
50 uM	49.6	**78.9**
100 uM	52.0	**82.7**

Supplementary Figure 8. Flow cytometry plots indicating live cell populations with propidium iodide staining. 30,000 single cells were collected for each sample. **(a)** Representative gating strategy shown for the DMSO vehicle control. **(b)** Quantification of cell viability relative to untreated control.

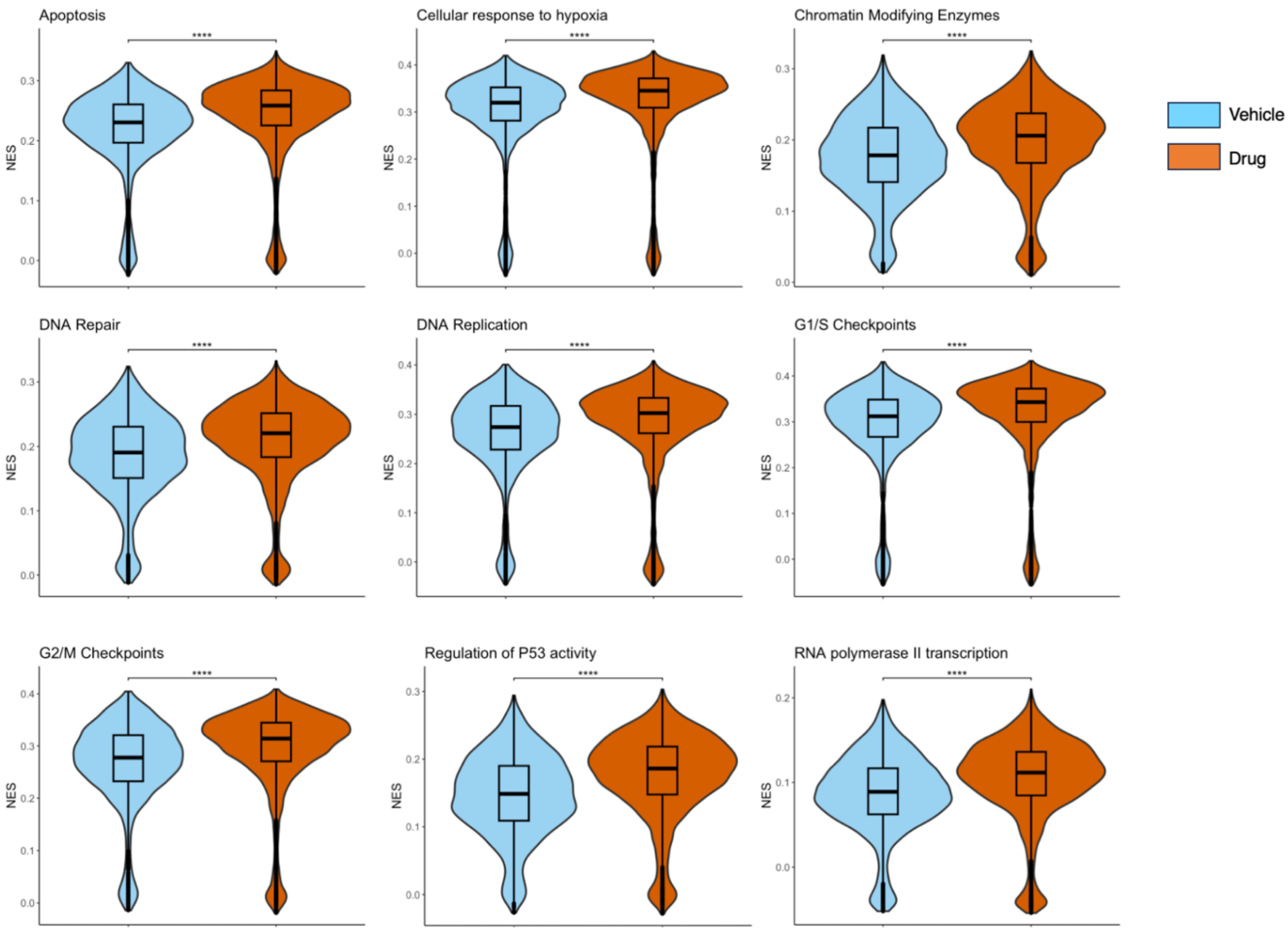

Supplementary Figure 9. Normalised enrichment scores for Reactome pathways in response to irinotecan treatment. Statistical significance calculated between vehicle control and irinotecan-treated cells; **** $p < 0.0001$.

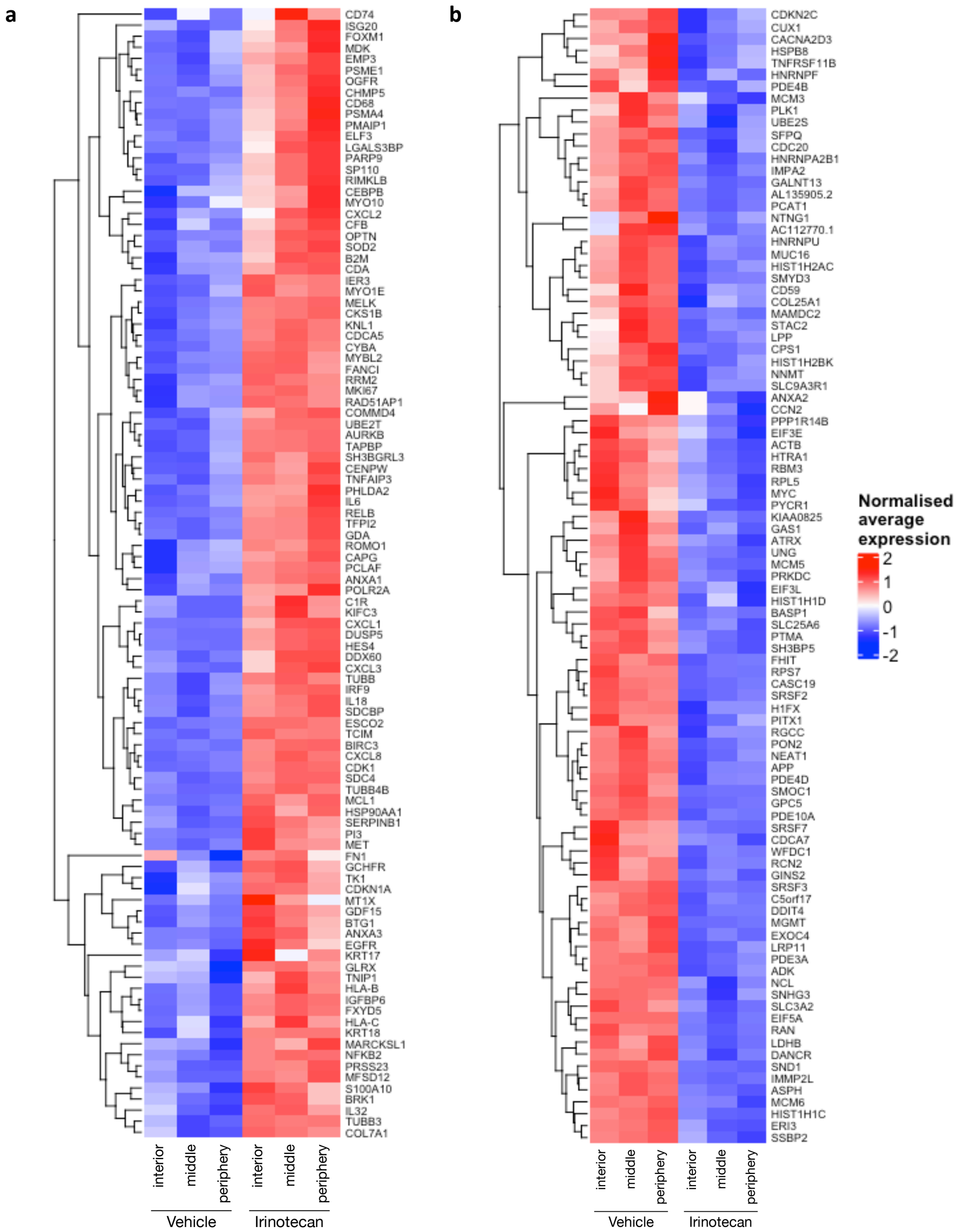

Supplementary Figure 10. Normalised average expression levels of differentially expressed genes, filtered for those genes that are affected by irinotecan treatment but do not display a spatially-dependent response. **(a)** Genes that are upregulated on exposure to the drug. **(b)** Genes that are downregulated on exposure.

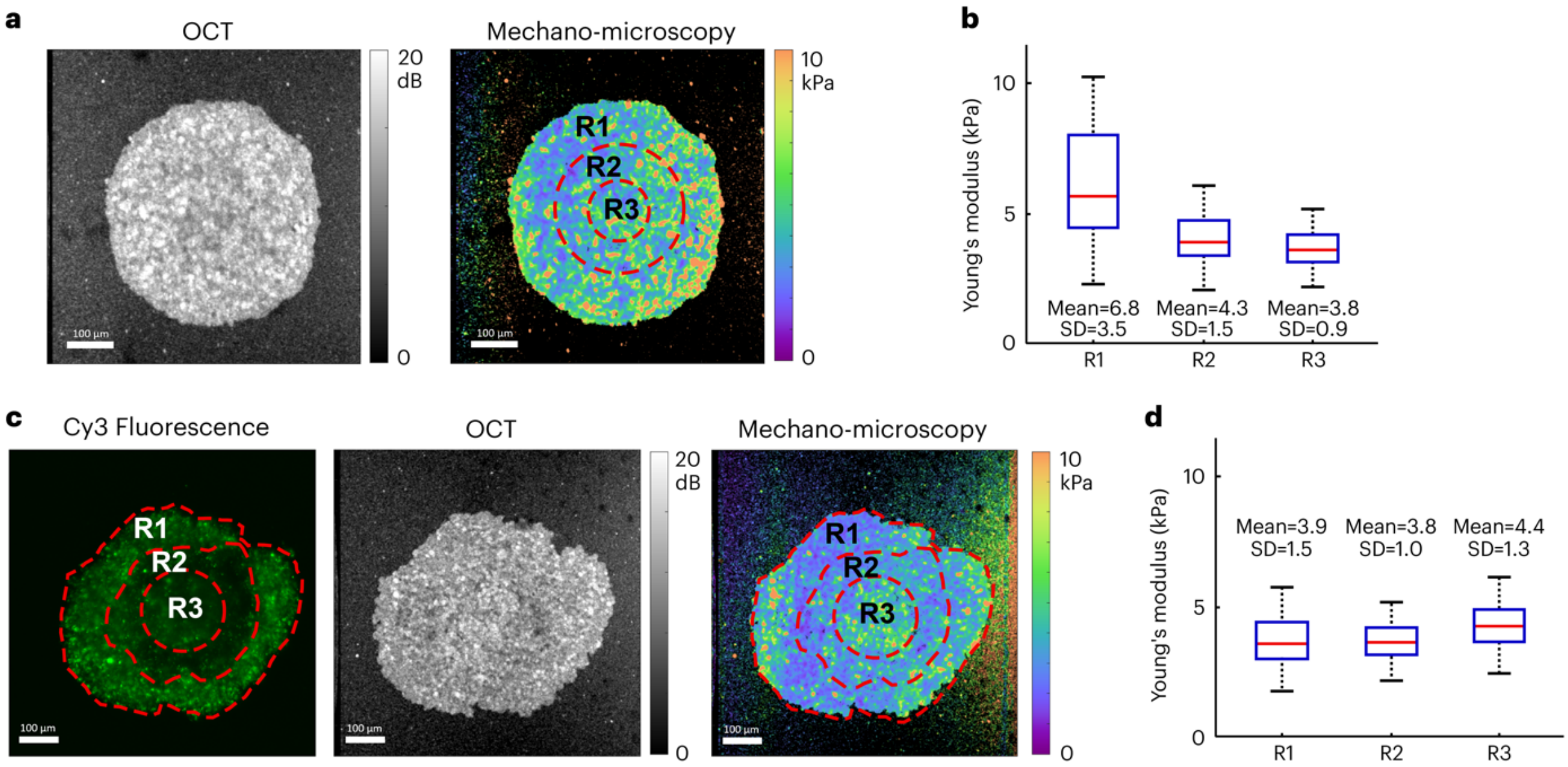

Supplementary Figure 11. Layered spheroids display uniform stiffness. **(a)** Optical coherence tomography (OCT) (left) and mechano-microscopy (right) images of mono-layer spheroid. **(b)** Box plot of elasticity measurements for pixels across the three arbitrarily selected regions of interest (R1-3) outlined in (a). Mean and standard deviation (SD) are also shown. **(c)** Fluorescence, OCT and mechano-microscopy images of three-layer spheroid. Cells in the core and outer layers were both tagged with Cy3-SBO to identify boundaries between the different layers since the setup for imaging in conjunction with elastography was a single-colour excitation system. **(d)** Elasticity (Young's modulus) measurements for pixels across the three layers (R1-3) outlined in (c) indicate little change in stiffness between the layers. Box and whisker plots are representative of $n = 2$ spheroids and display median (horizontal line), interquartile range (IQR, box) and 1.5×IQR (whisker), while values outside 1.5×IQR are not plotted.

Supplementary Tables

Supplementary Table 1. Feature barcode DNA sequences used in this study. Refer also to Supplementary Figure 1, which illustrates the position of the feature barcode within the full SBO sequence.

SBO ID	Feature barcode (5′ → 3′)	Used for labelling
SBO03	GTGCAAGAGTTGGCG	3-layer spheroid, vehicle-core
SBO05	TGGTGACAAGTATCT	3-layer spheroid, vehicle-middle
SBO07	CGAGGTACATCTTGT	3-layer spheroid, vehicle-periphery
SBO04	GAAGAAGCGTTATTC	3-layer spheroid, drug-core
SBO06	CACTCCTTGACAGGT	3-layer spheroid, drug-middle
SBO08	GCCAAGATCAGGTCC	3-layer spheroid, drug-periphery
SBO01-Cy5	AAGGCAGACGGTGCA	2-layer spheroid, interior
SBO14-Cy5	ACGAATCGGATACTA	2-layer spheroid, interior
SBO02-Cy3	GGCTGCGCACCGCCT	2-layer spheroid, periphery
SBO13-Cy3	ATAATCATTACGTGG	2-layer spheroid, periphery

Supplementary Table 2. Raw transcript counts of individual cells labelled either with unmodified SBO or Cy5-conjugated SBO.

	Min	Q1	Median	Mean	Q3	Max
SBO (no fluorophore)	515	16536	21494	21564	26634	52496
SBO–Cy5	504	17338	21989	23215	28808	67574
**All cells**	**504**	**17078**	**21788**	**22680**	**27892**	**67574**

Supplementary Table 3. Raw gene counts of individual cells labelled either with unmodified SBO or Cy5-conjugated SBO.

	Min	Q1	Median	Mean	Q3	Max
SBO (no fluorophore)	169	4180	4857	4597	5354	6991
SBO–Cy5	120	4242	4849	4726	5474	8489
**All cells**	**120**	**4234**	**4851**	**4684**	**5428**	**8489**

Supplementary Table 4. Raw SBO counts of individual cells labelled either with unmodified SBO or Cy5-conjugated SBO (rounded to the nearest integer).

	Min	Q1	Median	Mean	Q3	Max
SBO (no fluorophore)	152	606	772	882	1030	2892
SBO–Cy5	256	834	1370	1662	2062	11450
**All cells**	**152**	**734**	**1090**	**1408**	**1753**	**11450**

Supplementary Table 5. Raw transcript counts of individual cells isolated from two-layer spheroids.

	Min	Q1	Median	Mean	Q3	Max
Interior (Cy5-SBO)	503	12526	16148	18375	22385	88622
Periphery (Cy3-SBO)	507	14819	19213	20901	26197	58467
**All cells**	**503**	**12951**	**16914**	**19042**	**23434**	**88622**

Supplementary Table 6. Raw gene counts of individual cells isolated from two-layer spheroids.

	Min	Q1	Median	Mean	Q3	Max
Interior (Cy5-SBO)	339	3728	4409	4452	5255	7969
Periphery (Cy3-SBO)	340	4192	4884	4880	5721	7904
**All cells**	**339**	**3826**	**4530**	**4565**	**5400**	**7969**

Supplementary Table 7. Raw SBO counts of individual cells isolated from two-layer spheroids (rounded to the nearest integer).

	Min	Q1	Median	Mean	Q3	Max
Interior (Cy5-SBO)	51	230	564	1492	1549	29972
Periphery (Cy3-SBO)	67	337	571	1093	1149	23403
**All cells**	**51**	**262**	**567**	**1386**	**1427**	**29972**

Supplementary Table 8. Differentially expressed genes in two-layer spheroids. Average log_2FC represents expression level in peripheral layer relative to interior. Statistical significance for differential gene expression (p_{adj}) was reported by Seurat using non-parametric Wilcoxon rank sum test with Bonferroni correction.

Gene	Average log_2FC	p-value	p_{adj}
PGK1	-0.4164503	5.018237e-51	1.836725e-46
NQO1	0.3157012	4.149931e-48	1.518916e-43
FTL	0.2827381	3.785542e-47	1.385546e-42
BNIP3	-0.4473022	2.967747e-41	1.086225e-36
AKR1C3	0.3987517	4.221512e-40	1.545116e-35
ID3	0.2783516	6.894867e-39	2.523590e-34
CA9	-0.7937177	5.683418e-37	2.080188e-32
FAM162A	-0.3878044	6.854907e-34	2.508965e-29
MYL9	0.2556641	1.140053e-33	4.172708e-29
GPI	-0.3431416	3.117863e-32	1.141169e-27
LINC01411	0.2711669	1.517164e-28	5.552973e-24
NDRG1	-0.2999409	5.589421e-27	2.045784e-22
ALDH3A1	0.2905408	5.520260e-26	2.020470e-21
AKR1C2	0.4647274	1.071841e-24	3.923045e-20
CCN1	0.2505543	4.362710e-18	1.596795e-13
KRT17	0.2515342	4.816368e-14	1.762839e-09

Supplementary Table 9. Raw transcript counts of individual cells isolated from three-layer spheroids.

		Min	Q1	Median	Mean	Q3	Max
Vehicle	Interior	2446	12552	15475	17410	20456	49535
	Middle	5426	11696	16695	18282	21348	50713
	Periphery	1573	13590	18695	21902	26674	100605
Irinotecan	Interior	4568	14911	18802	20593	24792	54274
	Middle	5215	15876	19626	21743	25526	69320
	Periphery	2033	17090	23168	26183	32031	130156
**All cells**		**1573**	**15306**	**21011**	**23996**	**29451**	**130156**

Supplementary Table 10. Raw gene counts of individual cells isolated from three-layer spheroids.

		Min	Q1	Median	Mean	Q3	Max
Vehicle	Interior	1118	3586	4235	4342	5036	8603
	Middle	1973	3570	4478	4527	5196	7579
	Periphery	1057	3987	4820	4985	5868	9176
Irinotecan	Interior	1881	4394	5086	5084	5770	8521
	Middle	2411	4570	5172	5279	5909	8452
	Periphery	1211	4816	5725	5716	6666	9794
**All cells**		**1057**	**4379**	**5316**	**5370**	**6338**	**9794**

Supplementary Table 11. Raw SBO counts of individual cells isolated from three-layer spheroids (rounded to the nearest integer).

		Min	Q1	Median	Mean	Q3	Max
Vehicle	Interior	51	93	153	337	295	6192
	Middle	51	96	140	264	270	3772
	Periphery	51	380	1059	2483	2730	46562
Irinotecan	Interior	53	117	187	304	343	2625
	Middle	51	126	210	363	363	8374
	Periphery	51	481	1372	2749	3518	43664
**All cells**		**51**	**325**	**1009**	**2394**	**2839**	**46562**

Supplementary Table 12. Differentially expressed genes for mechanosensory, adhesion, and fibrotic markers in control spheroids. Statistical significance for differential gene expression (p_{adj}) was reported by Seurat using non-parametric Wilcoxon rank sum test with Bonferroni correction.

Gene	Average log2FC	*p*-value	p_{adj}	Layer
ACTG1	0.3136	2.403e-09	8.793e-05	interior
COL1A1	-1.2606	3.894e-05	1	interior
MYO10	-0.4234	6.589e-05	1	interior
FN1	0.8804	0.0012	1	interior
CD276	-0.4345	0.0046	1	interior
SMAD7	-0.5189	0.0060	1	interior
ITGAV	-0.2721	0.0110	1	interior
MYO1B	0.5808	0.0382	1	interior
ITGB1	0.2557	0.0390	1	interior
CITED2	0.3286	0.0471	1	interior
ACTG1	0.2630	2.116e-07	0.0077	middle
TUBA1A	-0.3902	4.734e-04	1	middle
TUBG2	-0.6444	0.0022	1	middle
TUBG1	-0.2631	0.0034	1	middle
SMAD7	-0.3351	0.0142	1	middle
TIMP1	0.3151	0.0179	1	middle
COL1A1	-0.3628	0.0263	1	middle
ACTG1	-0.3013	1.026e-15	3.753e-11	periphery
MYO10	0.3332	5.601e-06	0.2050	periphery
COL1A1	0.7421	7.859e-06	0.2876	periphery
TUBA1A	0.3043	9.219e-06	0.3374	periphery
SMAD7	0.4642	1.822e-04	1	periphery
TUBG1	0.2543	1.887e-04	1	periphery
TUBG2	0.4202	8.086e-04	1	periphery
FN1	-0.6310	0.0013	1	periphery
CITED2	-0.2757	0.0150	1	periphery

List of genes tested: *COL1A1, COL1A2, COL3A1, FN1, FMOD, POSTN, VCAM1, CCL2, SIRT1, KLF5, PPARG, AKT1, SHH, SMAD7, TGFB1, CTNNB1, MMP2, ITGB1, PLAUR, MMP14, NOX4, CADM1, CD2, CD226, CD274, CD276, CD28, CD34, CD4, CD40, CD58, CD6, CD80, CD8A, CD8B, CD99, CDH1, CDH2, CDH3, CDH4, CDH5, CDH15, ICAM1, ICAM2, ICAM3, ITGA4, ITGA6, ITGA8, ITGA9, ITGAL, ITGAM, ITGAV, ITGB2, ITGB7, ITGB8, SDC1, SDC2, SDC3, SDC4, CITED2, ACTA1, ACTA2, ACTB, ACTBL2, ACTC1, ACTG1, ACTG2, TUBA1A, TUBA1B, TUBA1C, TUBB, TUBB1, TUBB2A, TUBB2B, TUBB3, TUBB4A, TUBB4B, TUBB6, TUBB8, TUBD1, TUBE1, TUBG1, TUBG2, MYO1A, MYO1B, MYO1C, MYO1D, MYO1E, MYO1F, MYO1G, MYO1H, MYO3A, MYO3B, MYO5A, MYO5B, MYO5C, MYO6, MYO7A, MYO7B, MYO9A, MYO9B, MYO10, MYO15A, MMP1, MMP7, MMP8, MMP9, MMP10, MMP11, MMP12, MMP13, MMP15, MMP16, MMP17, TIMP1, TIMP2, TIMP3, TIMP4, TNC.*
