# Supplementary material for: Single-cell RNA sequencing profiles drug activity within spatially engineered 3D cultures
Source: Nat Commun. 2026 Jun 19;17:7735. doi: 10.1038/s41467-026-74587-8 (PMC13434128; doi:10.1038/s41467-026-74587-8)
Supplement: Supplementary file 2 [file 41467_2026_74587_MOESM2_ESM.pdf]

nature portfolio

Corresponding author(s): Cameron W. Evans

Last updated by author(s): May 21, 2026

Reporting Summary

Nature Portfolio wishes to improve the reproducibility of the work that we publish. This form provides structure for consistency and transparency in reporting. For further information on Nature Portfolio policies, see our Editorial Policies and the Editorial Policy Checklist.

Statistics

For all statistical analyses, confirm that the following items are present in the figure legend, table legend, main text, or Methods section.

n/a	Confirmed	
☐	☒	The exact sample size (*n*) for each experimental group/condition, given as a discrete number and unit of measurement
☐	☒	A statement on whether measurements were taken from distinct samples or whether the same sample was measured repeatedly
☐	☒	The statistical test(s) used AND whether they are one- or two-sided<br>*Only common tests should be described solely by name; describe more complex techniques in the Methods section.*
☒	☐	A description of all covariates tested
☐	☒	A description of any assumptions or corrections, such as tests of normality and adjustment for multiple comparisons
☒	☐	A full description of the statistical parameters including central tendency (e.g. means) or other basic estimates (e.g. regression coefficient) AND variation (e.g. standard deviation) or associated estimates of uncertainty (e.g. confidence intervals)
☐	☒	For null hypothesis testing, the test statistic (e.g. *F*, *t*, *r*) with confidence intervals, effect sizes, degrees of freedom and *P* value noted<br>*Give P values as exact values whenever suitable.*
☒	☐	For Bayesian analysis, information on the choice of priors and Markov chain Monte Carlo settings
☒	☐	For hierarchical and complex designs, identification of the appropriate level for tests and full reporting of outcomes
☒	☐	Estimates of effect sizes (e.g. Cohen's *d*, Pearson's *r*), indicating how they were calculated

Our web collection on statistics for biologists contains articles on many of the points above.

Software and code

Policy information about availability of computer code

Data collection	For single-cell RNA sequencing, cells were loaded onto a 10x Genomics Chromium Controller, Chip K (PN 1000286) and libraries were prepared using the Chromium Next GEM single cell 5' kit v2 and 5' Feature Barcode kit. Illumina iSeq 100 platform was used for initial QC of libraries and deep sequencing was conducted using Illumina NovaSeq 6000. For immunofluorescence, images were captured using a Nikon A1 RMP confocal microscope and NIS Elements software. Flow cytometry data was collected with BD SORP Fortessa and BD FACSDiva software.
Data analysis	• Sequencing analysis: Fastq files were generated with Illumina bcl2fastq v2.20.0.422, allowing 1 mismatch in each index. 10x Genomics Cell Ranger v7.0.0 was used for transcript alignment and generation of gene count and feature barcode matrices. Output from CellRanger were used in downstream analysis using R studio v2022.12.0+353 or later, and the following packages; ClusterProfiler v4.6.2, GSEABase v1.60.0, ReactomePA v1.42.0, and Seurat v4.3.0.1.<br>• Immunofluorescence analysis: Image analyses were performed using Fiji ImageJ v.2.9.0 or later.<br>• Flow cytometry analysis: Flow cytometry data were analysed using FlowJo v10.

For manuscripts utilizing custom algorithms or software that are central to the research but not yet described in published literature, software must be made available to editors and reviewers. We strongly encourage code deposition in a community repository (e.g. GitHub). See the Nature Portfolio guidelines for submitting code & software for further information.

Data

Policy information about availability of data

All manuscripts must include a data availability statement. This statement should provide the following information, where applicable:

- Accession codes, unique identifiers, or web links for publicly available datasets
- A description of any restrictions on data availability
- For clinical datasets or third party data, please ensure that the statement adheres to our policy

• Single-cell sequencing datasets generated in this study have been deposited in the National Centre for Biotechnology Information (NCBI) Gene Expression Omnibus (GEO) under accession number GSE245416 [https://www.ncbi.nlm.nih.gov/geo/query/acc.cgi?acc=GSE245416].
• Processed data used in the figures are provided with this paper as a Source Data file.
• Raw data have been deposited at doi:10.5281/zenodo.20373611 [https://doi.org/10.5281/zenodo.20373611].
• The R code used for data processing is available at doi:10.5281/zenodo.20373611 [https://doi.org/10.5281/zenodo.20373611].
• D-score software is available at doi:10.5281/zenodo.20375079 [https://doi.org/10.5281/zenodo.20375079].

Research involving human participants, their data, or biological material

Policy information about studies with human participants or human data. See also policy information about sex, gender (identity/presentation), and sexual orientation and race, ethnicity and racism.

Reporting on sex and gender	n/a
Reporting on race, ethnicity, or other socially relevant groupings	n/a
Population characteristics	n/a
Recruitment	n/a
Ethics oversight	n/a

Note that full information on the approval of the study protocol must also be provided in the manuscript.

Field-specific reporting

Please select the one below that is the best fit for your research. If you are not sure, read the appropriate sections before making your selection.

- [x] Life sciences
- [ ] Behavioural & social sciences
- [ ] Ecological, evolutionary & environmental sciences

For a reference copy of the document with all sections, see nature.com/documents/nr-reporting-summary-flat.pdf

Life sciences study design

All studies must disclose on these points even when the disclosure is negative.

Sample size	Sample sizes were one run for single-cell RNA sequencing experiments based on cost. No statistical methods were used to predetermine sample size. See also 'Replication' below.
Data exclusions	No data was excluded from experiments, but small subpopulations of cells exhibiting signs of widespread loss of gene regulation were excluded during data analysis. Each of these subpopulations has been explicitly mentioned in the manuscript.
Replication	• For in vitro experiments, at least two biological/technical replicates were conducted, as specified in the figure legends. In general, single spheroids were grown and treated in independent wells, after which 4-5 spheroids from the same experimental group were combined into one sample before cryopreservation, embedding, and sectioning. Where sections contained more than one spheroid, these were regarded as independent replicates since they were grown and treated in separate wells. We aimed for at least 2 biological replicates, each producing images of at least 2 spheroids.<br>• Flow cytometry results are representative of 2 biological replicates.<br>• For multiplex sequencing, an equal number of cells from each sample were included at a concentration of 2,000 cells/µl. Spheroid sequencing experiments were not repeated due to cost.
Randomization	2D cell culture in wellplates and 3D spheroids were assigned at random to control or treatment groups.
Blinding	Experimenters were generally not blinded as to control and treatment groups, except in sequencing experiments. For sequencing, pooled and barcoded cells were submitted as described in 'Replication'; initial data processing (Cell Ranger and D-score demultiplexing) was carried out using supplied SBO sequences but researchers were blinded as to which experimental groups each of these sample barcodes represented. Further analyses of sequencing results were not blinded.

Reporting for specific materials, systems and methods

We require information from authors about some types of materials, experimental systems and methods used in many studies. Here, indicate whether each material, system or method listed is relevant to your study. If you are not sure if a list item applies to your research, read the appropriate section before selecting a response.

Materials & experimental systems

n/a	Involved in the study
☐	☒ Antibodies
☐	☒ Eukaryotic cell lines
☒	☐ Palaeontology and archaeology
☒	☐ Animals and other organisms
☒	☐ Clinical data
☒	☐ Dual use research of concern
☒	☐ Plants

Methods

n/a	Involved in the study
☒	☐ ChIP-seq
☐	☒ Flow cytometry
☒	☐ MRI-based neuroimaging

Antibodies

Antibodies used	Rabbit anti-human Ki67 clone SP6 monoclonal (Abcam, ab16667)<br>Goat anti-rabbit IgG Alexa Fluor 488 (Abcam, ab150077)
Validation	Ki-67 antibody was knockout validated for confirmed specificity by the vendor. 'No primary antibody' controls for non-specific staining were confirmed by us.

Eukaryotic cell lines

Policy information about cell lines and Sex and Gender in Research

Cell line source(s)	HeLa cells were sourced from ATCC.
Authentication	Cell lines were not authenticated.
Mycoplasma contamination	Culture supernatants and pellets were tested and confirmed to be free of detectable Mycoplasma by PCR analysis.
Commonly misidentified lines (See ICLAC register)	HeLa is not reported as a commonly misidentified cell line.

Plants

Seed stocks	n/a
Novel plant genotypes	n/a
Authentication	n/a

Flow Cytometry

Plots

Confirm that:

☒ The axis labels state the marker and fluorochrome used (e.g. CD4-FITC).

☒ The axis scales are clearly visible. Include numbers along axes only for bottom left plot of group (a 'group' is an analysis of identical markers).

☒ All plots are contour plots with outliers or pseudocolor plots.

☒ A numerical value for number of cells or percentage (with statistics) is provided.

Methodology

Sample preparation	Cells and/or spheroids were washed with 1xPBS and dissociated with TrypLE. After centrifugation (300g, 5 min), collected cells were resuspended in 1xPBS + 6% v/v FBS + 2 mM EDTA.
Instrument	BD Canto II or BD SORP Fortessa LSR
Software	Data were acquired using BD FACSDiva v8.0.2 and analysed post-collection with FlowJo v10.
Cell population abundance	Unless specified otherwise, >10,000 single cells were analysed per sample.
Gating strategy	Samples were all gated using the same strategy; first, on FSC-A vs SSC-A to select for cells and exclude cellular debris, and second, on SSC-H vs SSC-A to select single cells and exclude multiplets. For quantification of fluorescent markers, a threshold was set using a negative control sample.

☒ Tick this box to confirm that a figure exemplifying the gating strategy is provided in the Supplementary Information.
