# Supplementary material for: Single-cell RNA sequencing profiles drug activity within spatially engineered 3D cultures
Source: Nat Commun. 2026 Jun 19;17:7735. doi: 10.1038/s41467-026-74587-8 (PMC13434128; doi:10.1038/s41467-026-74587-8)
Supplement: Supplementary file 3 [file 41467_2026_74587_MOESM3_ESM.pdf]

nature portfolio

Peer Review File

Single-cell RNA sequencing profiles drug activity within spatially engineered 3D cultures

Corresponding Author: Dr Cameron Evans

This file contains all reviewer reports in order by version, followed by all author rebuttals in order by version.

Version 0:

Reviewer comments:

Reviewer #1

(Remarks to the Author)
The manuscript presented by Jessica King et al; describes a methodology allowing to incorporate molecular DNA barcodes by transfection in mammalian cells that are then used for 3-dimensional cultures. Importantly, by using short barcode oligonucleotides (SBOs) conjugated to fluorophores and allowing to capture messanger RNA, this barcoding strategy has been used for building Hela spheroids presenting multiple layers of barcoded cells. This strategy has been named scTECH-seq for "single cell transfection-enabled cell hashing".

The most remarkable aspect of this strategy is the fact that it combines the power of single-cell transcriptomics with a physical spatial information across the assembled spheroid thanks to the layer information obtained by imaging.

Main comments:
- A key aspect of single-cell transcriptomics assays is to count with a unique molecular barcode per cell; or the way in which the technique is presented, it is not clear how is possible to ensure that a unique barcode is transfected per cell? This step is not essential for stratifying the different layers (because it is based on the conjugated fluorophore), but it is essential when analyzing the obtained transcripts by NGS.

- Since the different layers are added by consecutive deposition of cells over the 3D culture, the study does not consider the effect of cell proliferation and differentiation during the process. For instance, what would be the consequence of building such layered 3D cultures with embryonic stem cells that could be pushed to differentiate for instance in nervous tissue? This includes the viability of the transfected barcodes over days, but also cellular migration across layers. While this question could appear out of the focus of this manuscript, addressing this point might provide a larger application panel

(Remarks on code availability)

Reviewer #2

(Remarks to the Author)
Reviewer comments for "Single-cell RNA sequencing profiles drug activity within spatially engineered 3D cultures".

Jessica J. King et al presents a transfection-based method for constructing cellular architectures in 3D cultures, using two-color coded DNA barcodes to encode the spatial positioning of cells. This method, termed single cell transfection-enabled cell hashing (scTECH-seq), facilitates imaging and multiplex single-cell RNA sequencing (scRNA-seq), providing three-layer maps of gene expression. They demonstrated that SBOs, transfected individually into HeLa cells and used to construct multi-layered spheroids with spatial features, can de-multiplex single-cell gene expression profiles by three layers or two spheroid layers. Furthermore, they tested the variability in drug response across different spaces through stimulation experiments with the topoisomerase inhibitor irinotecan.The technique also includes independent set of imaging with mechano-microscopy to capture spatial variations in tissue elasticity. The study claims that 3D cultures display heterogeneous responses to drugs, influenced by factors such as drug diffusion gradients, differences in metabolism, nutrient supply, and cellular stressors.

Strength of this methodology is unclear. The authors repeatedly emphasize the problem of spatial resolution in the past spatial omics analysis. While this simple model is useful for verifying the method's robustness, for analytical purposes that aim to examine expression profiles from a spatial perspective, structures with greater heterogeneity or mosaic compositions would be more beneficial (PMID: 34875362 and 37533100). Methods have already been proposed for examining single-cell level spatial characteristics in more complex spheroid structures, such as heterogeneity and interactions with the surrounding environment (PMID: 35273957 and 33369114). Additionally, there are emerging modalities like Xenium, and others that can overcome resolution issue but none of these are discussed nor benchmarked. Perhaps more critically, this reviewer didn't see any "direct" resolution-related dataset since the spatial aspects were only controlled by spheroid formation process. There is computationally-inferred statistical spatial mapping methods are available and need to be discussed.

The method involves multiple complex manually controlled engineering steps, including transfection, multi-layered spheroid formation, and scRNA-seq, which may limit its accessibility to laboratories with less technical expertise or resources. Additionally, the applicability of this system is only tested in one immortal line (HeLa) cell based model which are not typically used in 3D format.

Insights gained from Irinotecan exposures mostly validates can be expected from traditional biology toolsets except that spatially restricted gene expression patten. For example, PARP14, IFITM2 and BCAP31 have been identified as a DEG set, however, none of these are spatially validated in their three layer spheroid culture system.

Mechanobiological assertion is of interest aspect however, this section is very misleading since elasticity measurement is completely independent of scRNAseq itself. I don't see any added benefit as a main part of stories unless more precise spatial mechanocharacter is profiled with transcriptomes. Overall, the scTECH-seq method is certainly a new development in the field of spatial transcriptomics, however, the applicability and uniqueness high-resolution spatial analysis of gene expression in 3D cultures.

Addressing the weaknesses specified below would help the study accurately verify the validity of this method.

Major comments are provided here:
1. SBO degradation and diffusion: The study mentions the potential degradation of SBOs over time, which could compromise the accuracy of spatial encoding and demultiplexing in longer experiments. However, I'm not entirely convicted by the discussion. For example, inner, middle degradation effects are heavily biased by cell death and stress response and I'm not entirely sure this interpretation only comes from degradation. None is discussed about potential barcode diffusion (for example, double positivity). These two aspects need to be quantitatively addressed by additional set of experiments.
2. Potential bias in cell types: The use of transformed cancer cells as a model system may not fully capture the heterogeneity of other cell types or tissues, potentially limiting the generalizability of the findings. Particularly this method is highly complex and not entirely clear is applicable when it comes to primary cells, stem cell-derived cell or non-epithelial lineages are includedA lack of experimental designs aimed at diverse applications such as comparative and co-culture models using various cancer cell lines, drug responses and toxicity fields using hepatic spheroid made it difficult to evaluate the advancements and potential of the scTECH-seq method appropriately.
3. Limited drug studies: Abstract and title is very bold and sound in characterizing spatially heterogeneous responses to drugs, however, the paper ended up only focusing on irinotecan, a single chemotherapeutic agent. As noted, the most of the biological insights are confirmatory and not requires spatial analysis. Broader studies involving multiple drugs would provide a more comprehensive understanding of the method's utility.
4. Spatial validation: Identified spatially biased genes need a in situ RNA staining to ascertain their discoveries including drug induced spatially heterogenous responsive genes: PARP14, IFITM2 and BCAP31.
5. Extensive rewrite: The readers will get confused about the resolution issues by comparing low resolution approach because this paper didn't precisely address resolution problem and no comparable data is presented. The important comparator will be to discuss weakness droplet-sequencing based statistical spatial reconstruction methodologies and advantage of the authors' method.

Other more specific comments:
Line 43-48: Besides the technology platform described by the authors, methods such as Stereo-seq (PMID: 37337030) and Seq-Scope (PMID: 34115981) are reported to achieve spatial resolution at the nanometer scale (~500 nm). Additionally, algorithms that integrate scRNA-seq and Spatial transcriptome data to deconvolute at the single-cell level have been reported (PMID: 38040768). The authors should also discuss these competitive technologies that address the issue of resolution and argue the advantages of scTECH-seq.
Line 136-138: The spatially defined gene expression patterns are only based on the macro expression profiles of artificially layered spaces. Is the spatial resolution really high when considering single-cell level spatial decomposition? Can we identify subpopulations that form within or move out of the coded spaces?
Line 182-184: Is it possible to quantitatively compare the relationship between the layers in the spheroid by overlapping the Fucci fluorescence signal (mVenous-hGem) and the SN-38 signal? The correlation between irinotecan metabolism and cell cycle changes per layer should be mentioned along with the deconvolution of scRNA-seq data (Figure 4d).
Line 250-255: The differences in drug response correlation between 3D spheroid and 2D monolayer cultures could potentially be due to technical issues. At least, data in 3D spheroids should be validated against Camptothecin or some topoisomerase II inhibitors.
Lines 268-272: Although the data suggesting uniform elasticity in multilayer spheroids as opposed to monolayer spheroids present an interesting insight, making a connection between these mechanobiological characteristics and single-cell gene expression profiles may be somewhat of a leap. To reiterate, are the interactions between cells uniformly distributed across

layers in such structures? Particularly, the expression and pairing pattern of mechanosensing receptors, adhesion molecules and inflammatory/fibrosis responsive genes would be informative. Superimposing spatial information about the structure with intercellular relationship information could enhance the significance of conducting spatial analysis at the cellular layer level.

(Remarks on code availability)

Reviewer #3

(Remarks to the Author)
I co-reviewed this manuscript with one of the reviewers who provided the listed reports. This is part of the Nature Communications initiative to facilitate training in peer review and to provide appropriate recognition for Early Career Researchers who co-review manuscripts.

(Remarks on code availability)

Reviewer #4

(Remarks to the Author)
I co-reviewed this manuscript with one of the reviewers who provided the listed reports. This is part of the Nature Communications initiative to facilitate training in peer review and to provide appropriate recognition for Early Career Researchers who co-review manuscripts.

(Remarks on code availability)

Reviewer #5

(Remarks to the Author)
In this work, Evans and colleagues performed spatial single-cell gene expression analyses in spheroids generated by pre-barcoded cell seeding in a layer-by-layer fashion. This method allows analysis of heterogeneous responses of different layers to therapies. It provides an approach to addressing the positional influence in 3D cell culture systems and holds the potential for future extension to high-throughput 3D bioprinting. Currently, it is important to understand how different regions of the tumor spheroid respond to therapies and to assess how the drug penetrates and affects cells at different spheroid levels. Additional experiments should be performed to strengthen the manuscript.
Major:
1. The spheroid was generated by growing spheroid one layer every 24 hours with pre-barcoded cells, which is not the common protocol for spheroid formation. People would not be able to analyze spatial positioning difference if they do not create spheroid layer-by-layer. What is the advantage of the layered spheroid?

2. The 2-layer spheroid is less than150 m while the 3-layer spheroid is approximately 400 m. Large spheroids have been reported to have ki-67 positive signals restricted to the outmost cell layers. The ki67 staining for the 3-layer spheroid exhibits homogenous distribution, which is not expected. Evidence should be provided to determine whether it is due to the spheroid size or to the layer-by-layer operation.

3. Seeding each layer requires a 24-hour interval. How many layers can one do at most? Will the interior cells die if the culturing is too long?

4. The reference #36 used dye penetration to identify different layers of spheroids. It is recommended to compare your method with this method. The drug response study can also be done using this method and compared with the current study.

5. Please provide evidence that cells grow after seeding and if so, the newly divided cells still contain SBOs.

6. What is the advantage of the dendritic polymer transfection? Can you compare it to the widely used method such as lipid, lipofectamine or nucleofection? How did you determine your transfection conditions with the polymer? There isn’t a mature protocol for this kind of transfection.

7. Does the cluster 4 (dead cells) in figure 2b come from the interior? Additional figures showing the three layers instead of 4 clusters will better illustrate the distribution of the three layers in the 4 clusters.

8. The cell cycle category used in figures 2, 3 and 4 are different. It is unclear why the authors included early-S phase in figure 2 how they distinguish the early-S fraction?

9. There is a group of genes activated in the interior but not in the periphery upon irinotecan treatment (figure 4f, top). It is important to provide explanation for this phenomenon.

10. In Supplementary figure S7, it seems that lots of cell died upon irinotecan treatment, even the vehicle results in a significant portion of dead cells. The flow cytometry data is not convincing, and live and dead cells percentage should be indicated in the figure.

11. It'd be interesting to detect and measure irinotecan-induced TOP1 cleavage complexes (TOP1cc) for each spheroid layers as stable TOP1cc induction is the primary mechanism of action of TOP1 inhibitors. Please refer to PMID: 37353483 for TOPcc detection in 3D organoids.

Minor:
1. No scale bar in figure 2a.
2. Please mark consistently for figures. For example, in supplementary figure S9, mark "control" and "drug" the same way as the main figures.

(Remarks on code availability)

Version 1:

Reviewer comments:

Reviewer #1

(Remarks to the Author)
The authors replied to all my comments.

(Remarks on code availability)

Reviewer #2

(Remarks to the Author)
First, I acknowledge the improvements made in addressing Comment 1 (barcode distribution and contamination) and Comment 8 (FACS-based quantification of SN-38 / Fucci). These changes appropriately correct some of the overstatements in the original submission and strengthen the discussion of feasibility and analytical framework.
However, I am frankly surprised by the absence of any new experimental evidence for several critical points. Specifically, issues raised in Comment 2 (cell types), Comment 3 (drug types), Comment 4 (RNA spatial localization), Comment 9 (counterpart data using inhibitors), and Comment 10 (mechanobiology-related observations) were only addressed by softening the language, without providing additional data. While I understand the authors' reasoning, this approach substantially weakens the claim of methodological generalizability.
The lack of in situ RNA validation (Comment 4) is particularly concerning, as these omissions leave key aspects of the dataset unvalidated. This raises questions about the robustness of the conclusions and feels somewhat inconsistent with the emphasis placed on these points in the original manuscript.
Regarding Comments 5 and 6 on spatial resolution: I appreciate the authors' acknowledgment of limitations in spheroid or organoid contexts. Nevertheless, published studies demonstrate that spatial transcriptomics can be applied even at the spheroid scale, with internal population structures resolved to a meaningful degree (see PNAS 2024, Nature 2023). While scTECH-seq may capture 3D layers within spheroids, section-based approaches could likely achieve similar resolution. If the authors intend to claim single-cell resolution in Fig. 4a–c (Comment 7), benchmarking against a competing section-based technology would be highly valuable, even if ambitious.
In summary, the key question is whether the toned-down novelty claims sufficiently compensate for the lack of benchmarking and validation. At present, these gaps remain significant and will likely influence the decision.

(Remarks on code availability)

Reviewer #3

(Remarks to the Author)
I co-reviewed this manuscript with one of the reviewers who provided the listed reports. This is part of the Nature Communications initiative to facilitate training in peer review and to provide appropriate recognition for Early Career Researchers who co-review manuscripts.

(Remarks on code availability)

Reviewer #4

(Remarks to the Author)

I co-reviewed this manuscript with one of the reviewers who provided the listed reports. This is part of the Nature Communications initiative to facilitate training in peer review and to provide appropriate recognition for Early Career Researchers who co-review manuscripts.

(Remarks on code availability)

Reviewer #5

(Remarks to the Author)
The major concerns raised in the previous review have been clearly and satisfactorily addressed.

In particular, the advantages of the layered spheroid model are now more clearly articulated and effectively highlighted. The earlier confusion regarding the relative sizes of the two-layer and three-layer spheroids—stemming from an incorrect scale bar—has been appropriately corrected. With this issue resolved, it is now evident that the spheroids are comparable in size, which greatly improves the clarity and interpretability of the layered spheroid approach.

The additional clarifications concerning spheroid growth dynamics and patterns of cell death throughout development further strengthen the manuscript. The observation that irinotecan treatment exerts differential effects between the interior and peripheral regions of the spheroid is particularly intriguing. While the precise underlying mechanisms remain to be fully defined, this does not diminish the overall value or utility of the model.

Overall, the manuscript has been substantially improved, and we have no remaining major concerns.

(Remarks on code availability)

Open Access This Peer Review File is licensed under a Creative Commons Attribution 4.0 International License, which permits use, sharing, adaptation, distribution and reproduction in any medium or format, as long as you give appropriate credit to the original author(s) and the source, provide a link to the Creative Commons license, and indicate if changes were made.
In cases where reviewers are anonymous, credit should be given to 'Anonymous Referee' and the source.
The images or other third party material in this Peer Review File are included in the article's Creative Commons license, unless indicated otherwise in a credit line to the material. If material is not included in the article's Creative Commons license and your intended use is not permitted by statutory regulation or exceeds the permitted use, you will need to obtain permission directly from the copyright holder.
To view a copy of this license, visit https://creativecommons.org/licenses/by/4.0/

Reviewer #1

Edits to the manuscript are highlighted in blue.

1. A key aspect of single-cell transcriptomics assays is to count with a unique molecular barcode per cell; or the way in which the technique is presented, it is not clear how is possible to ensure that a unique barcode is transfected per cell? This step is not essential for stratifying the different layers (because it is based on the conjugated fluorophore), but it is essential when analyzing the obtained transcripts by NGS.

   ✓ To clarify the above, there are actually two different 'barcodes' used during sequencing:

   i. Each individual cell receives a unique molecular identifier (UMI) as part of 10X Chromium single-cell workflow, which serves as a cellular barcode; UMIs are incorporated during library preparation and are unique to each cell analysed.

   ii. All cells within a given spheroid layer have been transfected with the same SBO (which may also be fluorescently labelled), which serves as a 'feature' barcode for cells within that layer. SBOs are not unique to each cell, but are unique between spheroid layers and samples.

   Transcripts and SBOs are captured and tagged with the same UMI during library preparation. After sequencing, reads with the same UMI are grouped together as they originate from the same cell. The transcript set reveals the gene expression profile, while the associated SBO tells us which spheroid layer and treatment condition the cell came from. We have amended the text to make this clearer:

   > ***Lines 69–71***
   > *"In this scheme, each individual cell is assigned a unique molecular identifier (UMI) during single-cell library preparation via the 10X Genomics Chromium platform, while SBOs serve as feature barcodes that distinguish different samples of cells."*

2. Since the different layers are added by consecutive deposition of cells over the 3D culture, the study does not consider the effect of cell proliferation and differentiation during the process. For instance, what would be the consequence of building such layered 3D cultures with embryonic stem cells that could be pushed to differentiate for instance in nervous tissue? This includes the viability of the transfected barcodes over days, but also cellular migration across layers. While this question could appear out of the focus of this manuscript, addressing this point might provide a larger application panel.

   ✓ We agree that the dynamics of proliferation, differentiation, and migration would be critical when applying this approach to stem cells or more plastic cell types:

   iii. Our study focused on short-term experiments in HeLa cells, and we observed a decrease in the number of SBOs per cell over 72–96 h (as presented in the Discussion), which we suggested might limit longer-term studies in immortalised cells. However, in line with Reviewer 2's request (point 1), we have now quantified two different SBO sequences in HeLa cells over time by qPCR, obtaining very similar results for both sequences (inserted as Supplementary Figure S2). The apparent half-life of SBOs in HeLa cells (2D culture) is 28 ± 4 h. As this is identical to the cell population doubling time, we conclude that apparent barcode 'loss' is principally due to proliferation/cell division, effectively halving the number of SBOs per cell each time the cell population doubles in number. This is expected because the SBOs contain phosphorothioate modifications to limit nuclease-mediated degradation. Therefore, we infer that slower-dividing cells could be analysed over longer time periods. Studies involving terminal differentiation in which cells stop dividing may support even longer-term barcoding in spheroids or developing organoids, which could be an exciting application for future exploration.

   iv. Migration of cells within a spheroid could indeed lead to a loss of spatially resolved tagging. However, our approach still has value over longer-term studies, since our pioneering use of fluorescent-tagged SBOs would allow any such migration of barcoded cell subpopulations to be visualised, while also supporting single-cell RNA-seq analysis of barcoded cell populations. Thus, migration and differentiation of cellular subpopulations could potentially be monitored

simultaneously – another intriguing avenue for further study that is otherwise not possible with existing technologies.

We have added to the Discussion to highlight the potential for broader applications, and look forward to the possibility to explore these directions in future work.

> ***Lines 386–396***
> *"In future applications involving stem cell differentiation or long-term culture, additional factors such as cellular migration, barcode stability, and dynamic gene expression changes would need to be considered; in such cases, stable genetic barcoding or chemically modified SBOs may be more suitable. However, slower-dividing cells, or studies involving terminal differentiation in which cells stop dividing, may support longer-term spatial barcoding in spheroids or developing organoids worthy of further exploration. While over long-term studies, migration of cells within a spheroid could result in mixing of spatially tagged cell populations, our use of fluorescent-tagged SBOs would allow any such migration of barcoded cell subpopulations to be visualised, while also supporting single-cell transcriptomic analysis. Thus, migration and differentiation could potentially be monitored simultaneously – another intriguing avenue for further study that is otherwise not possible with existing technologies."*
> ***Supplementary Figure S2, page S5***
> *New figure inserted with timelapse showing SBO inheritance and quantification by qPCR over time, and calculation of SBO half-life.*

Reviewer #2

Edits to the manuscript are highlighted in yellow.

1. SBO degradation and diffusion: The study mentions the potential degradation of SBOs over time, which could compromise the accuracy of spatial encoding and demultiplexing in longer experiments. However, I'm not entirely convicted by the discussion. For example, inner, middle degradation effects are heavily biased by cell death and stress response and I'm not entirely sure this interpretation only comes from degradation. None is discussed about potential barcode diffusion (for example, double positivity). These two aspects need to be quantitatively addressed by additional set of experiments.

- ✓ It is a valid point that cell death and stress, not just nuclease-mediated degradation, could lead to barcode loss. We have amended this interpretation in the manuscript:

> ***Lines 342–343***
> *"However, we cannot exclude the possibility that cell death or stress in these layers may have also been a factor."*

- ✓ We have quantified SBOs in cells using qPCR (see Reviewer 1, point 2(i)). As expected, we find that the number of SBOs per cell decreases over time, with a half-life of 28 ± 4 h, which is the same as the population doubling time of the HeLa cells. Therefore we conclude that the principal determinant of SBO longevity is cell division, which partitions SBOs between daughter cells.

> ***Supplementary Figure S2, page S5***
> *New figure inserted with timelapse showing SBO inheritance and quantification by qPCR over time, and calculation of SBO half-life.*

- ✓ We did not find evidence of barcode cross-contamination between layers: separation of fluorophore-tagged populations could still be separated by flow cytometry (Supplementary Fig. S2), and demultiplexing with D-score showed >80% unambiguous assignment in three-layer spheroids. This represents a substantial improvement over surface-based barcoding strategies using lipids or antibodies, in which barcode exchange leads to loss of data (see doi:10.1101/2023.08.21.554220v1).

> ***Lines 331–335***
> *"The labelled cell populations could be resolved by flow cytometry (Supplementary Fig. S3), and D-score demultiplexing assigned over 80% of cells unambiguously in three-layer spheroids, suggesting that intracellular delivery of SBOs by scTECH-seq results in minimal barcode diffusion between layers that is often observed using current lipid- or antibody-tagging technologies."*

2. Potential bias in cell types: The use of transformed cancer cells as a model system may not fully capture the heterogeneity of other cell types or tissues, potentially limiting the generalizability of the findings. Particularly this method is highly complex and not entirely clear is applicable when it comes to primary cells, stem cell-derived cell or non-epithelial lineages are included. A lack of experimental designs aimed at diverse applications such as comparative and co-culture models using various cancer cell lines, drug responses and toxicity fields using hepatic spheroid made it difficult to evaluate the advancements and potential of the scTECH-seq method appropriately.

✓ We appreciate the reviewer's keen interest in broader applications of our method.

   i. We have previously applied scTECH-seq to HMLE and mesHMLE cells to study epithelial-to-mesenchymal transition (doi:10.1101/2023.08.21.554220v1), and although this study was 2D-focused, it shows the applicability of our method to other cell types and to study cellular transitions on a temporal scale, highlighting the versatility of this technology.

   ii. We agree that testing scTECH-seq across diverse models including primary cells, co-cultures, and non-epithelial lineages would certainly further demonstrate its generalisability (please also refer to our response to Reviewer 1, point 2). We look forward to the possibility to complete these studies in future now that we have established proof-of-principle.

We have acknowledged the above points through the following amendment:

> ***Lines 381–386***
> *"While we validated spatially-resolved scTECH-seq here using HeLa spheroids, future work could explore applications in primary cells, co-culture systems, or organotypic models such as hepatic or neural spheroids to extend its utility in drug screening, toxicity testing, and developmental biology. We have previously applied scTECH-seq to HMLE and mesHMLE cells to study epithelial-to-mesenchymal transition in 2D cultures, which shows the applicability of our method to other cell types and cellular transitions, highlighting the versatility of this technology."*

3. Limited drug studies: Abstract and title is very bold and sound in characterizing spatially heterogeneous responses to drugs, however, the paper ended up only focusing on irinotecan, a single chemotherapeutic agent. As noted, the most of the biological insights are confirmatory and not requires spatial analysis. Broader studies involving multiple drugs would provide a more comprehensive understanding of the method's utility.

✓ We highlight that our experiments indicate that while cells in the spheroid periphery closely resemble expression profiles observed in 2D cultures, the cells in the middle and interior did not. This is consistent with MALDI-MSI results, where irinotecan distribution in spheroids showed drug accumulating in the core after 24 h, while the active metabolite SN-38 was more abundant in the outer rim and intermediate regions where cells were proliferating (doi:10.1007/s13361-014-1071-0). This supports our transcriptomic findings showing spatial differences in drug response. We therefore find that the spatial position of a cell within a spheroid not only affects its baseline gene expression profile (Fig. 2g) but also affects its response to drug treatment (Fig. 4f, 5a, 5b), in parallel to observations within the tumour microenvironment. This finding represents an advancement in understanding of drug activity in 3D and a new method for exploring the same, rather than simply confirmatory results.

✓ We agree that broader drug testing would strengthen the general applicability. In this proof-of-principle study, we chose irinotecan as a representative chemotherapeutic to test the platform's ability to resolve spatially heterogeneous gene expression. We have added to the Discussion, indicating that future studies could explore other drugs and drug classes to fully leverage the advantages of our method:

> ***Line 396–401***
> *"Future work could expand the drug panel to include compounds with diverse mechanisms of action to further demonstrate the utility of scTECH-seq in profiling spatially heterogeneous drug responses. We envisage that further correlative analysis between 2D and 3D drug response will be helpful in establishing expected gene expression profiles in 3D, and explaining heterogeneous activity and outcomes in response to drug treatment."*

4. Spatial validation: Identified spatially biased genes need a in situ RNA staining to ascertain their discoveries including drug induced spatially heterogenous responsive genes: PARP14, IFITM2 and BCAP31.

   ✓ In the present study, we correlated our expression data with known gene expression signatures (Fig. 5b), which confirms the observed profiles are aligned with the expected drug effects on a genome-wide scale, as opposed to the interrogation of individual genes.

   ✓ We appreciate the reviewer's suggestion regarding spatial validation using *in situ* RNA staining. While we agree that RNA-level validation can provide additional insight, our primary aim in this study was to demonstrate the feasibility and analytical framework for spatial transcriptomics in drug-treated cell cultures rather than to establish definitive spatial heterogeneity for specific genes. To complement the transcriptomic findings, we assessed protein expression for IFITM2 and BCAP31 using immunostaining in three-layer spheroids treated with 50 μM irinotecan for 24 h (the same conditions as in the rest of the paper). Staining for the labelled genes of interest are shown below in green (scale bars = 200 μm). It is also apparent in these images that the SN-38 abundance varies from cell to cell, but whether this is due to loss of the drug post-fixation is not clear.

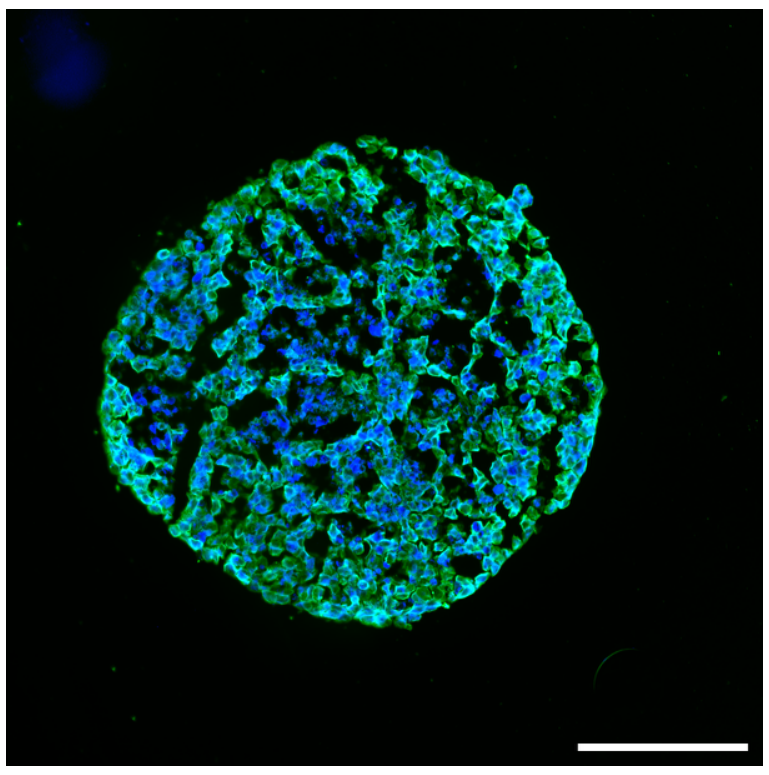

BCAP31

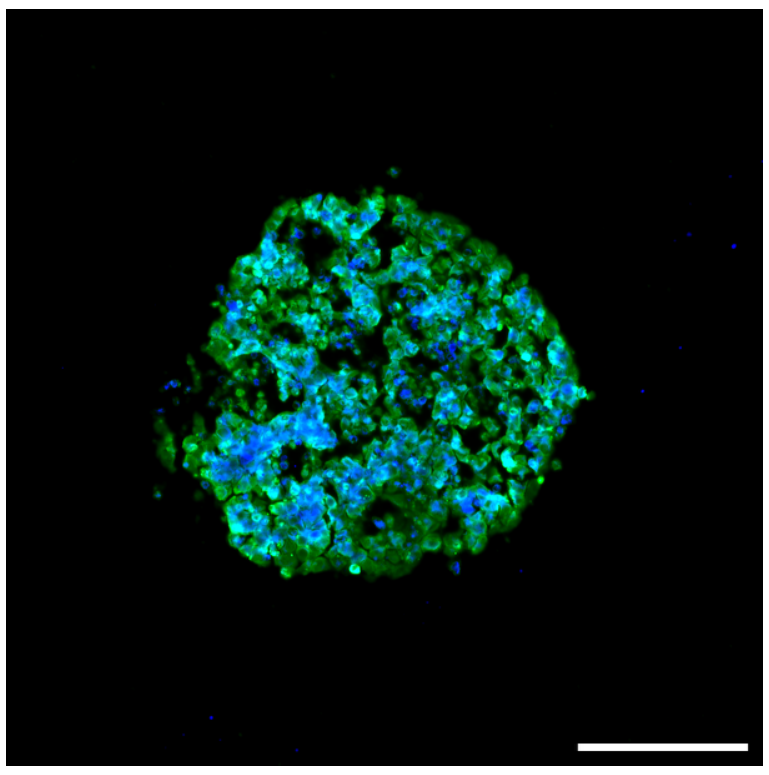

IFITM2

This suggests that any spatial transcriptomic variation may not translate into substantial protein-level heterogeneity under the conditions tested since mRNA and protein levels don't always correlate temporally and post-transcriptional regulation may buffer transcriptional changes. Given that the cell line used is a simplified model and not intended to fully recapitulate tumour complexity, we believe the current validation is sufficient to support the methodological focus of the manuscript.

5. Extensive rewrite: The readers will get confused about the resolution issues by comparing low resolution approach because this paper didn't precisely address resolution problem and no comparable data is presented. The important comparator will be to discuss weakness droplet-sequencing based statistical spatial reconstruction methodologies and advantage of the authors' method.

   ✓ We acknowledge the potential for confusion here and thank the reviewer for alerting us to this issue – please also see our responses to points 6 and 7 where we have addressed this.

   ✓ Statistical reconstruction in our novel architectures composed of a single cell type would be impossible at present, because this technique infers cell position based on similarity in gene expression, which has not yet been experimentally validated. We cannot assess whether there are spatial differences in gene expression if our experiment begins from an assumption that this is true. However, this could certainly be of value in future. We have added the following rationalisation to the manuscript:

> ***Lines 359–369***
> *"An alternative to physical spatial transcriptomics platforms are computational 'statistical reconstruction' methods that infer spatial position from droplet-based scRNA-seq data, such as novoSpaRc, SpaOTsc, and Tangram. These approaches rely on matching gene expression profiles to a spatial reference atlas, or to each other, to reconstruct a pseudo-spatial map. While these techniques are powerful when high-quality spatial reference data are available, their accuracy can be limited in novel systems, cultures consisting of a single cell type, or engineered cultures lacking a priori spatial maps, as is the case here. In contrast, scTECH-seq provides a*

> *direct physical encoding of spatial origin, enabling spatially resolved single-cell transcriptomics in de novo 3D architectures without reliance on external spatial references. There is scope to incorporate statistical reconstruction methods in future, provided that the spatial fidelity of such techniques can be experimentally confirmed."*

✓ We removed the reference to resolution at line 51, and instead mentioned the technical difficulty in using sectioned spheroids with existing spatial transcriptomics platforms, as highlighted in recent work (doi:10.1038/s41598-024-83989-x):

> ***Lines 52–53***
> *"In the context of 3D culture models, which are typically only a few hundred microns in size, existing spatial analysis platforms can be technically challenging to implement."*

Other more specific comments:

6. Line 43-48: Besides the technology platform described by the authors, methods such as Stereo-seq (PMID: 37337030) and Seq-Scope (PMID: 34115981) are reported to achieve spatial resolution at the nanometer scale (~500 nm). Additionally, algorithms that integrate scRNA-seq and Spatial transcriptome data to deconvolute at the single-cell level have been reported (PMID: 38040768). The authors should also discuss these competitive technologies that address the issue of resolution and argue the advantages of scTECH-seq.

✓ We identify the following advantages of our method:

i. Stereo-seq and Seq-Scope use spatially patterned capture chips. Spheroids are much smaller (~400 μm) than tissue sections. Positioning multiple spheroids within a single plane for sectioning, while also maximising the area used on the chip, is technically difficult (doi:10.1038/s41598-024-83989-x).

ii. Using scTECH-seq, we can combine samples using unique barcodes as we did in this study and our EMT study. This allows us to increase the scale and throughput and reduce cost, which would otherwise rapidly become cost-prohibitive for screening many conditions and correlating 2D and 3D expression profiles.

iii. Stereo-seq and Seq-Scope are based on 3' mRNA capture, whereas scTECH-seq allows multiplexing for 5' analysis, which offers better exonic read capture and long-read sequencing potential.

iv. The algorithms that deconvolute at the single-cell level (*e.g.,* novoSpaRc, SpaOTsc, Tangram, *etc.*) are based on having different cell types within the spatial data. The underlying gene expression profile in these different cell types is used to infer cell types within mixed transcriptomic spots. In our case, cells within the spheroid are from the same cell line, so it would not be possible to deconvolve spatial data on this basis. Please also see our response to point 5 above.

✓ We emphasise that scTECH-seq addresses spatial resolution through explicit barcoding and sample multiplexing at the single-cell level within complex 3D geometries. In contrast to approaches such as Stereo-seq or Seq-Scope, which are optimised for thin tissue slices or 2D sections, our method is specifically engineered for spatial analysis within the full 3D culture, where penetration, diffusion, and mechanical microenvironments play critical roles. Thus, while Stereo-seq and Seq-Scope provide impressive spatial resolution in planar tissues, scTECH-seq is uniquely suited for investigating spatial transcriptional dynamics in complex, multilayered 3D cell culture models, making it an ideal platform for spatial drug response profiling.

> ***Lines 322–329***
> *"scTECH-seq offers a number of advantages over existing sequencing methods, including compatibility with 5' gene expression profiling, which offers better exonic read capture and long-read sequencing potential, and highly uniform barcode distribution that improves demultiplexing efficiency. Furthermore, the departure from chip-based 3' mRNA capture allows us to multiplex samples to reduce cost, also facilitating experiments on a larger scale or with greater throughput in future. While existing spatial transcriptomic platforms can achieve impressive resolution in planar tissues, scTECH-seq is uniquely suited for investigating transcriptional dynamics in complex, multilayered 3D cell culture models."*

7. Line 136-138: The spatially defined gene expression patterns are only based on the macro expression profiles of artificially layered spaces. Is the spatial resolution really high when considering single-cell level spatial decomposition? Can we identify subpopulations that form within or move out of the coded spaces?

    - ✓ We identified subpopulations within the individual layers based on UMAP clustering of gene expression profiles (Fig. 4a-c). Therefore, in future work using co-cultures or more complex cell models, we can indeed infer that different cell types or subpopulations could also be readily identified within layers.
    - ✓ Single-cell level spatial decomposition techniques may prove beneficial in future, and we acknowledge that current 'resolution' is indeed limited to finding identified cells somewhere in the barcoded spaces. However, our approach could still provide sequencing data at the individual cell level, whereas the 'resolution' achievable with spatial transcriptomics or decomposition would be based on aggregate expression in groups of cells.
    - ✓ Please see Reviewer 1, point 2(ii) and Reviewer 5, point 1(ii) for further discussion of cellular migration outside the barcoded spaces.

    > ***Lines 232–235***
    > *"Within the three barcoded layers of these spheroids, we can identify different cell populations based on UMAP clustering of gene expression profiles, suggesting that in future work using co-cultures or more complex cell models, different cell types or subpopulations could also be readily identified within spatially barcoded regions."*

8. Line 182-184: Is it possible to quantitatively compare the relationship between the layers in the spheroid by overlapping the Fucci fluorescence signal (mVenus-hGem) and the SN-38 signal? The correlation between irinotecan metabolism and cell cycle changes per layer should be mentioned along with the deconvolution of scRNA-seq data (Figure 4d).

    - ✓ Within our flow cytometry data (line 183), we also collected SN-38 signal in addition to Fucci expression on a per-cell basis which is arguably more representative than individual sections. Within the spheroid flow cytometry data, we divided the irinotecan-treated spheroid cell population into cells containing high and low concentrations of SN-38. The higher the concentration of SN-38, the greater the proportion of green-fluorescent cells (indicating S phase arrest, as expected). We have added this as Fig. 3g-i.

    > ***Lines 208–211:***
    > *"Further stratification of irinotecan-treated cells based on SN-38 fluorescence intensity (Fig. 3g) revealed that the global increase in S-phase population was even more pronounced in SN-38^{high} cells compared to SN-38^{low} cells, confirming that the extent of cell cycle arrest correlated with effective drug concentration (Fig. 3h–i)."*

    - ✓ We expect that it would indeed be possible to correlate Fucci with SN-38 by imaging individual sections, noting that many spheroid sections would likely need to be analysed for sufficient statistical coverage. With the existing experimental design, we could not use Fucci simultaneously with SBO-fluorophore labelling to identify spheroid layers due to spectral overlap. This could be attempted in future using multi-colour or spectral imaging to visualise SN-38, Fucci markers (×2), and fluorescent SBOs (×2–3) to identify spheroid layers and correlate layers to Fucci data simultaneously.
    - ✓ Please note we have corrected details of the fluorescent protein fusions present in Fucci(CA)5: these should be AzaleaB5–Cdt1 and h2-3–Gem1 rather than mCherry and mVenus, respectively.

    > ***Lines 181–182:***
    > *"Representative fluorescence images of Fucci spheroid sections indicate more cells in S phase with cells expressing AzaleaB5–hCdt1 (red), h2-3–hGem (green) or both proteins (yellow). AzaleaB5–hCdt1 fluorescence is indicative of G1 phase, h2-3–hGem fluorescence is indicative of S phase, and cells expressing both markers indicate G2/M phase."*

9. Line 250-255: The differences in drug response correlation between 3D spheroid and 2D monolayer cultures could potentially be due to technical issues. At least, data in 3D spheroids should be validated against camptothecin or some topoisomerase II inhibitors.

- ✓ In the present manuscript, we validate that gene expression signatures reflect what is expected for the drug we tested, and not with unrelated anticancer compounds to provide confidence that identified DEGs align with the effects of irinotecan. We appreciate the reviewer's enthusiasm for the potential of our technique but respectfully note that correlation of 2D and spatial 3D gene expression signatures in response to drug treatment would be a substantial study in its own right, dependent on spheroid size, cell type, drug identity, and drug concentration. While certainly worthwhile, the number of variables here could be explored in a separate study now that proof-of-principle for our method has been established. We include the following statement in the manuscript to acknowledge this is a fledgling approach that may benefit from further validation:

> ***Lines 291–295***
> *"With this proof-of-principle in hand, it would be invaluable to conduct more comprehensive studies with more drugs and drug classes, as well as spheroids of different cell composition and size, to further validate and compare gene expression profiles observed in 2D and 3D cultures, with a view toward establishing 3D spatiotemporal drug atlases."*

10. Lines 268-272: Although the data suggesting uniform elasticity in multilayer spheroids as opposed to monolayer spheroids present an interesting insight, making a connection between these mechanobiological characteristics and single-cell gene expression profiles may be somewhat of a leap. To reiterate, are the interactions between cells uniformly distributed across layers in such structures? Particularly, the expression and pairing pattern of mechanosensing receptors, adhesion molecules and inflammatory/fibrosis responsive genes would be informative. Superimposing spatial information about the structure with intercellular relationship information could enhance the significance of conducting spatial analysis at the cellular layer level.

- ✓ Our observation of homogeneous elasticity distribution and mechanical continuity across spheroid layers supports the expectation that drug penetration differences are minimal, reinforcing our hypothesis that observed gene expression changes are more likely linked to metabolic rather than physical factors. We agree that a direct correlation between mechano-microscopy results and single-cell gene expression profiles would be interesting, though we have avoided making such conclusions in this work.

- ✓ As suggested, we examined the spatial expression of established mechanosensory receptors, adhesion molecules, and fibrotic markers within control spheroids. This included collagens, fibronectin, cadherins, integrins, syndecans, actins, tubulins, myosins, matrix metalloproteinases, TIMPs, and other relevant markers. *ACTG1* was the only gene showing statistically significant spatial variation ($p_{adj} \leq 0.05$), with higher expression in the core compared to the periphery. No other markers exhibited spatial differences, suggesting limited mechanosensory or adhesion-related heterogeneity in HeLa spheroids under these conditions.
  List of genes tested: *COL1A1, COL1A2, COL3A1, FN1, FMOD, POSTN, VCAM1, CCL2, SIRT1, KLF5, PPARG, AKT1, SHH, SMAD7, TGFB1, CTNNB1, MMP2, ITGB1, PLAUR, MMP14, NOX4, CADM1, CD2, CD226, CD274, CD276, CD28, CD34, CD4, CD40, CD58, CD6, CD80, CD8A, CD8B, CD99, CDH1, CDH2, CDH3, CDH4, CDH5, CDH15, ICAM1, ICAM2, ICAM3, ITGA4, ITGA6, ITGA8, ITGA9, ITGAL, ITGAM, ITGAV, ITGB2, ITGB7, ITGB8, SDC1, SDC2, SDC3, SDC4, CITED2, ACTA1, ACTA2, ACTB, ACTBL2, ACTC1, ACTG1, ACTG2, TUBA1A, TUBA1B, TUBA1C, TUBB, TUBB1, TUBB2A, TUBB2B, TUBB3, TUBB4A, TUBB4B, TUBB6, TUBB8, TUBD1, TUBE1, TUBG1, TUBG2, MYO1A, MYO1B, MYO1C, MYO1D, MYO1E, MYO1F, MYO1G, MYO1H, MYO3A, MYO3B, MYO5A, MYO5B, MYO5C, MYO6, MYO7A, MYO7B, MYO9A, MYO9B, MYO10, MYO15A, MMP1, MMP7, MMP8, MMP9, MMP10, MMP11, MMP12, MMP13, MMP15, MMP16, MMP17, TIMP1, TIMP2, TIMP3, TIMP4, TNC.*

- ✓ As noted in the Discussion, conclusive correlation between mechanobiological characteristics and 3D elasticity distribution would require targeted studies in more physiologically relevant models. Nonetheless, our work demonstrates that mechano-microscopy – a novel 3D elastography technique (doi:10.1063/5.0213077) – can be integrated with spatial transcriptomics, enabling future correlative analyses of mechanical properties and gene expression in complex structures.

> ***Lines 307–312***
> *"We profiled a comprehensive set of mechanosensory receptors, adhesion molecules, and fibrotic markers within control spheroids (Supplementary Table S12), but except for ACTG1, which showed significantly higher expression in the spheroid interior compared to the periphery ($p_{adj} \leq 0.05$), no other genes exhibited spatial variation. This finding suggests minimal mechanosensory or adhesion-related heterogeneity in HeLa spheroids, consistent with the uniform elasticity observed by mechano-microscopy."*

Reviewer #5

Edits to the manuscript are highlighted in pink.

1. The spheroid was generated by growing spheroid one layer every 24 hours with pre-barcoded cells, which is not the common protocol for spheroid formation. People would not be able to analyze spatial positioning difference if they do not create spheroid layer-by-layer. What is the advantage of the layered spheroid?

   ✓ Here, the advantage of the layered structure is that it allows quantification of gene expression within different subpopulations of cells based on their 3D position. Using non-spatial scRNA-seq technology, we would not be able to spatially distinguish cells as they all have the same underlying genotype. However, our approach is very flexible and is not limited to layered spheroids.

   i. Cultures could be assembled to form other architectures that mimic cellular physiology, to assess spatial differences in gene expression between cells of the same type.

   ii. Different subtypes or distinct phenotypes of the same cell line could be barcoded to explore what happens in a 3D context over time, such as quantifying migration and differentiation simultaneously (see our response to Reviewer 1, point 2).

   iii. We speculate that it may be possible to barcode cells once spheroids are formed, and relate the number of SBOs per cell position in a similar way to SEEP-seq (see point 4), but this would require substantial further optimisation.

   > ***Lines 81–84***
   > *"The advantage of a layer-by-layer spheroid is that it allows for engineering of a 3D culture with a controlled spatial organisation of barcoded cells; a randomised spheroid would contain different barcoded cells uniformly distributed throughout, which would not allow analysis of different spatial regions."*

2. The 2-layer spheroid is less than 150 μm while the 3-layer spheroid is approximately 400 μm. Large spheroids have been reported to have ki-67 positive signals restricted to the outmost cell layers. The ki67 staining for the 3-layer spheroid exhibits homogenous distribution, which is not expected. Evidence should be provided to determine whether it is due to the spheroid size or to the layer-by-layer operation.

   ✓ We apologise for our oversight; the scale bar in Fig. 1g was stated as being 100 μm but is 200 μm. All scale bars shown on spheroid section images are 200 μm, and thus all spheroids are roughly equal in size. Note that spheroids may appear slightly different in size depending on the sectioning depth, though we have tried to section near the middle of the spheroid wherever possible.

   > ***Lines 94,137***
   > *We have clarified that scale bars are 200 μm.*

   ✓ We have performed the Ki-67 staining in single-layer spheroids constructed in a single layer from either 10,000 or 20,000 cells, and in three-layer spheroids (2000 + 3000 + 5000 = 10,000 cells), as per the following images (scale bars = 200 μm):

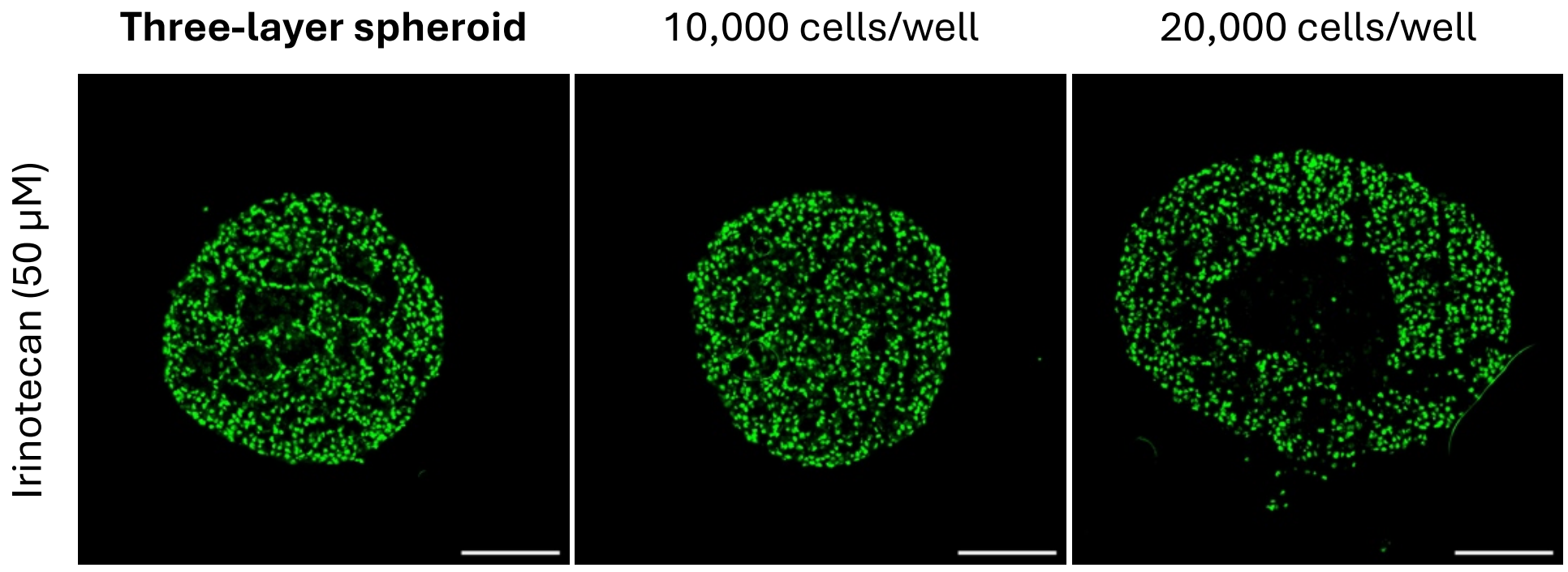

Treatment with irinotecan indeed suppresses proliferation in the interior part of the spheroid, but only in the largest spheroids consisting of 20,000 cells (> 500 µm) formed in a single step. Spheroids consisting of 10,000 cells, whether formed in one step or layer by layer, show a uniform distribution of proliferating cells. Therefore, we can answer the Reviewer's question and say the homogeneous distribution is due to the spheroid size. Note that in the three-layer treated spheroids as used in our study (~ 400 µm) we can perhaps see the beginnings of spatial variation.

> ***Fig. 3***
> *We have improved the Ki-67 staining results and updated these as Fig. 3d.*

3. Seeding each layer requires a 24-hour interval. How many layers can one do at most? Will the interior cells die if the culturing is too long?

✓ Yes, this is quite possible. We tested up to 72–96 h in three-layer spheroids for the following reasons:

i. We felt confident that three layers would be sufficient to establish trends in gene expression;
ii. We considered the number of unique fluorophores we could image based on microscope hardware;
iii. Spheroid growth, treatment, and dissociation could be completed in one week; and
iv. Based on expert advice from our sequencing partner Genomics WA, multiplexing two, three-layer spheroids for a total of 6 samples across two sequencing chip channels would ensure sufficient cells to analyse gene expression clusters within each multiplexed layer/treatment.

✓ We could certainly explore more rapid seeding of cell layers, or seeding of additional layers in future, noting the following:

i. We observed a decline in the ability to assign cells using D-score, particularly to the interior population. At this stage, we cannot definitively say whether this is due to cell death, stress, proliferation, or degradation. This is a risk that requires further investigation, but is also likely cell-type specific. See also our response to Reviewer 1, point 2(i) for qPCR of SBOs over time.
ii. The cell type and density within the spheroid would certainly influence cell death, proliferation, nutrient supply and waste clearance. This is true of any spheroid culture and we recommend that cell viability be confirmed prior to attempting our technique.

> ***Lines 347–350***
> *"Furthermore, additional layers, greater numbers or multiplexing of fluorescent tags, or more rapid addition of layers to the growing spheroid to facilitate a more fine-grained analysis of spatial gene expression. It would be prudent to confirm cell viability when extending the culture to a greater number of layers or over a longer time period."*

4. The reference #36 used dye penetration to identify different layers of spheroids. It is recommended to compare your method with this method. The drug response study can also be done using this method and compared with the current study.

✓ We make the following points, and clarify in the manuscript:

i. SEEP-seq is inherently limited to situations in which there is a difference in dye permeation, but our technique could allow barcoding and separation of different cell types, or even different phenotypes within one cell line, within 3D architectures, which is not possible using SEEP-seq.
ii. SEEP-seq is specific to diffusion length differences and requires extensive optimisation for every cell type and spheroid size to obtain a dye gradient, which is more labour-intensive than our method.
iii. SEEP-seq uses cell binning by FACS, requiring additional time for cell sorting that further impacts gene expression as cells are exposed to shear and pressure changes.
iv. Dye penetration may begin to differ with certain treatments, which can alter the spatial assignment of cells. Our technique assigns cells with greater certainty, as cell populations for analysis are determined prior to treatment.

> ***Line 355–359***
> *"SEEP-seq provides information in relation to drug diffusivity, whereas our approach is more application-agnostic. We see complementary potential for SEEP-seq and our technique, which is not limited to diffusion, and could be used to profile aspects of 3D culture, including proliferation or epithelial–mesenchymal transition, which may not be directly related to diffusivity, especially in more complex architectures."*

5. Please provide evidence that cells grow after seeding and if so, the newly divided cells still contain SBOs.

✓ To the first point, our revised Ki-67 staining results (see This Reviewer, point 2), together with the existing Fucci and scRNA-seq cell cycle analysis, demonstrate cells may be found in all phases of the cell cycle (not arrested) and are proliferating after the spheroid is established.

✓ We have added a timelapse experiment as Supplementary Figure S2 to show that SBOs are partitioned in daughter cells post-division. Taken together with qPCR to quantify SBOs within cells over time (see Reviewer 1, point 2(i)), we find that the number of barcodes per cell follows the rate of cell division, suggesting that division is the primary determinant of SBO longevity rather than degradation. We have made two amendments to clarify:

> ***Lines 74–77***
> *"SBOs were inherited by daughter cells following cell division and had an apparent half-life in HeLa cells of 28 ± 4 h (Supplementary Figure S2), which matches the cell population doubling time and is consistent with the number of SBOs per cell halving with each cell division."*
> ***Lines 335–339***
> *"We found evidence of reduced SBO numbers that would likely compromise successful demultiplexing after this timepoint, as revealed by SBO quantification over time (Supplementary Figure S2). The fact that SBO abundance correlates with the cell population doubling time suggests that division is the primary determinant of SBO longevity rather than degradation."*

6. What is the advantage of the dendritic polymer transfection? Can you compare it to the widely used method such as lipid, lipofectamine or nucleofection? How did you determine your transfection conditions with the polymer? There isn't a mature protocol for this kind of transfection.

✓ The benefit of polymer transfection here is endocytosis, resulting in complete cellular internalisation of SBOs. Competing technologies such as CITE-seq employ lipid- or antibody-based anchoring of SBOs to the cell membrane, which is reversible and leads to barcode exchange, meaning cells cannot be unambiguously demultiplexed (see also Reviewer 2, point 1). We have optimised this in our preprint doi:10.1101/2023.08.21.554220, where we found that the estimated rate of recovery of cells using scTECH-seq is >72% when compared to Lipofectamine-based barcoding (~44%). We also observed that cells barcoded via polymer-based delivery had a more uniform, narrow uptake of the SBOs in comparison to Lipofectamine 2000 as assessed by flow cytometry.

✓ For the transfection conditions, we based our method (as described in doi:10.1101/2023.08.21.554220) on that of Shin *et al.* (10.1126/sciadv.aav2249) which is almost identical to our lab's previous publications in DNA delivery, using 1 µg dsDNA per well in a 24-well plate. This scale could be adjusted

up or down to provide stronger barcoding or reduced toxicity, as appropriate. We offer the following clarification for readers:

> ***Lines 319–322***
> *"Polymer transfection by scTECH-seq is advantageous, because it results in complete cellular internalisation of SBOs via endocytosis. Alternative strategies such as CITE-seq employ lipid- or antibody-based anchoring of SBOs to the cell membrane, which is reversible and prone to barcode exchange between cells."*
> ***Lines 72–73***
> *"…using 23 pmol SBOs per 150,000 cells, based on previous reports (10.1126/sciadv.aav2249, 10.1101/2023.08.21.554220)."*

However, the study by Shin *et al.* was conducted using 3'-gene expression and cell recovery rates were not reported, making it difficult to compare the efficiency of this approach.

7. Does the cluster 4 (dead cells) in Figure 2b come from the interior? Additional figures showing the three layers instead of 4 clusters will better illustrate the distribution of the three layers in the 4 clusters.

✓ Cluster 4 is not only from the interior. As shown in Fig. S3 for the two-layer spheroids, the small cluster with very different gene expression is fairly balanced in representation from the interior and periphery. Similar observations can be made for three-layer spheroids: cells in that minor cluster represent 11% of all cells in the interior layer, 12% of all cells in the middle layer, and 6% of all cells in the periphery. We speculate these may be cells that were damaged during spheroid dissociation.

> ***Lines 122–124***
> *"This cluster contained cells from each layer (from 6 to 12% of the cells in each layer, representing 7.0% of all cells) which we speculate comprises cells that were damaged during spheroid dissociation, so they were excluded from further consideration."*

8. The cell cycle category used in figures 2, 3 and 4 are different. It is unclear why the authors included early-S phase in figure 2 how they distinguish the early-S fraction?

✓ In Figure 3f we quantified Fucci markers to determine cell cycle state, whereas in all other figures we used scRNA-seq data. The distinction here is that scRNA-seq analysis assigns cells to G1, S, or G2/M, whereas Fucci(CA)5 identifies the following cell cycle stages according to fluorescence: late S phase (green only), G1 (red only), G2/M (green and red), early S phase (no fluorescence) as per Ando *et al.* (10.1247/csf.23028, Fig. 1b). We have highlighted the reason for the difference in the manuscript:

> ***Lines 206–208***
> *"Here we used Fucci, rather than gene expression profiles, to visualise changes that occurred and confirm the spatial arrangement of cell cycle arrest within spheroids."*

9. There is a group of genes activated in the interior but not in the periphery upon irinotecan treatment (figure 4f, top). It is important to provide explanation for this phenomenon.

✓ Looking at the genes at the top of Fig. 4f, while it is true that there was gradient in expression of these DEGs (higher in the interior, lower in the periphery) in treated spheroids, the same expression gradient was observed in untreated spheroids. The overall expression of these genes was increased in the presence of irinotecan, but with the same spatial pattern. We speculate that this may represent drug-responsive genes that are not influenced by the different metabolic states of interior *vs* peripheral cells found within spheroids.

10. In Supplementary figure S7, it seems that lots of cell died upon irinotecan treatment, even the vehicle results in a significant portion of dead cells. The flow cytometry data is not convincing, and live and dead cells percentage should be indicated in the figure.

✓ We believe that there are two effects here:

i. First, a small amount of cell death probably arises during dissociation of the spheroid,

ii. Second, dead cell populations may have been overestimated as we had to transport dissociated live cell suspensions to the flow cytometry facility at a different campus for analysis, during which further death may have occurred, and

iii. Finally, the flow cytometry gating was conservative and probably overestimates dead cells.

- ✓ Please note that in Fig. 3a the viability was normalised to the vehicle control, leading to an apparent discrepancy with Fig. S8. We have clarified this point in the Fig. 3a caption.

> ***Fig. 3a***
> *The flow cytometry gating has been reassessed, being less conservative, and now shows a trend we consider more fairly represents our experimental findings.*
> ***Supplementary Fig. S8***
> *Flow cytometry plots have been updated with percentages shown.*

11. It'd be interesting to detect and measure irinotecan-induced TOP1 cleavage complexes (TOP1cc) for each spheroid layers as stable TOP1cc induction is the primary mechanism of action of TOP1 inhibitors. Please refer to PMID: 37353483 for TOPcc detection in 3D organoids.

- ✓ We appreciate the reviewer's interest in measuring irinotecan-induced TOP1 cleavage complexes (TOP1cc) across spheroid layers, as this would provide mechanistic insight into drug action. However, implementing this approach in our current workflow would be technically challenging. Our primary aim here was to demonstrate the feasibility of spatial transcriptomics for drug-treated spheroids rather than to exhaustively characterize TOP1cc formation. However, we have noted this as an important future direction in the Discussion:

> ***Lines 401–403***
> *"Additionally, future studies could aim to quantify drug-induced TOP1 cleavage complexes within spheroid layers to directly link spatial transcriptomic profiles with the primary mechanism of action of TOP1 inhibitors, providing a more comprehensive understanding of spatial drug responses."*

12. No scale bar in figure 2a.

- ✓ We have added the missing scale bar (it is identical to that in Fig. 1).

13. Please mark consistently for figures. For example, in Supplementary Figure S9, mark "control" and "drug" the same way as the main figures.

- ✓ We apologise for this oversight and have adjusted figure labels under the plots.

Single-cell RNA sequencing profiles drug activity within spatially engineered 3D cultures

Jessica J. King[1], Alireza Mowla[2,3], Jessica A. Kretzmann[1,4], Nishka Bhalla[1,4], Giselle Sugianto[1], Marck Norret[1], Ulrich D. Kadolsky[5], Munir Iqbal[5], Alka Saxena[5], Sebastian E. Amos[6], Yu Suk Choi[6], Brendan F. Kennedy[2,3,7], K. Swaminathan Iyer[1,4], Nicole M. Smith[1,4]*, and Cameron W. Evans[1,4,8]*

[1] School of Molecular Sciences, The University of Western Australia, Perth WA 6009, Australia.

[2] BRITElab, Harry Perkins Institute of Medical Research, QEII Medical Centre and Centre for Medical Research, The University of Western Australia, Nedlands, Perth WA 6009, Australia.

[3] Department of Electrical, Electronic & Computer Engineering, School of Engineering, The University of Western Australia, Perth, WA 6009, Australia.

[4] ARC Training Centre for Next-Generation Biomedical Analysis, The University of Western Australia, Perth WA 6009, Australia.

[5] Genomics WA, Harry Perkins Institute of Medical Research, Telethon Kids Institute, and The University of Western Australia, Nedlands, Perth WA 6009, Australia.

[6] School of Human Sciences, University of Western Australia, Perth WA 6009, Australia.

[7] Institute of Physics, Faculty of Physics, Astronomy and Informatics, Nicolaus Copernicus University in Toruń, 87-100 Toruń, Poland.

[8] School of Pharmacy and Pharmacology, University of Tasmania, Sandy Bay TAS 7005, Australia.

* Corresponding authors: Cameron W. Evans, cameron.evans@uwa.edu.au; Nicole M. Smith, nicole.smith@uwa.edu.au.

Abstract

Spatial transcriptomic techniques provide a wealth of information useful in guiding drug development, while three-dimensional (3D) cell cultures have demonstrated power in accelerating drug approvals. However, techniques for robust spatial analysis of 3D cultures are limited. Here, we present a transfection-based method for constructing cellular architectures in which DNA barcodes encode the spatial positioning of cells. Our technique facilitates imaging and multiplex single-cell RNA sequencing, providing spatial maps of gene expression and drug response, while correlative imaging with mechano-microscopy captures spatial variations in tissue elasticity. We show that 3D cultures display heterogeneous responses to drugs, which are not only due to diffusion gradients of the drug from periphery to core, but also from differences in metabolism, nutrient supply, and cellular stressors. The ability to create spatially encoded cellular assemblies is a general approach that can resolve spatial variation in gene expression within 3D culture models at the single cell level.

Introduction

Organ-on-a-chip platforms[1] demonstrate the power of three-dimensional (3D) cultures to accelerate animal-free clinical drug approvals. While *in vivo* animal studies are more biologically relevant than two-dimensional (2D) monolayer cell cultures for drug screening[2], animal models can be costly, time-consuming,[3] and often fail to translate to humans[4–6]. As an attractive 3D intermediary between homogeneous 2D cell cultures and highly complex tissue samples, 3D cultures have been successfully implemented in cancer and tumour research, and drug screening applications[7]. Spheroids are readily scalable 3D cell cultures that form sphere-like masses *via* cell-to-cell interactions, which may adopt a structural and spatial complexity similar to that found *in vivo*[8]. 3D cultures can more faithfully represent the heterogeneous cellular responses found in animal models, reflecting gradients in oxygen and nutrient supply, metabolic rate, and cell proliferation.

The ability to engineer 3D cultures with pre-coded spatial information will help to enrich drug screening and gene expression data. Understanding how cell position relates to transcriptomic changes could provide insight into how drug penetration, differences in the cell microenvironment, or cell–cell interactions contribute to drug response or disease progression. Characterising gene expression in 3D requires spatially-resolved single-cell RNA-seq (scRNA-seq) data[9] to capture transcriptomic profiles in the context of cellular organisation[10]. 10X Genomics[11,12] and NanoString Technologies[13] offer spatial analysis platforms, but these methods currently cannot capture the gene expression profiles of individual cells, since the spatial resolution is limited to ~50 µm[13,14]. In the context of 3D culture models, which are typically only a few hundred microns in size, existing spatial analysis platforms can be technically challenging to implement[15]. Furthermore, the low throughput limits analysis of 3D architectures[16]. A platform for the study of gene expression that captures profiles at the single cell level while also maintaining critical spatial information will help to bridge the divide between homogeneous 2D cell culture and animal models for analysing 3D cultures.

Here, we construct multi-layered spheroids in which short barcode oligonucleotides (SBOs) encode spatial information and facilitate sample multiplexing for scRNA-seq, a technique we term single cell transfection-enabled cell hashing (scTECH-seq).[17] Herein, we adopt our scTECH-seq method, using SBO–fluorophore conjugates to enable visualisation of barcoded cells in 3D spheroid cultures, prior to scRNA-seq. We demonstrate the construction of barcoded HeLa spheroids through a layer-by-layer assembly process and spatially profile drug-induced transcriptional responses within 3D cell cultures. The use of fluorescent barcodes also supports correlative imaging analysis using a recently reported mechano-microscopy technique[18] to measure tissue elasticity within 3D cultures.

Results and discussion

scTECH-seq enables visualisation and single-cell sequencing of multi-layered spheroids

scTECH-seq uses SBOs as described by 10X Genomics, which we modified through addition of a fluorophore label at the 5′ end to enable SBO visualisation and quantification in cells (Supplementary Figure S1, Supplementary Table S1). In this scheme, each individual cell is assigned a unique molecular identifier (UMI) during single-cell library preparation via the 10X Genomics Chromium platform, while SBOs serve as feature barcodes that distinguish different samples of cells. HeLa cells were transfected for 4 h with dye-conjugated-SBOs (Fig. 1a) using 23 pmol SBOs per 150,000 cells, based on previous reports[17,19]. SBO uptake was visualised with fluorescence microscopy (Fig. 1b), and flow cytometry quantification of fluorescent SBOs indicated >99.9% labelling efficiency (Fig. 1c). SBOs were inherited by daughter cells following cell division and had an apparent half-life in HeLa cells of 28 ± 4 h (Supplementary Figure S2), which matches the cell population doubling time and is consistent with the number of SBOs per cell halving with each cell division. A comparison of scRNA-seq using fluorophore-modified versus unmodified SBOs showed no reduction in RNA transcripts per cell, gene counts, or SBO numbers per cell (Supplementary Tables S2–S4) when using Cy5-modified SBOs compared to unmodified SBOs (Fig. 1d,e). We used SBO-labelled cells to construct layered spheroids,

in which each layer comprised cells transfected with a unique SBO (Fig. 1f). The advantage of a layer-by-layer spheroid is that it allows for engineering of a 3D culture with a controlled spatial organisation of barcoded cells; a randomised spheroid would contain different barcoded cells uniformly distributed throughout, which would not allow analysis of different spatial regions.

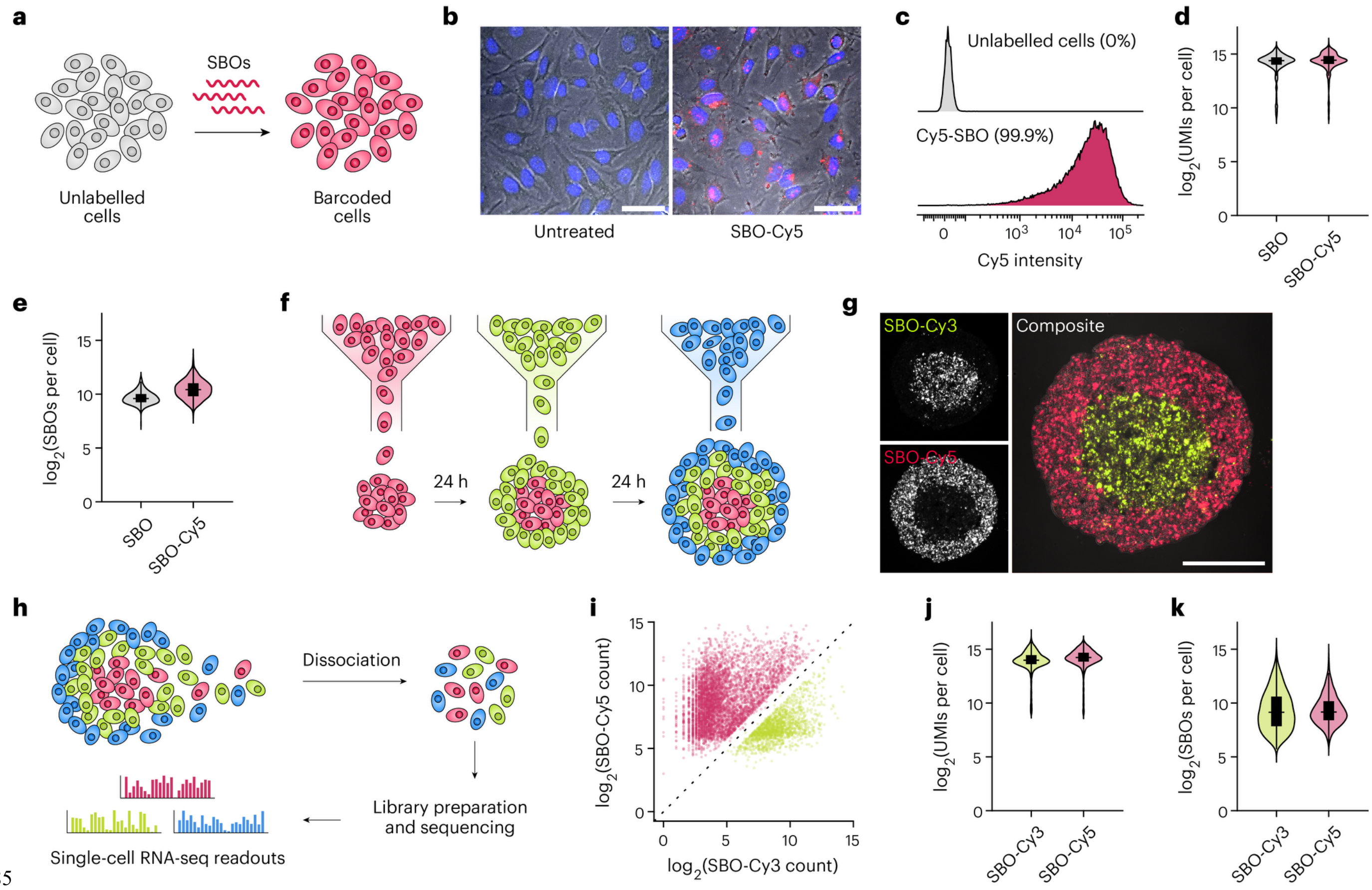

Figure 1. Single-cell transcriptomics of multi-layer spheroids. (a) Cells are transfected with SBOs in culture to produce populations of 'barcoded' cells. **(b)** Fluorescent microscopy shows SBO (red) uptake in HeLa cells. Scale bar 50 µm. **(c)** SBO uptake efficiency was measured using flow cytometry, with >99% cells positive for SBO fluorescence (two biological replicates, $n = 5$). **(d)** Violin plot showing similar number of RNA counts per cell for SBOs with or without Cy5 modification. **(e)** Violin plot shows similar number of SBO counts per cell with or without Cy5 modification. **(f)** Schematic of the experimental approach for barcoding cell populations and growing spheroids using a layered approach. Different colours represent unique SBO sequences, each encoding a spatial region of interest. **(g)** Representative image of spheroid cross-section showing two cell layers, labelled with Cy5- (red) and Cy3- (yellow) conjugated SBOs. Scale bar 200 µm. **(h)** Schematic of sequencing preparation process. Spheroids are dissociated into single cell suspension and undergo encapsulation and reverse transcription using the 10X Chromium Controller. Data is retrieved and analysed using CellRanger and Seurat. **(i)** Cy5- and Cy3- modifications do not interfere with SBO sequencing and deconvolution using D-score. **(j)** Violin plot shows

a similar number of RNA counts per cell for Cy5-SBO and Cy3-SBO. **(k)** Violin plot shows a similar number of SBO counts per cell for Cy5-SBO and Cy3-SBO.

Confocal microscopy of spheroid cryosections enabled visualisation of two distinct barcoded layers (Fig. 1g). Subsequent dissociation of spheroids (Fig. 1h) and quantification of SBOs *via* flow cytometry confirmed that barcodes were not lost or exchanged between individual cells (Supplementary Fig. S3). Two-layer spheroids tagged with Cy3- and Cy5-SBOs were dissociated, pooled, and sequenced, whereby SBOs also facilitated demultiplexing of scRNA-seq data (Fig. 1i). For the two layers, we observed similar numbers of RNA transcripts, genes, and SBO reads per cell (Fig. 1j–k, Supplementary Tables S5–S7), demonstrating capacity to construct and visualise SBO-labelled architectures using unmodified or fluorophore-labelled SBOs without compromising sequencing reads or demultiplexing integrity. Analysis of gene expression in two-layer spheroids can be found in the Supplementary Notes and Supplementary Fig. S4–S6.

Barcoding facilitates examination of spatial transcriptional differences in multi-layer spheroids

Extending the approach described above, we constructed spheroids from three populations of HeLa cells, each of which had been labelled with a unique SBO. Barcoded cell populations were added each day over a 3 d period (Fig. 2a), creating three defined layers. Spheroids were dissociated and sequenced as above. Transcript, gene, and SBO counts are summarised in Supplementary Tables S9–S11. Using the D-score tool for demultiplexing scRNA-seq data[17], 82.5% of cells were unambiguously assigned to one of the three SBOs used, deconvolving cell populations by SBO and hence also by spatial organisation. Cells were clustered into four groups by gene expression during analysis (Fig. 2b). Many of the differentially expressed genes (DEGs) between clusters could be attributed to cell cycle progression, and analysis of the cells in clusters 1–3 confirmed substantial differences in the number of cells in G1, S, and G2/M phase (Fig. 2c). A subpopulation of cells (cluster 4, representing 7.0% of total cells) displayed hallmarks of cell death, including deregulation of housekeeping genes including *ACTB*, *GAPDH*, *HPRT1*, and *EIF* family members. This cluster contained cells from each layer (from 6 to 12%

of the cells in each layer, representing 7.0% of all cells) which may represent cells that were damaged during spheroid dissociation, and these were excluded from further consideration.

Spatial analysis of gene expression revealed 117 DEGs ($|\log_2$ fold change$| > 0.25$, $p_{adj} < 0.05$) between the interior, middle and peripheral layers of three-layer spheroids. Examining cell cycle with respect to each of the three layers within the spheroids rather than cluster showed a similar distribution of cell cycle markers across the layers (Fig. 2d), while further examination of the top DEGs across the three layers revealed markers of growth, metabolism, and waste processing (Fig. 2e). Despite some indicators of cell stress and death within a small subpopulation of cells, the principal transcriptomic differences in multilayer spheroids were the result of growth and cell division. Expression profiles in spheroid interior regions confirmed some enrichment of apoptotic and necrotic pathways (Supplementary Fig. S7).

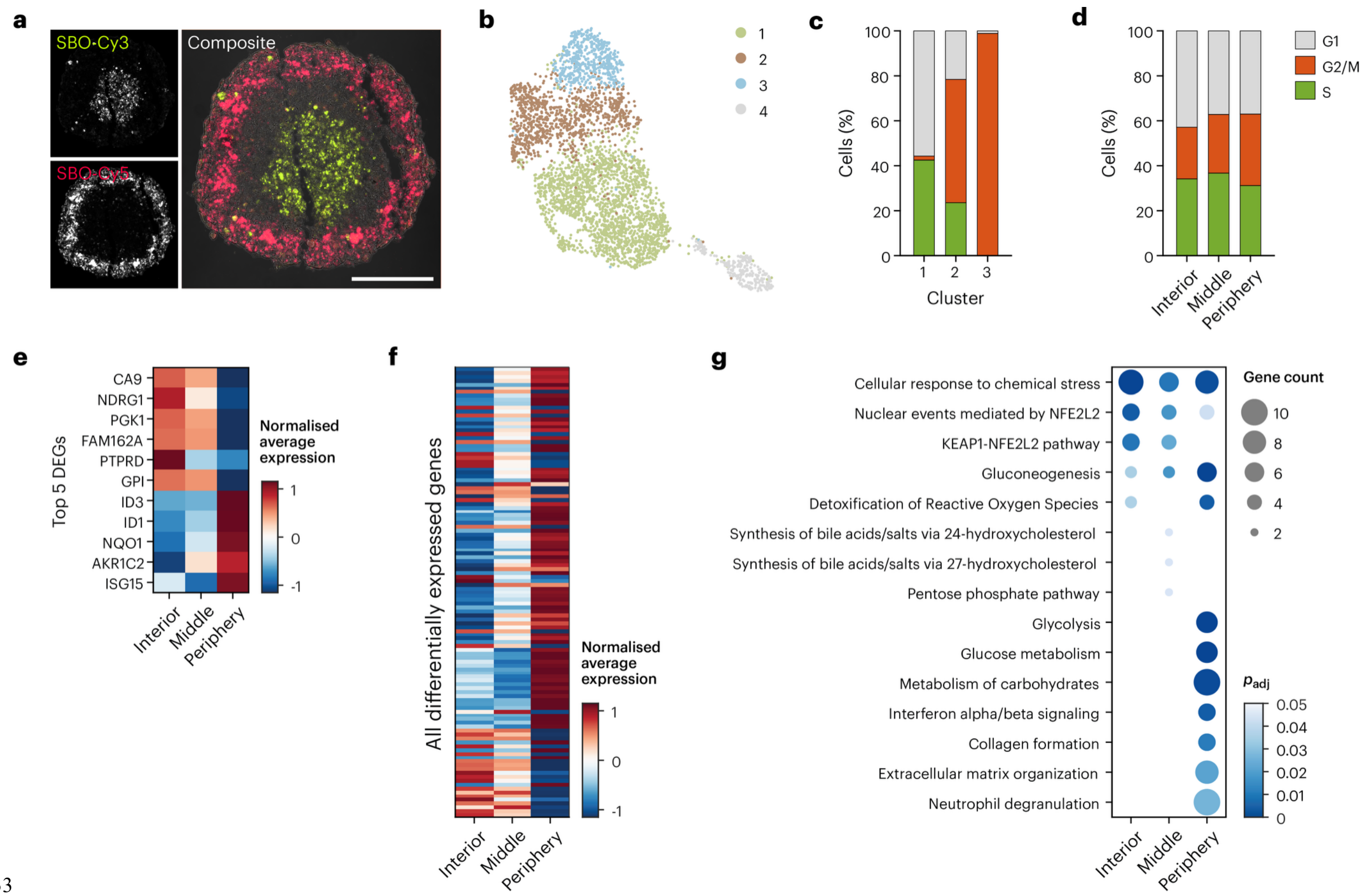

Figure 2. Multi-layer spheroids highlight spatial differences in gene expression related to cell cycle, metabolism, and nutrient supply. (a) Representative image of three-layer spheroid. Cells within the core (first

layer) are labelled with Cy3-SBO (yellow), the middle layer contains cells barcoded with an SBO that was not conjugated to a fluorophore, and cells on the periphery are labelled with Cy5-SBO (red). Scale bar 200 µm. **(b)** UMAP representation of the single cells recovered from three-layer spheroids. Cells are coloured by gene expression cluster. **(c)** Quantification of cell cycle markers within each of the three main clusters identified in (b). Cluster 3 principally contains cells undergoing mitosis (G2/M), while cluster 1 displays virtually no markers of G2/M phase. Thus, the main differences in gene expression across untreated spheroids are simply due to cell cycle. **(d)** Distribution of cell cycle markers according to spheroid layer show that cell cycle progression is relatively consistent between each of the three layers. **(e)** Normalised average expression levels of the top 5 differentially expressed genes (DEGs, $|\log_2 \text{fold change}| > 0.25$, $p_{adj} < 0.05$) across three-layer spheroids. **(f)** Normalised average expression levels for all DEGs ($|\log_2 \text{fold change}| > 0.25$, $p_{adj} < 0.05$) across three-layer spheroids. **(g)** Dot plot showing gene ontology (biological process) pathways enriched within each layer of three-layer spheroids. The dot size indicates the gene counts expressed in each pathway, while colour indicates adjusted *p*-value.

The top 5 DEGs identified in each layer of three-layer spheroids are shown in Fig. 2e. Of these, 8/11 genes were also found to be differentially expressed in two-layer spheroids (Supplementary Table S8). Qualitatively, across all DEGs, the middle layer tended to display expression levels that were intermediate between the interior and periphery (Fig. 2f), supporting our hypothesis that gradients in gene expression patterns could be spatially defined. Pathway analysis using DEGs was conducted (Fig. 2g); KEAP1-NFE2L2 pathways and NFE2L2 nuclear events were upregulated in the interior, consistent with an increase in oxidative stress, while glycolysis, glucose metabolism, and ECM formation and organisation processes were more prevalent in the peripheral layer. These results are consistent with the current understanding of spheroid cultures in which there is a gradient in metabolic rate, nutrient supply, and oxygen dependent on cell depth, and dense cell-to-cell contact and suppressed cell proliferation in the spheroid core[20].

In vitro analysis of irinotecan-treated spheroids

To explore the spatial analysis of gene expression in response to an experimental stimulus, three-layer spheroids were treated with irinotecan. Irinotecan is a prodrug that is used for many cancers[21–24], particularly colon cancer. The primary target of irinotecan is DNA topoisomerase I, which relaxes supercoiled DNA during replication and transcription[25,26]. Carboxylesterases metabolise irinotecan to ethyl-10-hydroxycamptothecin (SN-38)[27,28], which is a fluorescent metabolite with excitation at 360 nm

and emission at 420 nm, making it an ideal candidate to monitor drug penetration of spheroids. SN-38 stabilises topoisomerase I–DNA complexes, resulting in double stranded DNA breaks and activation of apoptotic pathways[26]. Using spheroids with three layers, we tested different concentrations of irinotecan prepared in 0.1% v/v DMSO for 24 h, as well as a 0.1% v/v DMSO control (hereafter referred to as 'vehicle control') and an untreated control (Supplementary Fig. S8). The percentage of live cells was quantified *via* flow cytometry using propidium iodide staining (Fig. 3a). For subsequent studies, 50 µM was selected.

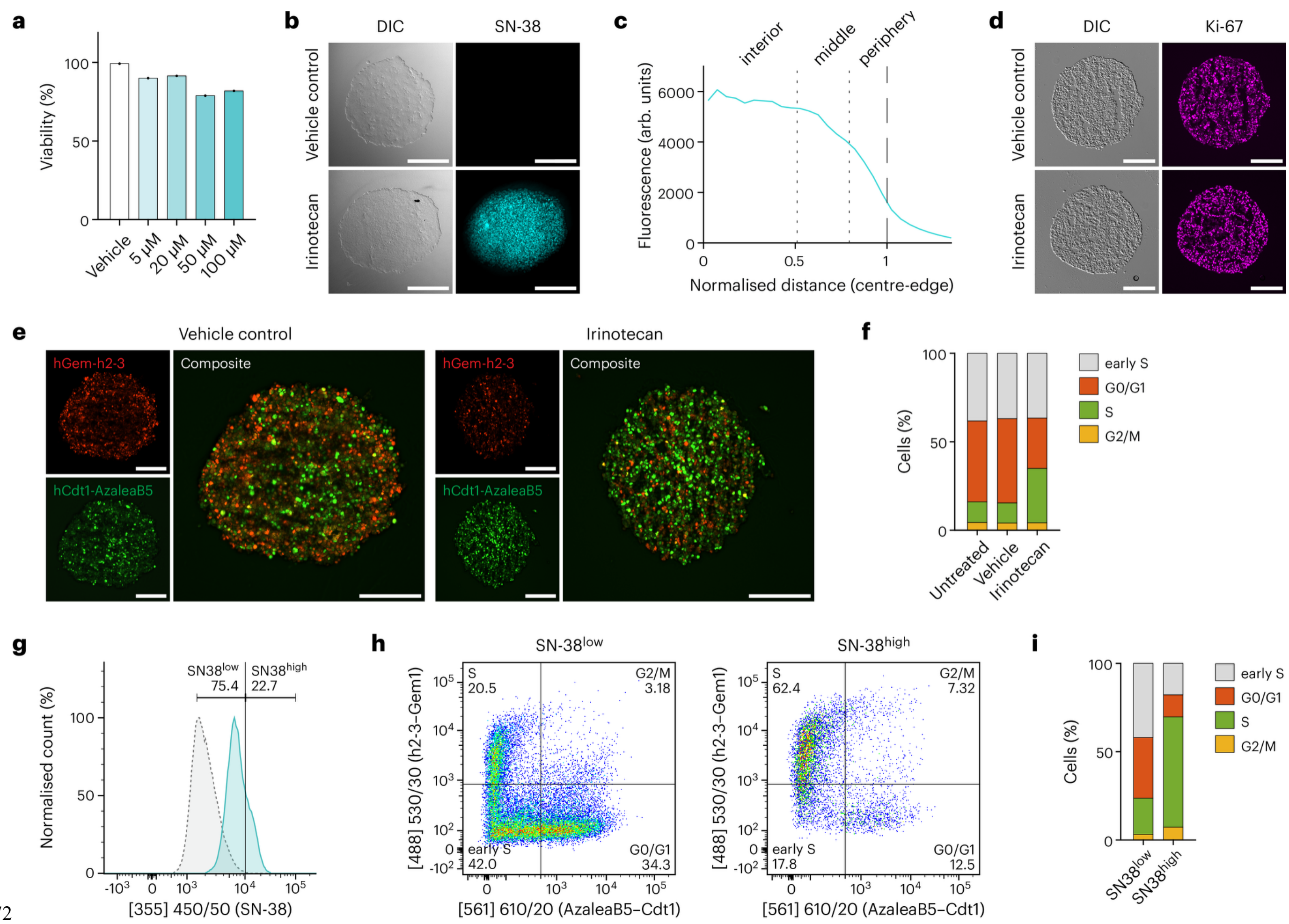

Figure 3. Topoisomerase I inhibition alters cell cycle state in three-layer HeLa spheroids treated with irinotecan for 24 h. (a) Percentage of live cells from dissociated spheroids after 24 h irinotecan treatment using propidium iodide. The percentage of live cells (mean ± standard deviation) was calculated via flow cytometry and normalised to the vehicle control. 0.1% DMSO ('vehicle') is the control. 50,000 single cells were collected per sample. **(b)** Epifluorescence microscopy images of spheroid cryosections in which fluorescence was used for SN-

38 detection. Scale bars 200 µm. **(c)** Quantification of SN-38 fluorescence across the diameter of the spheroid cross-section shown in (b). **(d)** Ki-67 staining of spheroid cryosections (*n* = 4–5 spheroids). Scale bars 200 µm. **(e)** Representative fluorescence images of Fucci spheroid sections show that irinotecan arrests cells in S phase with more cells expressing AzaleaB5–hCdt1 (red), h2-3–hGem (green) or both proteins (yellow). AzaleaB5–hCdt1 fluorescence is indicative of G1 phase, h2-3–hGem fluorescence is indicative of S phase, and cells expressing both markers indicate G2/M phase. Scale bars 200 µm. **(f)** Flow cytometry quantification of dissociated Fucci spheroids show an increase of cells in S phase after irinotecan treatment. Spheroids (n = 8) per condition were dissociated and 50,000 single cells were processed during flow cytometry. Flow cytometry graphs are representative of two biological replicates. **(g)** Analysis of cell cycle arrest as a function of drug concentration. Fucci HeLa spheroids were treated with irinotecan, dissociated, and analysed by flow cytometry. Irinotecan-treated cells (blue shaded curve) were separated into SN-38^{high} and SN-38^{low} populations for analysis. Cells from untreated spheroids are shown as a dotted grey line. >30,000 cells were analysed. **(h)** Scatter plots for the two populations shown in (g) based on expression of each of the Fucci markers. The percentage of cells in each quadrant is shown. **(i)** Quantification of cell cycle by Fucci in SN-38^{high} and SN-38^{low} cells as shown in (h) confirms that the proportion of cells arrested in S phase correlates with the effective SN-38 concentration.

Imaging of SN-38 revealed a relatively homogenous distribution throughout all spheroid layers (Fig. 3b), with a reduction in fluorescence intensity towards the periphery (Fig. 3c), however we speculate that this may have arisen due to drug diffusion and loss during sample processing. Ki-67 was used to evaluate proliferation in spheroids before (vehicle control) and after irinotecan treatment. Spheroids possessing an apoptotic or necrotic core were expected to have fewer Ki-67^+ cells, but immunofluorescence of spheroid sections indicated that Ki-67^+ cells were distributed homogeneously (Fig. 3d). Irinotecan treatment did not significantly reduce the number of Ki-67^+ cells in spheroids compared to vehicle control.

Since both irinotecan and its active metabolite SN-38 stabilise topoisomerase I–DNA complexes and induce DNA damage, we assessed cell cycle progression as an indicator of irinotecan activity in three-layer spheroids using the fluorescent, ubiquitination-based cell cycle indicator Fucci(CA)5 system[29,30]. Spheroids composed of Fucci-engineered HeLa cells revealed an increase in h2-3-hGem expression when treated with 50 µM irinotecan (Fig. 3e), which was quantified by flow cytometry, indicative of cell cycle arrest either prior to completion of S phase or at the G2 checkpoint (Fig. 3f). Here we used Fucci, rather than gene expression profiles, to visualise changes that occurred and confirm the spatial

arrangement of cell cycle arrest within spheroids. Further stratification of irinotecan-treated cells based on SN-38 fluorescence intensity (Fig. 3g) revealed that the global increase in S-phase population was even more pronounced in SN-38^{high} cells compared to SN-38^{low} cells, confirming that the extent of cell cycle arrest correlated with effective drug concentration (Fig. 3h–i).

scRNA-seq resolves heterogeneity within spheroid layers

We sequenced HeLa spheroids composed of three uniquely barcoded layers to study the spatial differences in gene expression triggered by irinotecan treatment. Vehicle control and spheroids treated with 50 μM irinotecan for 24 h were dissociated and libraries were prepared for scRNA-seq. After quality control, 11,921 single cells were assigned an SBO using the D-score deconvolution method; 3,748 and 5,674 cells were assigned to the vehicle control and the irinotecan-treated spheroids, respectively. Cells were clustered and a clear separation between the vehicle control and drug-treated cells was observed, consistent with broad changes in gene expression induced by irinotecan treatment (Fig. 4a). A variety of relevant reactome pathways were significantly upregulated in irinotecan-treated spheroids (Supplementary Fig. S9).

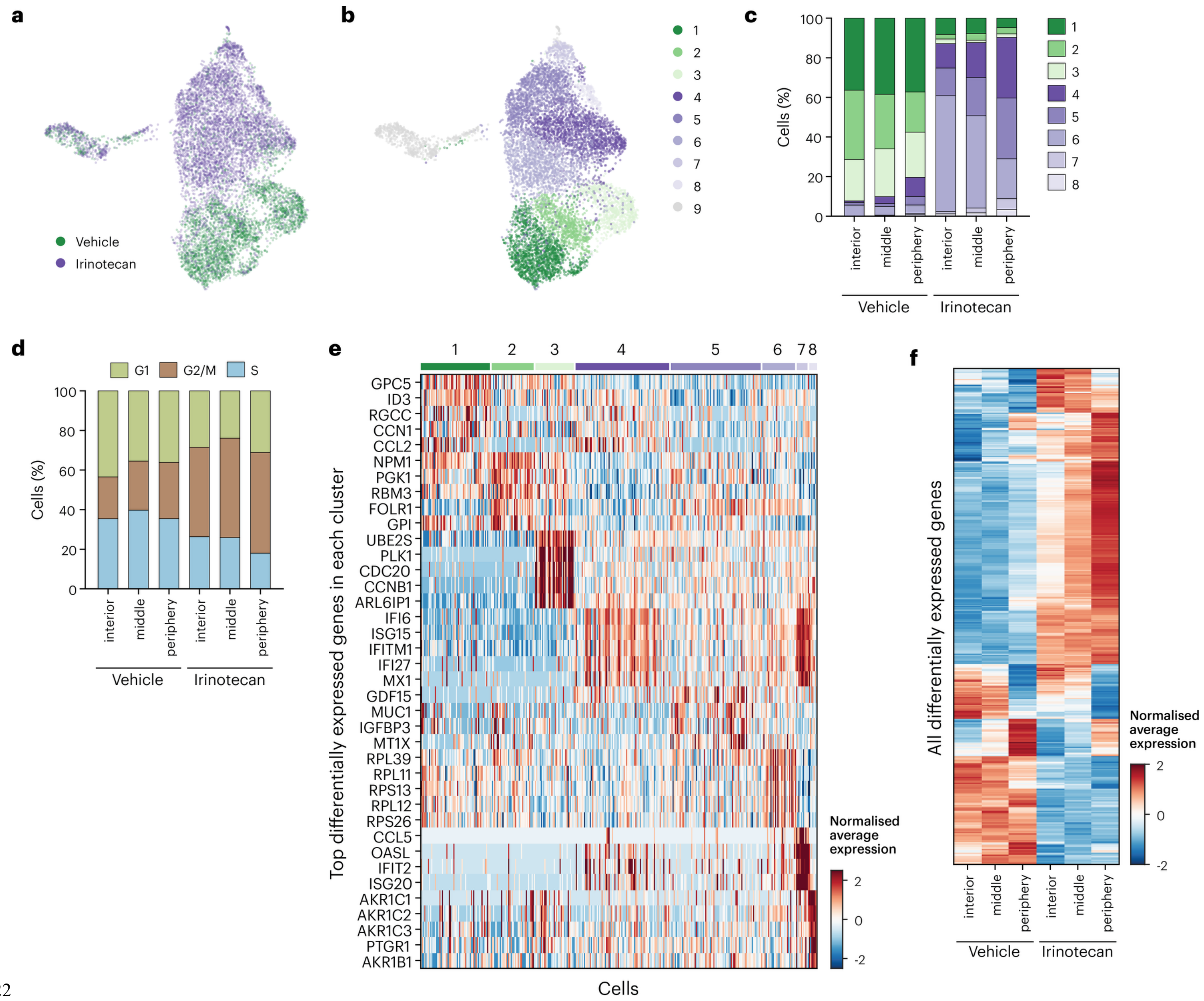

Figure 4. Irinotecan alters transcriptional profiles in multi-layer spheroids. (a) UMAP visualisation of single cells captured and sequenced. Cells are annotated according to whether they were treated with 0.1% DMSO ('vehicle') or 50 µM irinotecan for 24 h. **(b)** UMAP visualisation of single cells, annotated by cell cluster. **(c)** Distribution of cell clusters according to treatment with vehicle or irinotecan and layer. **(d)** Irinotecan increases expression of G2/M gene markers. Percentage of cells in each cell cycle from the vehicle control and irinotecan treated samples. **(e)** Heatmap enrichment of the top 5 DEGs ($|\log_2$ fold change$| > 0.25$ and $p_{adj} < 0.05$) in each cell cluster. **(f)** Normalised expression levels of DEGs ($|\log_2$ fold change$| > 0.25$ and $p_{adj} < 0.05$) between the vehicle and irinotecan treated spheroids, and each layer.

UMAP clustering revealed similar control populations as in the untreated spheroids, and an additional five clusters that were related to treatment with irinotecan (Fig. 4b). Within the three barcoded layers of these spheroids, we can identify different cell populations based on UMAP clustering of gene

expression profiles, suggesting that in future work using co-cultures or more complex cell models, different cell types or subpopulations could also be readily identified within spatially barcoded regions. One subpopulation in the dataset (cluster 9) was enriched in markers of cell death in the same way as for the two- and three-layer control spheroids and was again excluded. In the vehicle control, clusters were evenly distributed across the different layers, whereas the layers of the irinotecan-treated spheroids displayed greater differences in cell clustering (Fig. 4c). As an example, cluster 6 contained more cells from the interior region of the spheroids while the remaining clusters were enriched in cells from the periphery. We analysed the different layers for markers of cell cycle phase and found a general increase in G2/M markers in irinotecan-treated spheroids consistent with arrest at the G2 checkpoint (Fig. 4d). This agrees with the results in Fig. 3e-i in which cells are prevented from initiating mitosis.

We identified 3,970 DEGs between the eight clusters ($|\log_2$ fold change$| > 0.25$, $p_{adj} < 0.05$). Single-cell expression across the top 5 DEGs for each cluster is shown in Fig. 4e. Interestingly, cluster 8 displayed an upregulation in aldo/keto reductase (AKR) genes; within this family of stress-related genes, upregulation of *AKR1C3* in particular has been previously identified as a biomarker for irinotecan resistance in colon cancer cells.[31] Visualisation of the average gene expression across spheroid layers for all DEGs highlighted a variety of patterns, including an array of genes that were significantly upregulated compared to vehicle control in a spatially-dependent manner across spheroid layers following treatment with irinotecan (Fig. 4f).

scTECH-seq enables spatial analysis of irinotecan-induced change

The unique benefit of our multiplexing model is the ability to classify cells based on spatial location and map drug response across 3D systems. We filtered the average expression data for genes that did not vary in different layers of the control spheroids (to exclude baseline variation due to hypoxia, differences in metabolism, *etc.*). We identified 197 genes whose expression level was affected by irinotecan but did not significantly vary between layers (Supplementary Fig. S10), as well as 39 genes that presented a spatially dependent response to irinotecan (Fig. 5a). Within the set of spatially-dependent genes,

PARP14 has been associated with poor prognosis in pancreatic cancer[32], *IFITM2* has been identified as a marker of gastric cancer growth and metastasis[33], and *BCAP31* has been identified as a predictor of inferior survival in non-small cell lung cancer[34].

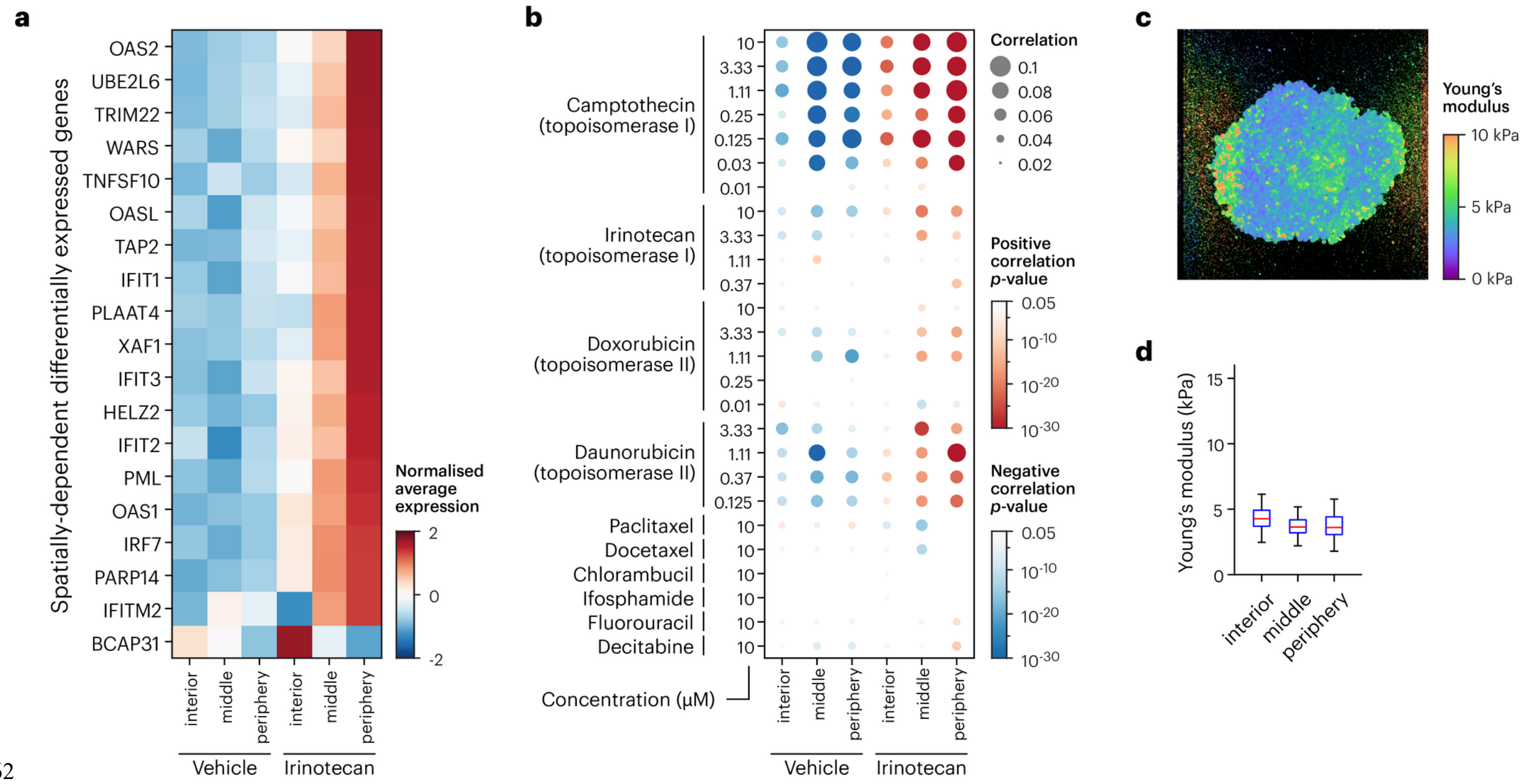

Figure 5. Irinotecan induces spatially dependent gene expression changes in spheroids. (a) Normalised average expression levels of DEGs, filtered for those that display a spatially dependent response to irinotecan while remaining consistent within untreated spheroids. **(b)** Gene expression profiles for each layer in vehicle and irinotecan-treated spheroids were correlated with 24 h HeLa perturbagen profiles from the LINCS L1000 database. Dot size represents Kendall's rank correlation coefficient τ, red dots show positive correlation, blue dots negative correlation, and colour scales represent *p*-value. **(c)** Mechano-microscopy was performed on three-layer spheroids, using fluorophore-tagged SBOs to identify boundaries between layers; the resulting map of Young's modulus shows that elasticity is relatively uniform throughout three-layer spheroids. **(d)** Spheroid elasticity quantified from the image in (c) did not significantly vary with depth, suggesting that gene expression differences between layers were not induced by variation in mechanical properties.

We correlated gene expression profiles from each layer of the vehicle and irinotecan-treated spheroids with gene expression profiles from the LINCS L1000 chemical response atlas (Fig. 5b).[35] LINCS datasets were all acquired in HeLa cells following 24 h drug treatment to match spheroid treatment conditions. Gene expression levels in spheroid layers were compared to L1000 expression signatures

through Kendall's rank correlation coefficient τ. We observed that the peripheral layer displayed the strongest positive correlations, which decreased in magnitude moving toward the interior, supporting the idea that drug activity was greatest within cells at the spheroid periphery, and that peripheral cells in our 3D culture most closely reflect the conditions under which cells are grown in 2D cultures. Interestingly, stronger positive correlations were observed with camptothecin than irinotecan, suggesting a different transcriptional response in 3D spheroids compared to 2D monolayer cultures, perhaps through differences in crowding, metabolic activity, senescence, and hypoxia. We also compared the expression profiles from our spheroids to other drugs; expecting to see some positive correlation with topoisomerase II inhibitors, we profiled against doxorubicin and daunorubicin, where we indeed found weaker positive correlations (Fig. 5b). As controls, we profiled against the taxanes paclitaxel and docetaxel, alkylating agents chlorambucil and ifosphamide, and antimetabolites fluorouracil and decitabine, all of which have different modes of action, and accordingly we found minimal similarity in gene expression profiles. Our results are consistent with the hypothesis that spatially encoded architectures can profile differential transcriptional responses to help explain evolving drug resistance and variation in cell response to drugs in a 3D context. With this proof-of-principle in hand, it would be invaluable to conduct more comprehensive studies with more drugs and drug classes, as well as spheroids of different cell composition and size, to further validate and compare gene expression profiles observed in 2D and 3D cultures, with a view toward establishing 3D spatiotemporal drug atlases.

Finally, we examined the mechanical properties of our spheroids using mechano-microscopy, a high-resolution variant of quantitative microelastography. We hypothesised that there may have been a discontinuous structure at spheroid layer boundaries because of stepwise growth, which could lead to variation in drug penetrance. Spheroids were embedded in hydrogels, and the deformation in response to an applied force was measured to obtain elasticity maps. Capitalising on the fluorophore-labelled SBOs present within each of the spheroid layers, we were able to assign elasticity measurements to each of the three regions. The multi-layered spheroids did not exhibit any signs of a disjoint boundary or change in elasticity between the layers (Fig. 5c,d). Thus, the spheroids were relatively homogeneous

in structure, and variation in the response to irinotecan was likely due to differences in drug penetration or cellular metabolic activity and not microenvironment. Interestingly, in comparison to our layer-by-layer assemblies, spheroids of a similar size grown in a single step displayed an elasticity that varied inversely with the cellular depth (Supplementary Fig. S10). We profiled a comprehensive set of mechanosensory receptors, adhesion molecules, and fibrotic markers within control spheroids (Supplementary Table S12), but except for *ACTG1*, which showed significantly higher expression in the spheroid interior compared to the periphery ($p_{adj} \leq 0.05$), no other genes exhibited spatial variation. This finding suggests minimal mechanosensory or adhesion-related heterogeneity in HeLa spheroids, consistent with the uniform elasticity observed by mechano-microscopy.

Discussion

In this work, we successfully used fluorophore-conjugated SBOs for intracellular barcoding in scRNA-seq experiments, enabling visualisation of barcoded cells. We anticipate that SBOs modified in this manner may also enable cell sorting based on SBO identity, for example, to clean up populations for cell types with non-uniform SBO uptake efficiency, or prior to sequencing to remove cells containing non-unique SBOs. We envision that assembly approaches could be extended beyond our layer-by-layer approach to automated 3D bioprinting to increase throughput and reproducibility. Polymer transfection by scTECH-seq is advantageous, because it results in complete cellular internalisation of SBOs via endocytosis. Alternative strategies such as CITE-seq employ lipid- or antibody-based anchoring of SBOs to the cell membrane, which is reversible and prone to barcode exchange between cells. scTECH-seq offers a number of advantages over existing sequencing methods, including compatibility with 5′ gene expression profiling, which offers better exonic read capture and long-read sequencing potential, and highly uniform barcode distribution that improves demultiplexing efficiency. Furthermore, the departure from chip-based 3′ mRNA capture allows us to multiplex samples to reduce cost, also facilitating experiments on a larger scale or with greater throughput in future. While existing spatial

transcriptomic platforms can achieve impressive resolution in planar tissues, scTECH-seq is uniquely suited for investigating transcriptional dynamics in complex, multilayered 3D cell culture models.

Our antigen-independent barcoding method (scTECH-seq) is sufficient for transfection, cell harvesting, assembly, and treatment of novel layered 3D HeLa architectures over 72–96 h. The labelled cell populations could be resolved by flow cytometry (Supplementary Fig. S3), and D-score demultiplexing assigned over 80% of cells unambiguously in three-layer spheroids, suggesting that intracellular delivery of SBOs by scTECH-seq results in minimal barcode diffusion between layers that is often observed using current lipid- or antibody-tagging technologies. We found evidence of reduced SBO numbers that would likely compromise successful demultiplexing after this timepoint, as revealed by SBO quantification over time (Supplementary Fig. S2). The fact that SBO abundance correlates with the cell population doubling time suggests that division is the primary determinant of SBO longevity rather than degradation. Since the number of demultiplexed cells correlated with the median number of SBO counts per cell (Supplementary Table S11), we concluded that some barcode loss or degradation had taken place over the longer time taken to grow three-layer spheroids, reducing demultiplexing efficiency in the interior and middle layers. However, we cannot exclude the possibility that cell death or stress in these layers may have also been a factor. Since polymer-based delivery of SBOs is known to result in endolysosomal entrapment, alternative delivery reagents to target alternative uptake routes[36], modifying SBOs with phosphorothioate linkages to reduce nuclease-mediated degradation[37], or generating cell lines with genetically encoded barcode sequences and/or fluorescent reporters[38] could help to preserve SBO integrity in future work. Furthermore, additional layers, greater numbers or multiplexing of fluorescent tags, or more rapid addition of layers to the growing spheroid to facilitate a more fine-grained analysis of spatial gene expression. It would be prudent to confirm cell viability when extending the culture to a greater number of layers or over a longer time period.

While spatial transcriptomics platforms are ideal for exploring gene expression over comparatively large tissue sections, spatial transcriptomics for spheroids is limited by the resolution of capture spots, which is generally 50–220 μm with a centre-to-centre gap of 100–500 μm. A previously reported

technique (SEEP-seq) for spatial transcriptomics in 3D cultures makes use of dye penetration to indirectly quantify cell depth in 3D cultures prior to single-cell analysis[39]. SEEP-seq provides information in relation to drug diffusivity, whereas our approach is more application-agnostic. We see complementary potential for SEEP-seq and our technique, which is not limited to diffusion, and could be used to profile aspects of 3D culture, including proliferation or epithelial–mesenchymal transition, which may not be directly related to diffusivity, especially in more complex architectures. An alternative to physical spatial transcriptomics platforms are computational statistical reconstruction methods that infer spatial position from droplet-based scRNA-seq data, such as novoSpaRc[40], SpaOTsc[41], and Tangram[42]. These approaches rely on matching gene expression profiles to a spatial reference atlas, or to each other, to reconstruct a pseudo-spatial map. While these techniques are powerful when high-quality spatial reference data are available, their accuracy can be limited in novel systems, cultures consisting of a single cell type, or engineered cultures lacking *a priori* spatial maps, as is the case here. In contrast, scTECH-seq provides a direct physical encoding of spatial origin, enabling spatially resolved single-cell transcriptomics in *de novo* 3D architectures without reliance on external spatial references. There is scope to incorporate statistical reconstruction methods in future, provided that the spatial fidelity of such techniques can be experimentally confirmed.

As we have demonstrated, SBOs not only facilitate multiplex scRNA-seq, but can also encode the spatial arrangement of cells, allowing scRNA-seq data to be related to cell location within engineered 3D cell culture architectures. Our data support the hypothesis that spheroid cultures display limited cell growth and proliferation within the core due to dense cell packing and a limited supply of nutrients and oxygen. Exploration of cellular response to irinotecan in the 3D context revealed a gradient in the transcriptomic response that indicated the greatest drug-related effects at the periphery. Extending the fluorescent barcoding idea further, the visualisation of SBOs within spheroids also supported *in situ* correlative imaging techniques; we demonstrated this possibility using mechano-microscopy to construct maps of elasticity (*i.e.,* Young's modulus) in 3D cultures. The ability to relate scRNA-seq sequencing data to matrix elasticity could facilitate correlative studies of gene expression in the context of mechanobiology.

While we validated spatially-resolved scTECH-seq here using HeLa spheroids, future work could explore applications in primary cells, co-culture systems, or organotypic models such as hepatic or neural spheroids to extend its utility in drug screening, toxicity testing, and developmental biology. We have previously applied scTECH-seq to HMLE and mesHMLE cells to study epithelial-to-mesenchymal transition in 2D cultures, which shows the applicability of our method to other cell types and cellular transitions, highlighting the versatility of this technology. In future applications involving stem cell differentiation or long-term culture, additional factors such as cellular migration, barcode stability, and dynamic gene expression changes would need to be considered; in such cases, stable genetic barcoding or chemically modified SBOs may be more suitable. However, slower-dividing cells, or studies involving terminal differentiation in which cells stop dividing, may support longer-term spatial barcoding in spheroids or developing organoids worthy of further exploration. While over long-term studies, migration of cells within a spheroid could result in mixing of spatially tagged cell populations, our use of fluorescent-tagged SBOs would allow any such migration of barcoded cell subpopulations to be visualised, while also supporting single-cell transcriptomic analysis. Thus, migration and differentiation could potentially be monitored simultaneously – another intriguing avenue for further study that is otherwise not possible with existing technologies. Future work could expand the drug panel to include compounds with diverse mechanisms of action to further demonstrate the utility of scTECH-seq in profiling spatially heterogeneous drug responses. We envisage that further correlative analysis between 2D and 3D drug response will be helpful in establishing expected gene expression profiles in 3D, and explaining heterogeneous activity and outcomes in response to drug treatment. Additionally, future studies could aim to quantify drug-induced TOP1 cleavage complexes within spheroid layers to directly link spatial transcriptomic profiles with the primary mechanism of action of TOP1 inhibitors, providing a more comprehensive understanding of spatial drug responses.

We conclude that transfection-based hashing methods such as scTECH-seq are compatible with the stepwise growth of layered spheroids. Fluorophore modification of SBOs enables cell subpopulations within the assembly to be imaged, and the use of unique SBO sequences facilitates multiplexing of scRNA-seq data to collect spatially-resolved, transcriptomic information. Our spatially-resolved data

highlights differences in gene expression that could be used to inform the first 3D gene expression atlases in response to drug treatment. Using scTECH-seq, the construction of engineered cellular architectures adds spatial resolution to studies of gene expression using *in vitro* 3D culture models.

Methods

SBO Barcode design

The 10X Genomics 5′ feature short barcode oligonucleotides (SBOs) were ordered from Integrated DNA Technologies (IDT Asia Pacific). SBOs (69 nt) were designed according to 10X Genomics specifications, except with the following modifications: oligonucleotides were modified by replacing the C_{12} amine linker with a shorter chain internal modified C (/iAmC6C/) and adding a fluorophore at the 5′ end (/5Cy5/ or /5Cy3/). Oligonucleotides were ordered at 250 nmol scale with HPLC purification. A representative SBO sequence using feature barcode 13 shown in lowercase is 5′-/5Cy3//iAmMC6C/GGAGATGTGTATAAGAGACAGNNNNNNNNNNataatcattacgtggNNNNNNNNNCCCATATAAGA*A*A-3′ (* = phosphorothioate). SBO sequences used in this study are presented in Supplementary Table S1.

Cell culture

HeLa cells were cultured in Dulbecco’s Modified Eagle Medium (DMEM) (Gibco 10569044) supplemented with 10% v/v FBS at 37 °C and 5% CO_2. HeLa cells were maintained at 37 °C in a humidified incubator with 5% CO_2 and passaged at 70–80% confluency using 1× TrypLE Express (Gibco).

Transfection protocol

Dendritic polymer for transfection of SBOs was synthesised as previously described[43,44] using a 25 mol% GMA backbone and generation 5.0 poly(amido amine) dendrons. Polymer and SBO complexes

were formed at amine-to-phosphate (N/P) ratio 30. For SBO labelling, cells were seeded at 150,000 cells/mL in a 24-well plate and incubated overnight at 37 °C and 5% CO_2. Polymer (3 mg/ml stock solution in ddH_2O) and SBO (100 μM stock solution in ddH_2O) were diluted in Opti-MEM (Gibco 31985070). SBO was diluted to 657 nM for a total of 23 pmol (1.59 nmol DNA phosphate groups) in 35 μL, while the polymer was diluted to 0.326 mg/ml for a total of 47.7 nmol primary amines in 35 μl. Equal volumes of polymer (34 μl) and SBO (34 μl) solutions were mixed gently but thoroughly and incubated for 25 min at room temperature. Cells were washed with 1×PBS once to remove serum and media was replaced with 150 μL Opti-MEM. 65 μL polymer–SBO complexes were added per well and cells were incubated at 37 °C for 4 h. Transfection efficiency was visualised with an epifluorescence microscope and quantified *via* flow cytometry.

Flow cytometry

For 2D cell cultures, transfection solution was removed and cells were washed with PBS twice. Cells were collected with TrypLE and 2× FACS buffer (2 mM EDTA + 6% FBS in 1×PBS) for flow cytometry. For spheroid dissociation, cells washed with PBS twice before incubation with TrypLE for 20 min at 37 °C. Spheroids were manually dissociated by pipette mixing 20–30 times until cell clumps were no longer visible. Cells were collected by centrifugation (300*g*, 5 min) and resuspended in 2× FACS buffer.

Spheroid formation

To form spheroids, transfected HeLa cells were plated in Nunclon Sphera 96-well U-shaped-bottom microplates (Thermo Scientific 174925). Spheroid formation was visualised under phase contrast microscopy. To grow three-layer spheroids, HeLa cells were transfected as described above. Transfected cells were harvested and plated in 96-well low adhesion plates at 2,000 cells/well in 100 μL media. To form the second layer, the following day, a new sample of HeLa cells was transfected with a different SBO prior to harvesting and 3,000 cells/well were seeded into fresh wells. Spheroids formed the previous day were transferred to the new well, with one spheroid per well. This process was

repeated on the third day, using 5,000 cells/well to form the final layer. Each day, cells were incubated for 24 h prior to the addition of the next layer. Two-layer spheroids were prepared the same way using 5,000 cells/well for both layers.

Spheroid dissociation and viability assay

Propidium iodide was used to assess the proportion of live/dead cells in spheroids after irinotecan treatment. 24 h after the third layer of cells were added, spheroids were treated with irinotecan. Irinotecan hydrochloride (Sigma-Aldrich I1406) was dissolved at 50 mg/mL in DMSO. Irinotecan was diluted in media to a 1 mM working stock and serial dilutions were used to make 200 μM, 100 μM, 40 μM, and 10 μM dilutions. Half of the volume in each well was removed and diluted irinotecan stocks were added in equal volume to achieve final concentrations of 100, 50, 20 and 5 μM. Spheroids were incubated for 24 h. Vehicle (0.1% v/v DMSO) and untreated spheroids were used as controls. After 24 h, spheroids (n = 8) for each treatment were transferred into a 1.5 mL tube. Media was removed and 300 μL 1× TrypLE Express was added, and spheroids were incubated for 15 min at 37 °C. After 15 min another 300 μL TrypLE was added to each tube and spheroids were pipetted up and down 20 times using a 1000 μL single-channel pipette until no visible cell clumps remained. Cells were centrifuged at 300*g* for 5 min and resuspended in 2× FACS buffer supplemented with 4 μg/mL propidium iodide solution. Cells were stored on ice until flow cytometry was performed (BD SORP Fortessa), using 561 nm laser and 610/20 nm bandpass filter. Data was analysed using FlowJo v10.

Spheroid sectioning

Following spheroid growth or drug treatment, spheroids were transferred into 1.5 mL tubes. Media was removed and 4% paraformaldehyde (PFA) in 1×PBS was added and spheroids were incubated for 20 min at 25 °C. Spheroids were washed with 1×PBS twice and incubated in 30% sucrose overnight at 4 °C for cryoprotection. The following day, spheroids were frozen in optimal cutting temperature (OCT) compound for cryosectioning using 12 μm slices.

Ki-67 immunofluorescence

Hydrophobic barrier pens were used to create a water-repellent barrier around spheroid sections. All incubation steps were conducted at 25 °C unless otherwise stated. Spheroid sections were fixed to slides with 4% w/v PFA for 15 min and washed with 1×PBS twice, 5 min each. Antigen retrieval was performed by immersing slides in citrate buffer (10 mM sodium citrate, pH 6.0 + 0.05% v/v Tween 20) overnight at 60 °C. Sections were washed with 1×PBS, permeabilised with 0.1% v/v Triton X-100 in 1×PBS for 10 min, followed by 2 × 5 min washes in 1× PBS. Blocking was conducted using 2% w/v BSA in 1×PBS for 1 h and incubated with anti-Ki67 antibody (Abcam ab16667) in a 1:200 dilution, overnight at 4 °C. Sections were washed 3 × 5 min in 0.1% v/v Tween 20 in 1×PBS. Secondary antibody incubation was left for 1 h using anti-rabbit Alexa Fluor 488-conjugated at a 1:500 dilution. Sections were washed with 0.1% v/v Tween 20 in 1×PBS twice and 1×PBS before mounting glass coverslips with Fluoromount G (Southern Biotech). Epifluorescence images were acquired (Nikon ECLIPSE Ti2) using a Plan Apo λ 10×/0.45 objective and analysed using ImageJ.

Fucci cells

Stable HeLa-Fucci cell line was generated using the Super PiggyBac Transposase Expression Vector system (System Biosciences, PB210PA-1) together with tFucci(CA)5 plasmid[30] (Addgene 153521), as per manufacturer's protocol. Briefly, HeLa cells were seeded at a density of 80,000 cells/mL in a 12-well plate and left to settle overnight (~16 h). For transfection, 200 ng of the PiggyBac transposase was mixed with 500 ng of tFucci(CA)5 plasmid (overall ratio of 1:2.5 transposase : transposon vector), and diluted to a total of 30 µL in Opti-MEM (Gibco 31985070). Separately, 0.7 µL Lipofectamine 2000 (Invitrogen 11668027) was diluted to 30 µL in Opti-MEM, and the two solutions were mixed thoroughly (total of 60 µL transfection cocktail) and incubated for 25 min at 25 °C. Meanwhile, cell media was removed, cells were rinsed twice with 1×PBS, and 400 µL Opti-MEM was added. After incubation, 60 µL of the transfection cocktail was added to the well, and cells were incubated at 37 °C for 3 h, after which 540 µL DMEM + 10% v/v FBS media was added to the well and the transfection

was left to proceed for a further 45 h (48 h total). After 48 h, the cells were transferred to a 10 cm plate and left overnight to settle. Cells were routinely cultured in selection media (DMEM + 10% v/v FBS + Blasticidin S HCl 10–30 µg/mL) until 100% of cells expressed the protein markers.

scRNA-seq sample preparation and sequencing

Three-layer spheroids were formed as described above. Spheroids were treated with 50 µM irinotecan or 0.1% DMSO for 24 h. Spheroids were dissociated as outlined in 'Spheroid dissociation and viability assay'. Single cells from the vehicle control and irinotecan spheroids were counted and pooled in equal number at 2,000 cell/µL in 0.4% w/v BSA for sequencing. Cell viability was confirmed to be >90%. Cells were loaded onto a 10x Genomics Chromium Controller, Chip K (1000286) and libraries were prepared using the Chromium Next GEM single cell 5′ kit v2 and 5′ Feature Barcode kit following manufacturer guidelines (10x Genomics 1000263, 1000265). Illumina iSeq 100 platform was used for initial QC of the libraries and deeper sequencing was conducted on Illumina NovaSeq 6000 in the vendor recommended format to reach up to 50,000 reads per cell. Fastq files were generated with Illumina bcl2fastq v2.20.0.422, allowing 1 mismatch in each index. 10x Genomics Cell Ranger was used for transcript alignment and generation of gene count and feature barcode matrices.

Single cell data analysis

The D-score deconvolution algorithm was used to assign SBOs to single cells (available at https://github.com/genomicswa/d-score). Three-layer spheroids are used here as an illustrative example. A total of 11,921 single cells were sequenced and successfully assigned an SBO. Cells were filtered for 200–95,000 total genes and less than 15% mitochondrial genes detected. 9,422 cells were used in downstream analysis. Data were normalised and the top 3,000 variable genes were selected for scaling. The scaled data was used for principal component, dimensional reduction and UMAP clustering. The Wilcoxon rank sum test was used to calculate DEGs ($|\log_2$ fold change$| > 0.25$, $p < 0.05$). DEGs were corrected for multiple testing using adjusted *p*-value. The following software versions were used: Cell Ranger v7.0.0, ClusterProfiler v4.6.2, GSEABase v1.60.0, ReactomePA v1.42.0, Seurat v4.3.0.1.

Mechano-microscopy

Mechano-microscopy was conducted as previously described[45]. Briefly, the mechano-microscopy system is built upon an optical coherence microscopy (OCM) setup using a supercontinuum laser for broadband light. The Michelson interferometer in the OCM system includes filters to create a spectral range of 650 nm to 950 nm and achieves ~1.4 µm axial resolution and ~0.5 µm lateral resolution. Two B-scans were acquired per location and B-scans were analysed to measure local displacement and strain, allowing for elasticity estimation using the stress–strain response of a pre-characterised compliant layer placed at the bottom of the sample. A confocal fluorescence microscopy system and a photomultiplier tube detector is integrated alongside the OCM to visualise fluorescent barcodes.

SBO inheritance and longevity analysis

For timelapse imaging to observe SBO inheritance by daughter cells, HeLa cells were seeded in 4-well chamber slides (Ibidi 80427) and incubated overnight before transfection. All quantities for transfection were scaled from a 24-well according to the culture surface area using SBO01-Cy5 and SBO13-Cy3. Cells were incubated with transfection mix for 2 h before adding complete media and imaging overnight. Epifluorescence and phase contrast images were captured every 10 min for 2 h, every 30 min for the following 6 h, and every 60 min thereafter for Cy3 and Cy5 (Nikon ECLIPSE Ti2, S Plan Fluor ELWD 20×/0.45 objective).

For SBO quantification over time by qPCR, HeLa cells were seeded at 0.3×10^6 cells per well in 6-well plates and incubated overnight before transfection. Two model barcode oligo sequences (SBO05: 5′-/5AmMC12/CGGAGATGTGTATAAGAGACAGCAGGNNNNNNTGGTGACAAGTATCTGAAC-CACGACCCATATAAGA*A*A-3′, SBO08: 5′-/5AmMC12/CGGAGATGTGTATAAGAGACAGC-AGGNNNNNNGCCAAGATCAGGTCCCAAAGGCAGCCCATATAAGA*A*A-3′; * represents phosphorothioate linkage) were ordered from Integrated DNA Technologies at 250 nmol scale with HPLC purification (NB: these oligos were modified slightly to facilitate PCR primer design and are therefore not suitable for sequencing experiments). Equal amounts of SBO05 and SBO08 were mixed

and diluted to a final concentration of 6.57 nM (*i.e.,* 3.29 nM each) in Opti-MEM (Gibco) and complexed with polymer at an N/P ratio of 30. Equal volumes of SBO and polymer were mixed and left to incubate at room temperature for 25 min to form transfection complexes. After rinsing the cells with PBS, 350 µL of the complexes were added to each well. Cells were incubated with the complexes at 37 °C for 4 h, after which 2 mL of complete medium were added without removing the transfection mixture. Samples were collected at 24, 72, 96, and 120 h post-transfection. All cells were washed once with PBS, pelleted, and stored at -80 °C until further use. Genomic DNA was extracted using the ISOLATE II Genomic DNA Kit (Bioline BIO-52067) following the manufacturer's protocol, with lysis carried out at 65 °C for 1 h. DNA was eluted twice in 50 µL of pre-warmed elution buffer. Concentration and purity were measured using NanoDrop Eight (Thermo Fisher Scientific). qPCR was performed using the QuantiNova SYBR Green PCR Kit (Qiagen 208056) on an AriaMx Real-Time PCR System (Agilent Technologies) in a 96-well plate format. Primers were ordered from Integrated DNA Technologies at 25 nmol scale with standard desalting (SBO forward: 5′-GGAGATGTGTATAAGA-GACAGCAGG-3′, SBO05 reverse: 5′-GTCGTGGTTCAGATACTTGTCACC-3′, SBO08 reverse: 5′-CTGCCTTTGGGACCTGATCTTG-3′, *AGTR1* forward: 5′-GGTTTCCACTGCCAAAACAATGC-3′, *AGTR1* reverse: 5′-CTAGGAAGACACTACTACTTGGGACC-3′). Reactions were set up in technical triplicates, amplifying the SBO sequence of interest, with *AGTR1* used as the internal reference gene for normalisation. The cycling protocol included an initial denaturation at 95 °C for 3 min, followed by 40 cycles of 95 °C for 15 s, 58 °C for 30 s, and 72 °C for 30 s. Melt curve analysis was performed after amplification to confirm primer specificity. Relative SBO levels were calculated using the comparative Ct (ΔΔCt) method. The fold change at each time point were calculated as $2^{-\Delta\Delta Ct}$ relative to the average ΔCt at the 24-hour time point, and error propagation was based on the standard deviation of ΔΔCt values from technical replicates.

Data and Code availability

The R code used to generate the plots presented in the main figures are available at https://github.com/Jessica736/Layer-by-layer-spheroids. The scRNA-seq datasets generated in this study have been deposited in the National Centre for Biotechnology Information (NCBI) Gene Expression Omnibus (GEO) under accession number GSE245416.

Author information

Corresponding author: Cameron W. Evans, cameron.evans@uwa.edu.au; Nicole M. Smith, nicole.smith@uwa.edu.au.

Author contributions

J.J.K. performed cell culture, barcoding, and spheroid experiments. M.N. synthesised and characterised dendritic polymers. J.A.K. produced Fucci reporter HeLa cells. J.J.K. and G.S. prepared and imaged spheroid sections. A.M., S.E.A., Y.S.C. and B.F.K. performed elasticity measurements. M.I., U.D.K. and A.S. processed cells for sequencing and performed initial bioinformatics processing of the dataset. J.A.K. and N.B. performed SBO longevity/inheritance experiments. J.A.K. analysed flow cytometry data in Fig. 3a, g-i. J.J.K. and C.W.E. processed and analysed the sequencing data and prepared figures. N.M.S., A.S. and C.W.E. conceived experiments. N.M.S. and C.W.E. supervised the project. N.M.S., K.S.I., and C.W.E. acquired funding. J.J.K. and C.W.E. wrote the draft manuscript. All authors contributed to proofreading and editing the manuscript.

Conflict of interest statement

None to declare.

Acknowledgements

This work was funded by the Australian Research Council and the State Government of Western Australia through the Future Research & Health Innovation Fund. We acknowledge the facilities and assistance at the Centre for Microscopy, Characterisation and Analysis at the University of Western Australia. Genomics WA is supported by BioPlatforms Australia, the State Government of Western Australia, Harry Perkins Institute of Medical Research, Cancer Research Trust, Telethon Kids Institute and the University of Western Australia. We gratefully acknowledge the Australian Cancer Research Foundation and the Centre for Advanced Cancer Genomics for making available Illumina Sequencers for the use of Genomics WA. We acknowledge the facilities, and the scientific and technical assistance of Microscopy Australia at the Centre for Microscopy, Characterisation & Analysis, The University of Western Australia, a facility funded by the University, State and Commonwealth Governments. J.A.K. acknowledges the Forrest Research Foundation for a Forrest Postdoctoral Fellowship.

References

1. Ingber, D. E. Human organs-on-chips for disease modelling, drug development and personalized medicine. *Nat Rev Genet* **23**, 467–491 (2022).

2. Wrzesinski, K. & Fey, S. J. From 2D to 3D--a New Dimension for Modelling the Effect of Natural Products on Human Tissue. *Curr Pharm Des* **21**, 5605–5616 (2015).

3. Greek, R. & Menache, A. Systematic Reviews of Animal Models: Methodology versus Epistemology. *Int. J. Med. Sci.* **10**, 206–221 (2013).

4. Pound, P. & Ritskes-Hoitinga, M. Is it possible to overcome issues of external validity in preclinical animal research? Why most animal models are bound to fail. *J Transl Med* **16**, 304 (2018).

5. Bracken, M. B. Why animal studies are often poor predictors of human reactions to exposure. *J R Soc Med* **102**, 120–122 (2009).

6. Van Norman, G. A. Limitations of Animal Studies for Predicting Toxicity in Clinical Trials. *JACC: Basic to Translational Science* **4**, 845–854 (2019).

7. Peng, W. *et al.* 3D bioprinting for drug discovery and development in pharmaceutics. *Acta Biomaterialia* **57**, 26–46 (2017).

8. Ryu, N.-E., Lee, S.-H. & Park, H. Spheroid Culture System Methods and Applications for Mesenchymal Stem Cells. *Cells* **8**, 1620 (2019).

9. Van De Sande, B. *et al.* Applications of single-cell RNA sequencing in drug discovery and development. *Nat Rev Drug Discov* **22**, 496–520 (2023).

10. Piwecka, M., Rajewsky, N. & Rybak-Wolf, A. Single-cell and spatial transcriptomics: deciphering brain complexity in health and disease. *Nat Rev Neurol* **19**, 346–362 (2023).

11. Ståhl, P. L. *et al.* Visualization and analysis of gene expression in tissue sections by spatial transcriptomics. *Science* **353**, 78–82 (2016).

12. Vickovic, S. *et al.* High-definition spatial transcriptomics for in situ tissue profiling. *Nat Methods* **16**, 987–990 (2019).

13. Merritt, C. R. *et al.* Multiplex digital spatial profiling of proteins and RNA in fixed tissue. *Nat Biotechnol* **38**, 586–599 (2020).

14. He, S. *et al.* High-plex imaging of RNA and proteins at subcellular resolution in fixed tissue by spatial molecular imaging. *Nat Biotechnol* **40**, 1794–1806 (2022).

15. Cougnoux, A. *et al.* Diffusion Smart-seq3 of breast cancer spheroids to explore spatial tumor biology and test evolutionary principles of tumor heterogeneity. *Sci Rep* **15**, 3811 (2025).

16. Baysoy, A., Bai, Z., Satija, R. & Fan, R. The technological landscape and applications of single-cell multi-omics. *Nat Rev Mol Cell Biol* **24**, 695–713 (2023).

17. King, J. J. *et al. Metastable Intermediates Identified in Epithelial to Mesenchymal Transition Are Regulated by G-Quadruplex DNA Structures*. http://biorxiv.org/lookup/doi/10.1101/2023.08.21.554220 (2023) doi:10.1101/2023.08.21.554220.

18. Mowla, A. *et al.* Subcellular mechano-microscopy: high resolution three-dimensional elasticity mapping using optical coherence microscopy. *Opt. Lett.* **47**, 3303 (2022).

19. Shin, D., Lee, W., Lee, J. H. & Bang, D. Multiplexed single-cell RNA-seq via transient barcoding for simultaneous expression profiling of various drug perturbations. *Sci. Adv.* **5**, eaav2249 (2019).

20. Nunes, A. S., Barros, A. S., Costa, E. C., Moreira, A. F. & Correia, I. J. 3D tumor spheroids as in vitro models to mimic in vivo human solid tumors resistance to therapeutic drugs. *Biotech & Bioengineering* **116**, 206–226 (2019).

21. Iwai, T. *et al.* Topoisomerase I inhibitor, irinotecan, depletes regulatory T cells and up-regulates MHC class I and PD-L1 expression, resulting in a supra-additive antitumor effect when combined with anti-PD-L1 antibodies. *Oncotarget* **9**, 31411–31421 (2018).

22. Shitara, K. *et al.* Randomised phase II study comparing dose-escalated weekly paclitaxel vs standard-dose weekly paclitaxel for patients with previously treated advanced gastric cancer. *Br J Cancer* **110**, 271–277 (2014).

23. Kindler, H. L., Herndon, J. E., Zhang, C., Green, M. R., & Cancer and Leukemia Group B. Irinotecan for malignant mesothelioma A phase II trial by the Cancer and Leukemia Group B. *Lung Cancer* **48**, 423–428 (2005).

24. Perez, E. A. *et al.* Randomized phase II study of two irinotecan schedules for patients with metastatic breast cancer refractory to an anthracycline, a taxane, or both. *J Clin Oncol* **22**, 2849–2855 (2004).

25. Xu, Y. & Villalona-Calero, M. A. Irinotecan: mechanisms of tumor resistance and novel strategies for modulating its activity. *Annals of Oncology* **13**, 1841–1851 (2002).

26. Gilbert, D. C., Chalmers, A. J. & El-Khamisy, S. F. Topoisomerase I inhibition in colorectal cancer: biomarkers and therapeutic targets. *Br J Cancer* **106**, 18–24 (2012).

27. Rivory, L. Molecular, cellular, and clinical aspects of the pharmacology of 20(S)camptothecin and its derivatives. *Pharmacology & Therapeutics* **68**, 269–296 (1995).

28. De Man, F. M., Goey, A. K. L., Van Schaik, R. H. N., Mathijssen, R. H. J. & Bins, S. Individualization of Irinotecan Treatment: A Review of Pharmacokinetics, Pharmacodynamics, and Pharmacogenetics. *Clin Pharmacokinet* **57**, 1229–1254 (2018).

29. Sakaue-Sawano, A. *et al.* Genetically Encoded Tools for Optical Dissection of the Mammalian Cell Cycle. *Molecular Cell* **68**, 626-640.e5 (2017).

30. Ando, R., Sakaue-Sawano, A., Shoda, K. & Miyawaki, A. Two coral fluorescent proteins of distinct colors for sharp visualization of cell-cycle progression. *Cell Struct. Funct.* **48**, 135–144 (2023).

31. Matsunaga, T. *et al.* Significance of aldo-keto reductase 1C3 and ATP-binding cassette transporter B1 in gain of irinotecan resistance in colon cancer cells. *Chemico-Biological Interactions* **332**, 109295 (2020).

32. Yao, N., Chen, Q., Shi, W., Tang, L. & Fu, Y. PARP14 promotes the proliferation and gemcitabine chemoresistance of pancreatic cancer cells through activation of NF-κB pathway. *Molecular Carcinogenesis* **58**, 1291–1302 (2019).

33. Xu, L. *et al.* IGF1/IGF1R/STAT3 signaling-inducible IFITM2 promotes gastric cancer growth and metastasis. *Cancer Lett* **393**, 76–85 (2017).

34. Wang, J. *et al.* BCAP31, a cancer/testis antigen-like protein, can act as a probe for non-small-cell lung cancer metastasis. *Sci Rep* **10**, 4025 (2020).

35. Duan, Q. *et al.* L1000CDS2: LINCS L1000 characteristic direction signatures search engine. *npj Syst Biol Appl* **2**, 16015 (2016).

36. Dunn, A., Cai, Y., Iwasawa, K., Kimura, M. & Takebe, T. POLYseq: A poly(β-amino ester)-based vector for multifunctional cellular barcoding. *Stem Cell Reports* **16**, 2149–2158 (2021).

37. Sago, C. D. *et al.* Barcoding chemical modifications into nucleic acids improves drug stability *in vivo*. *J. Mater. Chem. B* **6**, 7197–7203 (2018).

38. Guo, C. *et al.* CellTag Indexing: genetic barcode-based sample multiplexing for single-cell genomics. *Genome Biol* **20**, 90 (2019).

39. Morse, D. B. *et al.* Positional influence on cellular transcriptional identity revealed through spatially segmented single-cell transcriptomics. *Cell Systems* **14**, 464-481.e7 (2023).

40. Nitzan, M., Karaiskos, N., Friedman, N. & Rajewsky, N. Gene expression cartography. *Nature* **576**, 132–137 (2019).

41. Cang, Z. & Nie, Q. Inferring spatial and signaling relationships between cells from single cell transcriptomic data. *Nat Commun* **11**, 2084 (2020).

42. Biancalani, T. *et al.* Deep learning and alignment of spatially resolved single-cell transcriptomes with Tangram. *Nat Methods* **18**, 1352–1362 (2021).

43. Kretzmann, J. A. *et al.* Synthetically controlling dendrimer flexibility improves delivery of large plasmid DNA. *Chem. Sci.* **8**, 2923–2930 (2017).

44. Evans, C. W. *et al.* Intracellular Communication between Synthetic Macromolecules. *J. Am. Chem. Soc.* **144**, 14112–14120 (2022).

45. Mowla, A. *et al.* Multimodal mechano-microscopy reveals mechanical phenotypes of breast cancer spheroids in three dimensions. *APL Bioengineering* **8**, 036113 (2024).

Reviewer #1

The authors replied to all my comments.

- ✓ We thank the reviewer for their contributions.

Reviewer #2

Edits to the manuscript are highlighted in yellow.

1. First, I acknowledge the improvements made in addressing Comment 1 (barcode distribution and contamination) and Comment 8 (FACS-based quantification of SN-38 / Fucci). These changes appropriately correct some of the overstatements in the original submission and strengthen the discussion of feasibility and analytical framework.

   - ✓ We appreciate the reviewer's recognition of these revisions.

2. However, I am frankly surprised by the absence of any new experimental evidence for several critical points. Specifically, issues raised in Comment 2 (cell types), Comment 3 (drug types), Comment 4 (RNA spatial localization), Comment 9 (counterpart data using inhibitors), and Comment 10 (mechanobiology-related observations) were only addressed by softening the language, without providing additional data. While I understand the authors' reasoning, this approach substantially weakens the claim of methodological generalizability.

   - ✓ We have responded separately to each of these points below. Our revisions here focus on clarifying scope and ensuring claims remain appropriately calibrated to the presented data.

     i. *Additional cell types:* Our primary contribution here is the spatially encoded spheroid assembly strategy rather than biology specific to any cell line. Reports of controlled spheroid architectures are rare in the literature, and assembly of transfected or barcoded cells into defined 3D assemblies even more so. However, our layer-by-layer technique will not be applicable to every cell type. Many cell types and assembly methods could be implemented, since 3D cellular assemblies can be created with different methods (such as printing, microfluidics, drop-based methods, *etc.* as reviewed in doi:10.3390/ijms21176225), resulting in different cellular arrangements. As for the sequencing technique itself, we reaffirm our *bioRxiv* preprint, doi:10.1101/2023.08.21.554220v1, provides validation of scTECH-seq in human mammary luminal epithelial cells and a stable mesenchymal variant of the same, and we have successfully implemented scTECH-seq in primary human fibroblasts originating from the mesoderm (unpublished). To avoid implying unsupported generalisability in this manuscript, we have moderated and clarified relevant statements:

        > ***Line 31***
        > *"is a general approach that can resolve spatial variation in gene expression" → "may help to reveal spatial variation in gene expression..."*
        > ***Lines 28, 415***
        > *Specified HeLa rather than general cell types.*
        > ***Line 390***
        > *"While we validated spatially-resolved scTECH-seq here using HeLa spheroids, future work could explore applications in other immortalised cell types..."*

     ii. *Additional drug types and counterpart data using inhibitors:* Irinotecan was selected specifically because the fluorescence of its active metabolite, SN-38, confirms intracellular localisation, not just passive coexistence with cells, and enables quantification of intracellular 3D distribution. Other drugs would not provide a comparable intracellular visual validation and would require orthogonal methods to validate cellular uptake. In terms of comparisons, scTECH-seq enabled spatially resolved transcriptional profiling of drug response in 3D, where irinotecan-induced gene expression

signatures in the peripheral spheroid layer closely matched the 2D HeLa LINCS reference data for topoisomerase I inhibitors, while signatures from the middle and interior layers increasingly diverged from the observed 2D gene expression profile. The metabolite of irinotecan, SN-38, is a structurally related camptothecin analogue (7-ethyl-10-hydroxycamptothecin). Hence, the gene expression correlation between camptothecin in 2D and irinotecan in 3D is expected. Future studies of drugs with distinct mechanisms and physicochemical properties would indeed further establish the generalisability of these findings, but represents a much larger and more costly undertaking.

iii. In situ *validation:* Please refer to point 3 below.

iv. *Mechanobiology-related observations:* We examined a large panel of mechanobiology-related genes as suggested and observed minimal expression differences, with only a single gene showing statistical significance across layers. This is consistent with expectations given the layer-by-layer assembly method, which does not appear to impose strong mechanical gradients. Mechanobiology-focused studies are better suited to models with tighter contacts and greater physical stress.

3. The lack of in situ RNA validation (Comment 4) is particularly concerning, as these omissions leave key aspects of the dataset unvalidated. This raises questions about the robustness of the conclusions and feels somewhat inconsistent with the emphasis placed on these points in the original manuscript.

✓ We highlight that our experimental transcriptional data were corroborated against the independently-generated LINCS L1000 transcriptomic database. The strong concordance between peripheral and middle layer signatures and the established topoisomerase I inhibitor profile in LINCS already constitutes meaningful orthogonal benchmarking against an independent dataset.

✓ We did attempt orthogonal validation of some identified markers at the protein level, but spatial variation in mRNA did not translate to protein-level heterogeneity within the timeframe of our analysis. This is consistent with the idea that single-cell transcriptomics captures spatial gradients that lower-throughput imaging approaches cannot resolve, strengthening the novelty of the manuscript. Additionally, *in situ* hybridisation is unlikely to provide clear layer-specific patterns due to substantial cell-to-cell expression variability despite obvious differences in average expression between layers, making spatial patterns challenging to visualise. We have updated to explain that while further validation may be pursued in more physiologically relevant models, imaging methods may not reliably resolve subtle inter-layer expression differences.

> ***Lines 261–264***
> *Further validation of the above genes could be undertaken in more physiologically applicable models of the relevant cancers. However, we note that in situ hybridisation and immunofluorescence may not clearly reveal differences between layers due to relatively high cell-to-cell variation in gene expression.*

4. Regarding Comments 5 and 6 on spatial resolution: I appreciate the authors' acknowledgment of limitations in spheroid or organoid contexts. Nevertheless, published studies demonstrate that spatial transcriptomics can be applied even at the spheroid scale, with internal population structures resolved to a meaningful degree (see PNAS 2024, Nature 2023). While scTECH-seq may capture 3D layers within spheroids, section-based approaches could likely achieve similar resolution.

✓ We appreciate the reviewer's point and have further moderated our claims regarding achievable resolution. Language in the Abstract and Discussion has been refined to ensure precision and avoid overstating capability. We note that the cited *PNAS* (2024) study relies on custom laminated sample preparation with associated technical hurdles, and that this study really highlights some of the practical challenges motivating our approach.

> ***Line 53***
> *The reference to PNAS (2024) has been added.*

✓ We also reiterate that benchmarking of scTECH-seq and D-score demultiplexing is presented in a separate preprint (doi:10.1101/2023.08.21.554220v1).

5. If the authors intend to claim single-cell resolution in Fig. 4a–c (Comment 7), benchmarking against a competing section-based technology would be highly valuable, even if ambitious.

   - ✓ We do not claim single-cell resolution in Fig. 4a–c. The text refers to identification of cell types or subpopulations within spatially barcoded regions, not individual cells. We have reviewed wording to ensure this distinction is clear.

6. In summary, the key question is whether the toned-down novelty claims sufficiently compensate for the lack of benchmarking and validation. At present, these gaps remain significant and will likely influence the decision.

   - ✓ Drug-induced gene expression signatures recovered by scTECH-seq from discrete spheroid layers were independently corroborated against the LINCS L1000 drug perturbation database – a large, well-established 2D reference dataset. The strong correlation between peripheral/middle layer signatures and topoisomerase I inhibitor reference data confirms that scTECH-seq accurately captures spatially resolved transcriptional responses consistent with known drug biology. This orthogonal validation against an independent, externally generated dataset demonstrates high-fidelity transcriptional data that compares favourably against existing datasets. We have adjusted novelty claims accordingly, clarified limitations, and outlined areas for future work while ensuring the manuscript transparently communicates its scope and contributions.

Reviewer #3, 4

I co-reviewed this manuscript with one of the reviewers who provided the listed reports. This is part of the Nature Communications initiative to facilitate training in peer review and to provide appropriate recognition for Early Career Researchers who co-review manuscripts.

- ✓ We thank Reviewers 3 and 4 for their contributions.

Reviewer #5

The major concerns raised in the previous review have been clearly and satisfactorily addressed. In particular, the advantages of the layered spheroid model are now more clearly articulated and effectively highlighted. The earlier confusion regarding the relative sizes of the two-layer and three-layer spheroids—stemming from an incorrect scale bar—has been appropriately corrected. With this issue resolved, it is now evident that the spheroids are comparable in size, which greatly improves the clarity and interpretability of the layered spheroid approach. The additional clarifications concerning spheroid growth dynamics and patterns of cell death throughout development further strengthen the manuscript. The observation that irinotecan treatment exerts differential effects between the interior and peripheral regions of the spheroid is particularly intriguing. While the precise underlying mechanisms remain to be fully defined, this does not diminish the overall value or utility of the model. Overall, the manuscript has been substantially improved, and we have no remaining major concerns.

- ✓ We thank the reviewer for their contributions.
